# Target Isolation of Prenylated Isoflavonoids and Pterocarpans from *Acosmium diffusissimum* Using LC–MS/MS-Based Molecular Networking

**DOI:** 10.1021/acsomega.5c00866

**Published:** 2025-03-27

**Authors:** Gabriela Ribeiro de Sousa, Natanael Ramos de Lima Teles, Carlos Vinicius Azevedo da Silva, Mariana Costa Aragão, Domingos Benício Oliveira Silva Cardoso, Francisco Allysson Assis Ferreira Gadelha, Marcia Regina Piuvezam, Josean Fechine Tavares, Marcelo Sobral daSilva, José Maria Barbosa Filho

**Affiliations:** † Laboratório Multiusuário de Caracterização e Análises, Programa de Pós-Graduação em Produtos Naturais e Sintéticos Bioativos, Centro de Ciências da Saúde, Universidade Federal da Paraíba, João Pessoa 58051-900, Paraíba, Brazil; ‡ Grupo de Pesquisa em Metabolômica e Espectrometria de Massas, Universidade Estadual do Amazonas (UEA), 690065-130, Manaus, Amazonas, Brazil; § Instituto de Biologia, Universidade Federal da Bahia, Salvador 40170-115, Bahia, Brazil; ∥ Laboratório de Imunofarmacologia, Programa de Pós-Graduação em Produtos Naturais e Sintéticos Bioativos, Centro de Ciências da Saúde, Universidade Federal da Paraíba, João Pessoa 58051-900, Paraíba, Brazil

## Abstract

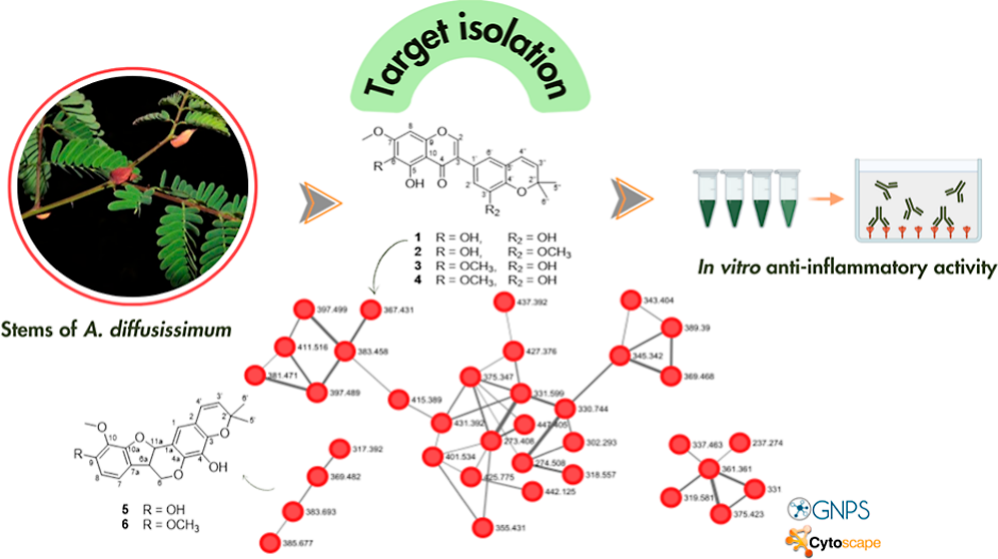

This is the report of the HPLC-MSn-guided isolation of new anti-inflammatory prenylated isoflavonoids and pterocarpans from the stems of *Acosmium diffusissimum* using GNPS molecular networking as the main tool. Cluster analysis guided the isolation of five new prenylated isoflavonoids, diffusiflavone A–E (**1–4** and **9**), two prenylated pterocarpans, diffusicarpan A and B (**5** and **6**), and two known compounds 6-prenylorobol (**7**) and 3-*O*-methylquercetin (**8**). The in vitro anti-inflammatory potential of compounds **1–6** and **9** was assessed in macrophages induced with lipopolysaccharide (LPS). Compounds **2**, **3**, **5**, and **9** were observed to have a reduction in NO levels in at least one of the concentrations tested (1.25, 2.5, 5, 10, and 20 μg/mL) and also reduced IL-1β and IL-6 cytokines, especially diffusicarpan A which reduced cytokine levels in all the concentrations tested.

## Introduction

Inflammation is a physiological response of the immune system aimed at defending the organism against various agents such as pathogens, toxins, and injuries in order to return to homeostasis.^1^ Dysregulation of this process can lead to various disorders, including metabolic, cardiovascular, neuropsychiatric, and neurological diseases.^2−4^

Natural products derived from plants are important sources of new bioactive compounds. Plants from the Fabaceae–Papilionoideae family have a large chemical diversity and high biological potential^5,6^ with a notable occurrence of isoflavonoids and pterocarpans which have anti-inflammatory activity among their many associated pharmacological activities.^7−11^*Acosmium diffusissimum* (Mohlenbr.) Yakovlev (Fabaceae–Papilionoideae) is a species endemic to Brazil, occurring in the states of Minas Gerais and Bahia in the caatinga regions. Despite the existing pharmacological potential of species within *Acosmium sect. Acosmium* (clade Dalbergieae), their chemistry and biological potential is still poorly explored.

Molecular networking is one of the most explored features of Global Natural Product Social Molecular Networking (GNPS). The MS/MS data obtained experimentally are converted, added to the online platform, and compared with internal spectral libraries showing a chemical relationship of similarity between the compounds which are arranged in molecular networking. For this reason, the use of this tool has been reported as one of the main tools in dereplication studies, a strategy adopted to target the discovery of new compounds.^12,13^

Natural products are promising sources of anti-inflammatory compounds due to their structural diversity and innovative mechanisms of action. Flavonoids isolated from *Kalanchoe brasiliensis* have demonstrated significant anti-inflammatory activity, reinforcing their therapeutic potential.^14^ Similarly, vermelhotin, a fungal metabolite, inhibited nitric oxide production in RAW 264.7 macrophages via suppression of the p38 MAPK pathway.^15^ These findings highlight the importance of natural metabolite prospecting as a strategic approach for the discovery of new anti-inflammatory agents.

**Figure 1:**
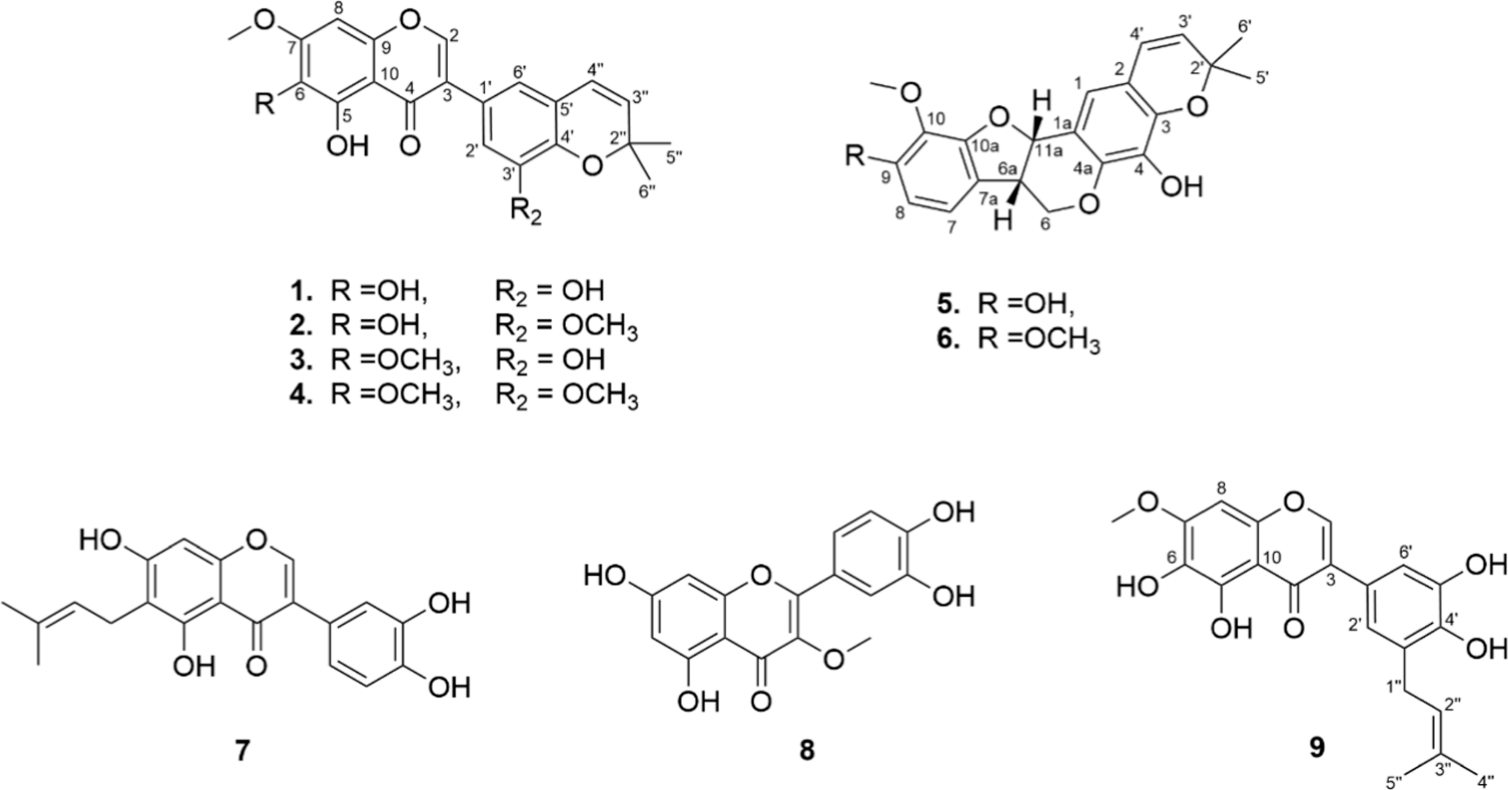
Compounds from the stems of *A. diffusissimum*.

In this work, we report the application of GNPS molecular networking to guide the isolation of five new prenylated isoflavonoids (**1–4** and **9**) and two prenylated pterocarpans (**4** and **5**) (Figure 1) and their anti-inflammatory activity by evaluating inflammatory mediators (NO, IL-1β, and IL-6) in LPS-induced macrophages, as well as the isolation of two already known compounds 6-prenylorobol (**7**) and 3-*O*-methylquercetin (**8**) from the stems of *A. diffusissimum*.

## Results and Discussion

**Figure 2:**
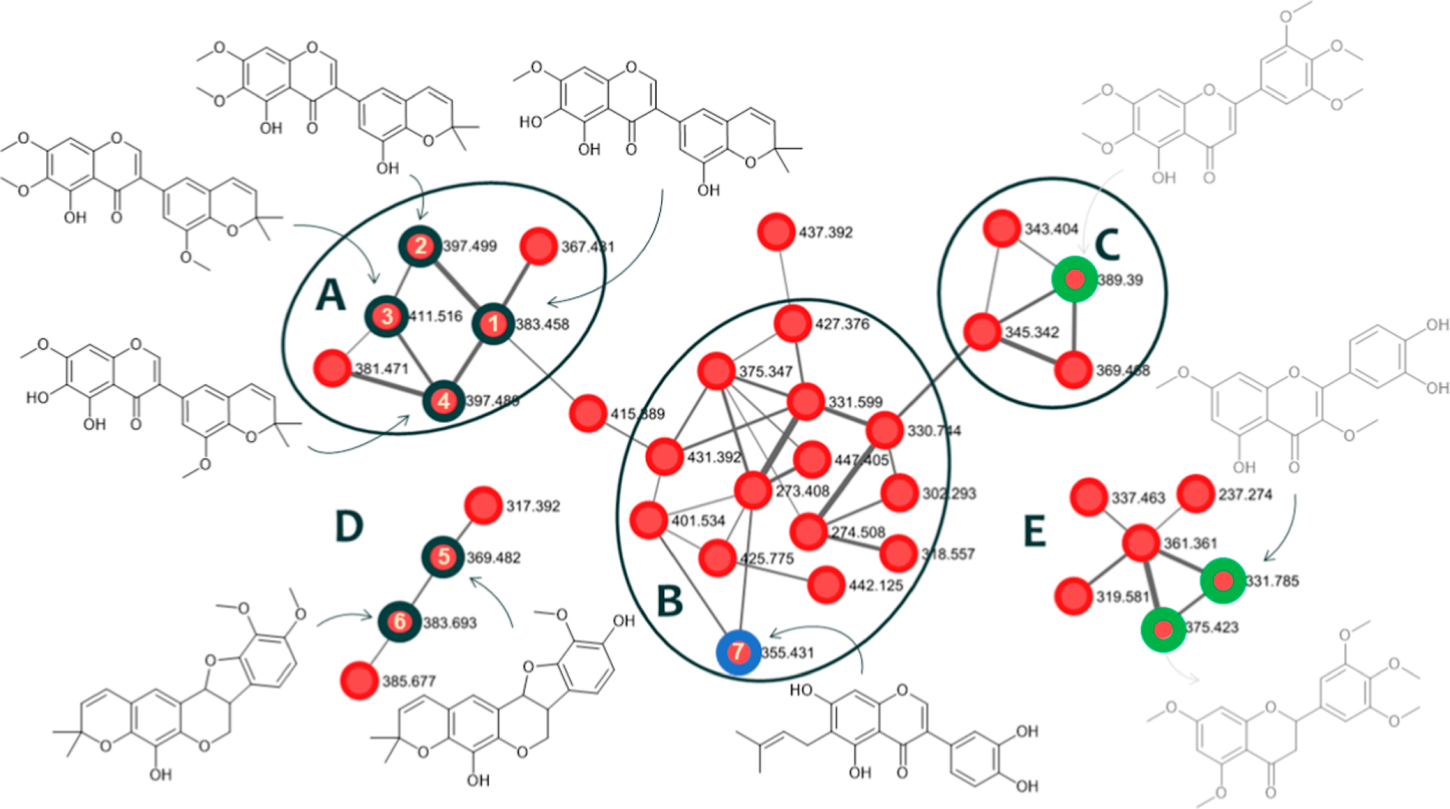
Molecular networking of the flavonoid-rich fraction of *A. diffusissimum* stems. Cluster A and B (prenylated isoflavonoids), Cluster C (polymethoxylated flavonoids), Cluster D (pterocarpans), and Cluster E (flavonoids). Nodes with bold borders were isolated and characterized substances and identified with their respective numbers, nodes with blue borders were substances annotated and confirmed with an internal standard (substances isolated and characterized from *A. diffusissimum* stems) and nodes with green borders were annotated substances.

The flavonoid-rich fraction from the stems of *A. diffusissimum* was analyzed by LC–MS/MS in positive mode, and its data were processed using GNPS. The molecular networking obtained showed 32 clusters and 83 nodes. The clusters were evaluated according to their combination with the spectral library, retention times, and comparison of the LC–MS/MS data with the literature. The nodes are connected by lines whose thickness is directly related to the cosine values, where thicker lines correspond to a higher cosine value and therefore greater similarity between the nodes. Based on this, the compounds were compiled into clusters A and B (prenylated isoflavonoids), C (polymethoxylated flavonoids), D (pterocarpans), and E (flavonoids) (Figure 2).

Cluster A exhibited a node at *m*/*z* 367 which showed a base peak ion in the MS^2^ spectrum of *m*/*z* 183. The MS^3^ spectrum of the *m*/*z* 183 fragment ion showed product ions of *m*/*z* = 165 (–H_2_O) and *m*/*z* = 155 (−CO). The fragmentation pattern for precursor ion *m*/*z* 367 [M + H]^+^ was not found in the literature. However, evaluation of the hit library match from the GNPS database revealed a structural similarity with the compound corylin, an isoflavonoid with a pyran ring inserted into its basic skeleton, whose fragmentation pattern with a base peak ion in the MS^2^ spectrum is obtained by an RDA fragmentation reaction of the C ring.^16,17^ This information suggested that the nodes present in this cluster could be compounds not yet described for this class, and the isolation of these compounds was prioritized.

The nodes at *m*/*z* 383 and *m*/*z* 397 and their respective adducts [M + H + Na]^+^ 405 and [M + H + Na]^+^ 419 shared fragment ions in the MS^2^ spectrum at *m*/*z* 365 and *m*/*z* 337. These fragments were generated from losses of H_2_O (18 Da) and CO + H2O (46 Da) and neutral losses of HOCH_3_ (32 Da) followed by the loss of CO (28 Da), for precursor ions *m*/*z* 383 and *m*/*z* 397, respectively (Schemes S1 and S2). The product ion of *m*/*z* 183 (RDA fragmentation)^18−24^ was also observed for both compounds, but with intensities of 100 and 49%, respectively (Figures S2 and S12).

In the MS^2^ spectrum of precursor ion at *m*/*z* 397, the base ion was observed at *m*/*z* 382, corresponding to the loss of a methyl group (15 Da), a feature absent in the ion at *m*/*z* 383. The product ion was also identified at *m*/*z* 200, indicating cleavage of the C ring by RDA after radical elimination. These data confirm that radical elimination occurs due to the presence of a methoxyl at C-3. In the MS^3^ spectrum of *m*/*z* 382, the following fragment ions were detected at *m*/*z* 346 (−2H_2_O), *m*/*z* 364 (−H_2_O), *m*/*z* 200 (RDA and –CH_3_), and *m*/*z* 155 (RDA and –CO). On the other hand, the MS^3^ spectrum of the ion at *m*/*z* 383 showed a loss of 32 Da (−HOCH_3_), giving rise to the fragment ion at *m*/*z* 151.

The nodes at *m*/*z* 397 and *m*/*z* 411 showed common fragment ions at *m*/*z* 379 (–H_2_O and –HOCH_3_, respectively) and *m*/*z* 351 (−CO + H_2_O and –CO + HOCH_3_), as well as the same RDA pathway product ion at *m*/*z* 197, with relative intensities of 100% and 50%, respectively (Figures S23 and S33). In the MS^2^ spectrum of the precursor ion at *m*/*z* 411, the base ion was identified at *m*/*z* 396, resulting from a radical loss of 15 Da (−CH_3_). The MS^3^ spectrum suggests that the ions at *m*/*z* 363 and *m*/*z* 200 (after RDA) are also dystonic ions, representing a fragmentation pathway that differentiates the *m*/*z* 411 ion from its analogue at *m*/*z* 397.

Also, in the MS^3^ spectrum of the ion at *m*/*z* 411, fragment ions were observed at *m*/*z* 378 (−H_2_O), *m*/*z* 363 (−H_2_O and –CH_3_), *m*/*z* 197 (RDA), and *m*/*z* 179 (RDA and loss of –H_2_O). In contrast, in the MS^3^ spectrum of the precursor ion at *m*/*z* 397, fragments were observed at *m*/*z* 197 (RDA), *m*/*z* 182 (RDA and loss of –CH_3_), and *m*/*z* 151 (RDA followed by loss of –CO and –H_2_O) (Scheme S5).

Cluster D showed nodes at *m*/*z* 369 and *m*/*z* 383 which shared neutral losses of 164 Da, generating the base peak ions in the MS^2^ spectrum at *m*/*z* 205 and *m*/*z* 219, respectively. The MS^2^ spectrum of the ion at *m*/*z* 369 also showed product ions of *m*/*z* 341, *m*/*z* 177, and *m*/*z* 153. The MS^3^ spectrum showed a common fragment ion at *m*/*z* 187 for both precursor ions, as well as product ions at *m*/*z* 201 (−H_2_O) and *m*/*z* 147 (−CH3 and –OC(CH_3_)_2_) for the ion at *m*/*z* 383. Analysis of the fragmentation pattern of this cluster with data in the literature indicated the presence of pterocarpans with a dimethyl pyran ring.^23−25^ On the other hand, there was no correlation between the LC–MS/MS data and the literature, so these compounds were also prioritized for guided isolation.

The flavonoid-rich fraction obtained from the stems of *A. diffusissimum* was fractionated and monitored by LC–MS using the fragmentation pattern and UV absorption profile. The guided isolation allowed us to isolate and characterize five prenylated isoflavonoids (**1–4** and **9**) and two pterocarpans (**5** and **6**) and known compounds 6-prenylorobol (**7**)^26−28^ and 3-*O*-methylquercetin (**8**),^31,32^ which were used as internal standards to confirm the nodes noted on the molecular networking. In continuing the study of the chemical profile of *A. diffusissimum*, fractionation of higher-polarity fractions was also carried out and it was possible to isolate and characterize compound **9**, demonstrating the potential of *A. diffusissimum* as a source for obtaining other compounds of this class.^29,30^

Compound **1** was obtained as a yellow, amorphous solid. The molecular formula was determined as C_21_H_19_O_7_ by HRESIMS from *m*/*z* 383.1125 [M + H] ^+^ (calcd for 383.1128, error: −0.6 ppm). In the ^1^H NMR spectrum were observed signals at δ 8.38 s (1H, s) and its corresponding olefinic carbon signal at δ 154.49 indicated the presence of an isoflavone skeleton.^33,34^ The ^1^H NMR spectrum also exhibited one singlet at δ 6.77 (1H, s) and two aromatic proton signals with *meta*-coupling at δ 6.73 (1H, d) and δ 6.92 (1H, d) with *J* = 2.0 Hz, indicating the presence of a penta- and tetrasubstituted ring in the structure. In the ^13^C NMR spectrum was observed a methine carbon signal at δ 90.82, which corroborates the presence of a penta-substituted ring due to the ortho-protective effect of the substituents on the ring.^35^ The ^1^H NMR spectrum also observed the presence of signals typical of a 2,2-dimethylpyran at δ 1.38 (6H, s) and two *cis*-coupled vinyl protons at δ 5.74 (1H, d, *J* = 9.8 Hz) and δ 6.37 (1H, d, *J* = 9.8 Hz) together with the signals at δ 27.50, δ 76.03, δ 131.29, and δ 122.08 in the ^13^C NMR spectrum.^36−42^ The presence of a singlet at δ 12.62 characteristic of a chelated hydroxyl in the ^1^H NMR spectrum indicates the insertion of a hydroxyl at position 5. The correlations between H-8 (δ 6.77) and carbons C-9 (δ 150.01), C-10 (δ 105.78), C-7 (δ 154.60), C-4 (δ 180.43), and C-5 (δ 146.53) present in the HMBC correlation spectrum confirmed the presence of a trioxygenation in ring A of the isoflavone. The insertion of the methoxyl was confirmed by the correlation of the protons at δ 3.89 of the methoxyl with C-7 (δ 154.60). The pyran portion was confirmed at positions C-4′ and C-5′ by the correlations of H-4″ (δ 6.37) with C-6′ (δ 140.5) and C-5′ (δ 110.3) (Figure S71). From these spectroscopic data, the structure of compound **1** was named diffusiflavone A.

The molecular formula of compound **2** was determined as C_22_H_21_O_7_ by HRESIMS at *m*/*z* 397.1282 [M + H] ^+^ (calcd for 397.1290, error: −2.0 ppm). The NMR spectroscopic data of compound **2** was very similar to that of compound **1**. The ^1^H NMR spectrum also exhibited typical signals of the isoflavone skeleton and 2,2-dimethylpyran in the structure. However, signals were observed at δ 3.87 in the ^1^H NMR spectrum and at δ 56.43 in the ^13^C NMR spectrum, which indicated that **2** was a methoxylated derivative of **1** whose insertion at the C-3′ position was confirmed by HMBC experiments through the correlations between the protons at δ 3.87 with C-3′ (δ 148.25). Analysis of the spectroscopic data led to the identification of compound **2**, which is named diffusiflavone B.

Compound **3** was shown to be an isomer of compound **2** with the molecular formula of C_22_H_21_O_7_ by HRESIMS of *m*/*z* 397.1282 [M + H]^+^ (calcd for 397.1290, error: −0.9 ppm). The NMR data were also very similar to compound **2**. However, a signal at δ 60.94 was observed in the ^13^C NMR spectrum which indicated the presence of a sterically hindered methoxyl^23^ in **3** suggesting insertion at the C-6 position, which was confirmed by HMBC experiments through the correlations between δ 3.89 and C-7 (δ 159.25). Based on these spectroscopic data, the structure of compound **3** has been determined and named diffusiflavone C.

Compound **4** was obtained as a white, amorphous solid. The molecular formula was determined as C_23_H_23_O_7_ by HRESIMS from *m*/*z* 411.1447 [M + H]^+^ (calcd for 411.1438, error: −2.2 ppm). As with compounds **2** and **3**, the ^1^H and ^13^C data were very similar, except for the presence of the signals at δ 3.93 (3H, s) and δ 3.90 (3H, s) in the ^1^H NMR spectrum and their corresponding carbons at δ 60.91 and δ 56.47, in addition to δ 3.88 and δ 56.40, which indicated that **4** was a polymethoxylated derivative of **1** whose insertion of methoxyls in the C-6 and C-3′ carbons was suggested by the maintenance of the chelated hydroxyl signal at δ 12.97 and confirmed by HMBC experiments. Spectroscopic data confirm the structure of compound **4**, designated as diffusiflavone D.

**Figure 3:**
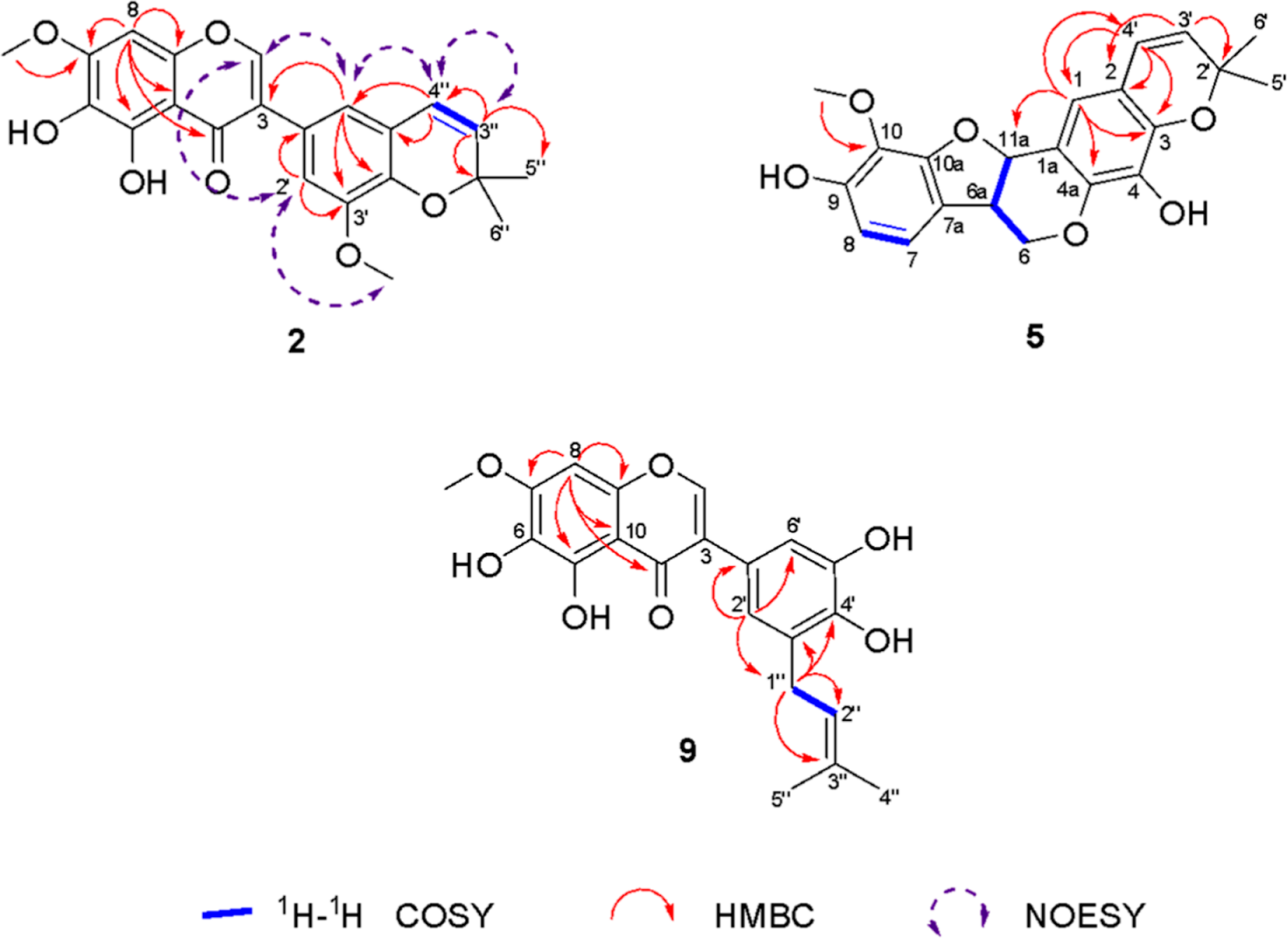
HMBC, ^1^H–^1^H COSY, and NOESY corrections of compounds **2**, **5**, and **9**.

Compound **9** was isolated as a white, amorphous solid. The molecular formula was C_21_H_21_O_7_ by HRESIMS at *m*/*z* 385.1282 [M + H]^+^ (calcd for 385.1286, error: −1.0 ppm). The NMR data observed the signals at δ 8.05 (1H, s) in the ^1^H NMR spectrum and δ 155.05 in the ^13^C NMR spectrum, one singlet at δ 6.68 (1H, s) and δ 91.51 in the ^13^C NMR spectrum, which indicated the presence of the same pattern observed in compounds **1** to **4** for ring A. The aromatic proton signals with *meta*-coupling were also observed at δ 6.73 (1H, d) and δ 6.90 (1H, d) with *J* = 2.1 Hz. However, in the ^1^H NMR spectrum were observed a triplet at δ 5.35 (1H, t, *J* = 7.5 Hz) and a duplet at δ 3.34 (1H, d, *J* = 7.5 Hz) and δ 1.74 (6H, s). The ^13^C NMR spectrum also were observed signals at δ 17.89, δ 25.94, and δ 29.36 which suggested the presence of a prenyl unit at **9**.^42,43^ In the HMBC spectrum of **9** (Figure 3), correlations were observed between H-1″ and C-3′ (δ 129.76), C-4′ (δ 144.61), and C2″ (δ 123.96) and between H-2′ and C-1′(δ 124.70), C1″ (δ 29.36), and C-6′ (δ 114.81) which confirmed the insertion of the prenyl into C-3′. The spectroscopic data described allowed **9** to be defined as diffusiflavone E.^44,45^

Compound **5** was isolated as a yellowish, amorphous solid. The molecular formula was determined as C_22_H_22_O_6_ by HRESIMS at *m*/*z* 383.1125 [M + H]^+^ (calcd for 383.1128, error: −2.6 ppm). In the ^1^H NMR spectrum were observed signals at δ 3.56 (1H, m), δ 3.66 (1H, m), δ 4.32 (1H, ddd, *J* = 1.0, 4.8, 10.6 Hz), and δ 5.51 (1H, d, *J* = 6.6 Hz), a classic hydrogenation pattern for the presence of two asymmetric centers characteristic of position C-6, C-6a, and C-11 of a pterocarpan skeleton.^46−48^ In addition, the couplings of the aliphatic hydrogens H-6a and H-11a with *J* = 6.60 Hz and the chemical shifts of C-6, C-6a, and C-11a at δ 66.75, δ 40.20, and δ 79.18, respectively, together indicate the presence of a *cis* configuration in compound **5**. In the ^1^H NMR spectrum also was observed the presence of typical 2,2-dimethylpyran signals at δ 1.47 (3H, s) and δ 1.43 (3H, s), two *cis*-coupled hydrogens at δ 5.54 (1H, d, *J* = 9.8 Hz) and δ 6.61 (1H, d, *J* = 9.8 Hz), two ortho-coupled signals, δ 6.73 (1H, dd, *J* = 1.0 and 8.1 Hz) and δ 6.44 (1H, d, *J* = 8.1 Hz), and the singlet at δ 6.84 (1H, s) suggested the presence of tetra- and penta-substituted ring in the structure.^46,47^ The pyran portion was confirmed in positions C-2 and C-3 due to the “cross-peak” correlations observed between H-4′ and C-1/C-2/C-3 and between H-3′ and C-2 and the insertion of the methoxyl into C-10 was confirmed by the correlations between δ 3.84 and C-10 (δ 130.65). From these spectroscopic data, the structure of compound **5** was named diffusicarpan A.

Compound **6** was isolated as a yellowish amorphous solid with a molecular formula of C_21_H_21_O_7_ determined by HRESIMS at *m*/*z* 385.1282 [M + H]^+^ (calcd for 385.1286, error: −0.6 ppm). The NMR data indicate a similar structure to compound **5**. However, the signals at δ 3.86 and δ 61.06 demonstrated the insertion of a sterically hindered methoxyl into the structure where the insertion at the C-9 position was confirmed by HMBC spectra through the correlations between the δ 3.86 signals and C-9 (δ 148.06). From these spectroscopic data, compound **6** was structurally elucidated and designated as diffusicarpan B.

The effects of the compounds on cell viability were assessed using the MTT assay to ensure the noncytotoxic concentration ranges. The substances tested did not produce cell death in RAW 264.7 macrophages, when compared to the control group, at concentrations of 1.25, 2.5, 5, 10, and 20 μg/mL (Figure S72).

**Figure 4:**
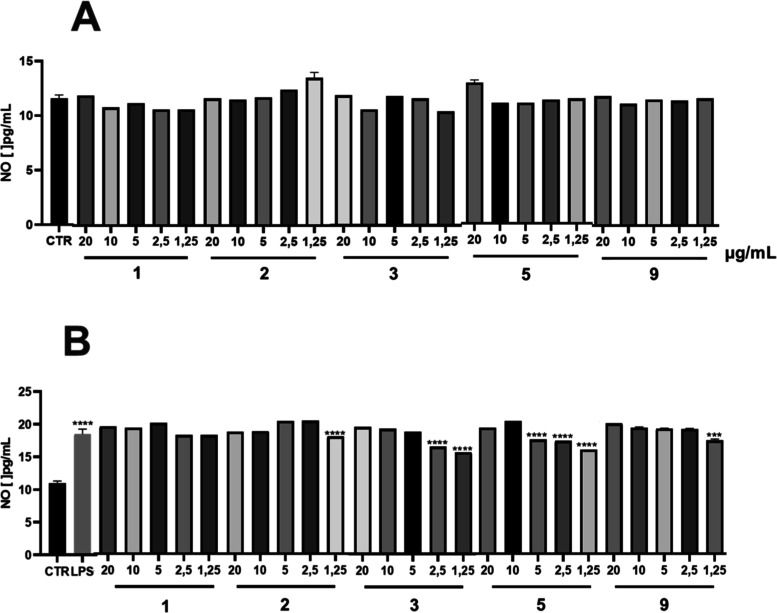
Indirect nitric oxide assay by the Griess reagent method. (A) RAW 264.7 macrophages without LPS exposure were exposed to the compounds at different concentrations of 1.25, 2.5, 5, 10, and 20 μg/mL. CTR: control group. (B) LPS-stimulated RAW 264.7 macrophages were exposed to the compounds at different concentrations of 1.25, 2.5, 5, 10, and 20 μg/mL (the results are presented as mean ± standard error of the mean where the values for *p < 0.05, **p < 0.01, ***p < 0.001, and ****p < 0.0001 when compared to the LPS group were considered significant. The data were analyzed using one-way ANOVA followed by the Bonferroni post-test for comparisons between established groups).

The compounds tested did not induce NO in RAW 264.7 macrophages without exposure to LPS (Figure 4A). Additionally, compounds **1**, **4**, **5**, and **6** reduced NO levels in macrophage cultures stimulated with LPS compared to cells stimulated and treated with vehicle (*p* < 0.001), at concentrations of 1.25 to 5 μg/mL (Figure 4B). These data indicate that the compounds have anti-inflammatory activity.

**Figure 5:**
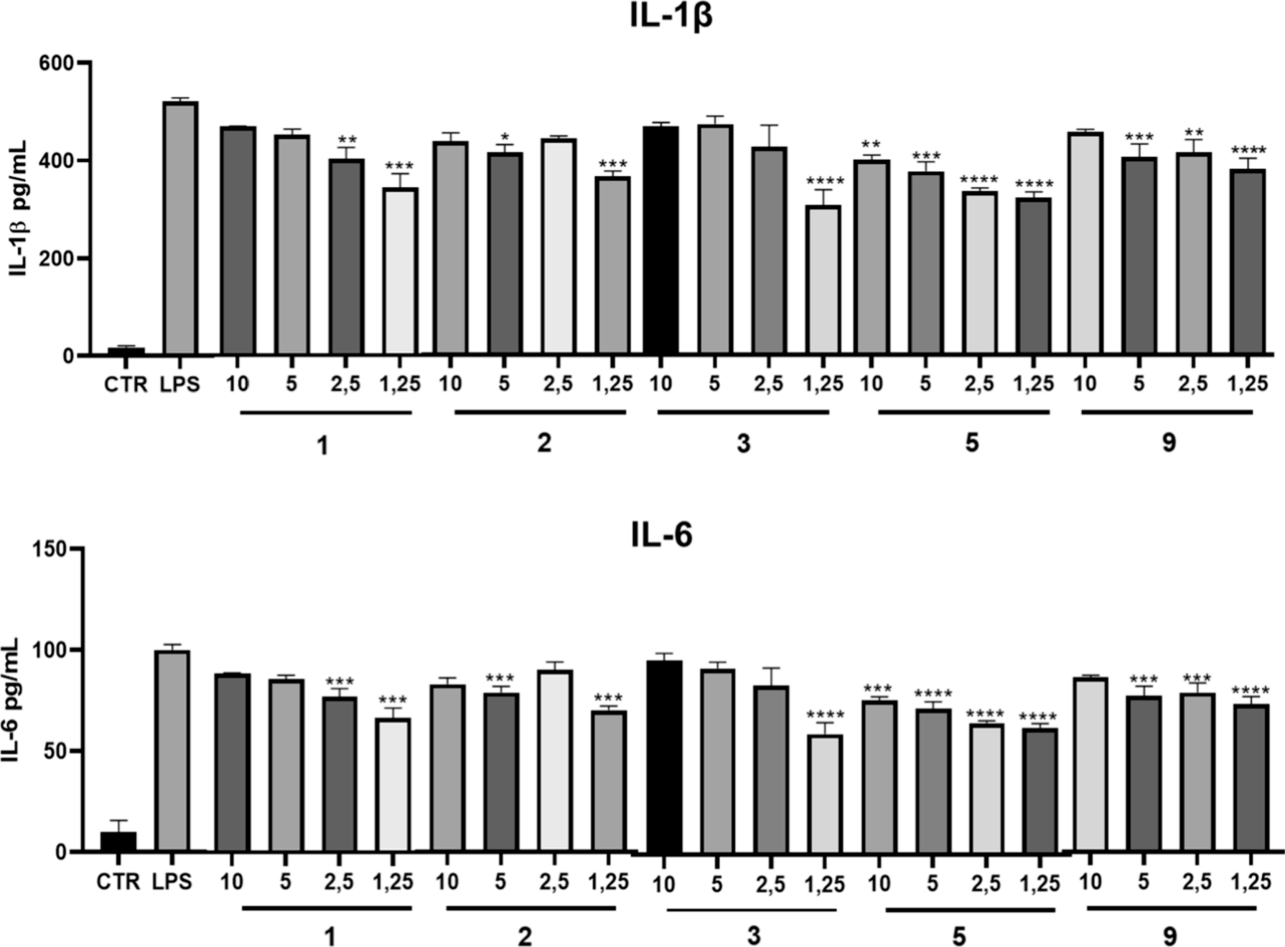
Levels of IL-1β and IL-6 in LPS-stimulated RAW 264.7 macrophages exposed to the compounds at different concentrations 1.25, 2.5, 5, 10, and 20 μg/mL. CTR: control group (the results are presented as mean ± standard error of the mean where the values for *p < 0.05, **p < 0.01, ***p < 0.001, and ****p < 0.0001 when compared to the LPS group were considered significant. The data were analyzed using one-way ANOVA followed by the Bonferroni post-test for comparisons between established groups).

Consistent with these results, ELISA data (Figure 5) show that compounds **1** (1.25 and 2.5 μg/mL), **2** and **9** (1.25, 2.5, and 5 μg/mL), **3** (1.25 μg/mL), and **5** (all concentrations tested) reduced IL-1β and IL-6 production in macrophages stimulated with LPS in a dose-independent manner (*p* < 0.01). The maximum inhibitory effects on NO, IL-6, and IL-1β production induced by **5** were 14.0%, 38.0%, and 38.2%, respectively. These results indicate that the compounds can inhibit macrophage responses to inflammatory stimuli. Considering the fundamental role of macrophages and their mediators in inflammatory processes,^49−52^ the data reinforce the anti-inflammatory potential of the compounds.

This work demonstrates the usefulness of molecular networking as a dereplication strategy for isolating novel bioactive compounds. The anti-inflammatory properties observed for the isolated prenylated isoflavonoids and pterocarpans emphasize the therapeutic potential of *A. diffusissimum*. Further investigations should focus on exploring the promising chemical and pharmacological properties of this species.

## Experimental Section

### General Experimental Procedures

The IR spectra were recorded on a Shimadzu FTIR Spirit spectrometer. The 1D and 2D nuclear magnetic resonance (NMR) spectra were obtained on Bruker AVANCE III Hb spectrometers at 400 and 500 MHz (Bruker Co., Ltd., Bremen, Germany). The residual nondeuterated solvent peaks were used as references. High-resolution mass spectrometric data analyses were recorded using a microTOFII ESI-TOF mass spectrometer, and tandem mass spectrometry (MSn) was performed on an AmazonX ESI-IT instrument (Bruker, Billerica, MA, USA). Preparative C18 (Shim-pack GIST, 250 mm × 20 mm, 5 μm) and analytical C18 columns (Shim-pack GIST, 250 mm × 4.6 mm, 5 μm) were used for the separation. Silica gel with particles of 40–63 μm, 230–400 mesh, and 60 Å was used for column chromatography (CC) and vacuum liquid chromatography (VLC).

### Plant Material

The stems of *A. diffusissimum* were collected in January 2021 at Fazenda Esperança (14°43′28″S and 40°42′37″W) located in the municipality of Boa Vista do Tupim, Bahia, Brazil, and identified by Domingos Bencio Oliveira Silva Cardoso. An exsiccate is deposited in the Herbarium of the State University of Feira de Santana (HUEFS), in Bahia, under number 246074. The species was registered for access to the genetic heritage in SisGen with process no. A39AEA5.

### Analysis by LC-ESIMS^n^

The rich fraction of flavonoids was analyzed by HPLC (Shimadzu), using an analytical chromatographic C18 column (Kromasil; 250 × 4.6 × 5 μm) coupled to a mass spectrometer (Ion-Trap AmazonX, Bruker), with electrospray ionization (ESI). The analyzed samples were solubilized in MeOH (1 mg/mL) and filtered with PVDF filters (0.45 μm). The separation process was performed through a gradient system by using acidified water (0.1% HCOOH) as solvent A and MeOH as solvent B, with an injection volume of 10 μL and a flow rate of 0.6 mL/min. The ESI source parameters were defined as follows: capillary, 4.5 kV; end plate offset, 500 V; nebulizer, 35 psi; dry gas, N2; flow rate of 8 mL/min; and temperature of 300 °C. The analyses were performed by operating in the positive mode with a range of *m*/*z* 50–1500.

### Molecular Networking

The data obtained by LC–MS/MS were converted into mzXML format directly from Bruker Data Analysis 4.2. The molecular networks were generated using GNPS (http://gnps.ucsd.edu) with the following parameters: parent mass tolerance of 2.0 Da, MS/MS fragment ion tolerance of 0.5 Da, cosine score of 0.7 and minimum matching peaks of 6, analogue search, and filter spectra of G6 as blanks before the network was enabled. The data were subsequently visualized using Cytoscape 3.10.2 software. The dereplicated compounds were identified based on the comparison of MS/MS data with the literature.

### Extraction and Isolation

The dried and powdered plant material was obtained from 5.7 kg of stems, which was extracted at room temperature with 96% EtOH (×3 for 72 h) with later concentration through a rotary evaporator at 40 °C to obtain 220 g of crude extract (CE). One portion of CE (150 g) was subjected to defatting with hexane (3 × 500 mL), yielding 130 g of crude defatted ethanolic extract which was subjected to vacuum liquid chromatography using silica gel and eluted with *n*-hexane, *n*-hexane/CHCl_3_, CHCl_3_, EtOAc, EtOAc/MeOH, and MeOH. The CHCl_3_ phase (1.4 g) was submitted to column chromatography on silica gel, using an elution gradient of hexane/EtOAc (95:5, 90:10, 85:15, 80:20, 75:25, 1:1, 25:75, and 0:100), EtOAc/MeOH (1:1), and MeOH. Fractions of 125 mL were collected, and 14 fractions were obtained. Fractions 8 and 9 (68 mg) were grouped and subjected to preparative RP-HPLC using MeOH and H_2_O with 0.1% HCOOH as the eluent (8 mL/min) through a gradient system with the following parameters: 0–15 min (40–70% B) and 15–60 min (70% B), obtaining compounds **4** (1.5 mg) and **6** (1.0 mg). Fractions 10–13 (328 mg) were grouped and subjected to preparative RP-HPLC using MeOH and H2O with 0.1% HCOOH as the eluent (8 mL/min) through a gradient system with the following parameters: 0–60 min (65% B), obtaining compounds **1** (11.5 mg), **2** (6.0 mg), **3** (6.9 mg), and **5** (12.8 mg). The AcOEt phase (4.2 g) was submitted to column chromatography on silica gel, using an elution gradient of hexane/CH_2_Cl_2_ (1:1 and 20:80), CH_2_Cl_2_, CH_2_Cl/_2_EtOAc (80:20, 1:1, and 20:80), EtOAc, EtOAc/MeOH (80:20 and 1:1), and MeOH. Fractions of 125 mL were collected, and 13 fractions were obtained. Fractions 6 and 7 (1.35 g) were fractionated to column chromatography on silica gel, using an elution gradient of CHCl_3_/EtOAc (85:15, 80:20, and 1:1), EtOAc, EtOAc/MeOH (1:1), and MeOH. Fractions of 50 mL were collected, and 13 fractions were obtained. Fraction 67–2 (78 mg) was subjected to preparative RP-HPLC using MeOH and H_2_O with 0.1% HCOOH as the eluent (8 mL/min) through a gradient system with the following parameters: 0–15 min (40–70% B), 15–20 min (70–80% B), and 20–60 min (80% B), obtaining compound **7** (2 mg) and compound **9** (11.7 mg). Fraction 67–4 (302 mg) was subjected to preparative RP-HPLC using MeOH and H_2_O with 0.1% HCOOH as the eluent (8 mL/min) through a gradient system with the following parameters: 0–60 min (42–70% B), obtaining compound **8** (7 mg).

Diffusiflavone A (**1**): white amorphous solid; IR (liquid solution) ν_max_ 3371, 2920, 2849, 1657, 1576, 1502, 1306, 1206, 1273, 1131, 1051, 1022, 726, and 450 cm^–1^; ^1^H and ^13^C NMR data, see Table 1; positive-ion HRESIMS *m*/*z* 383.1125 [M + H] ^+^ (calcd for C_21_H_19_O_7_, 383.1128, Δ = −0.6 ppm).

**Table 1:** ^1^H and ^13^C NMR Spectroscopic Data of Compounds **1–4** and **9**

	**1**^a^		**2**^c^		**3**^c^		**4**^c^		**9**^b^	
position	δ_C_	δ_H,multi.(*J*,HZ)_	δ_C_	δ_H,multi.(*J*,HZ)_	δ_C_	δ_H,multi.(*J*,HZ)_	δ_C_	δ_H,multi.(*J*,HZ)_	δ_C_	δ_H,multi.(*J*,HZ)_
2	154.49	8.38 s	153.01	7.89 s	152.82	7.86 s	153.36	7.88 s	155.05	8.05 s
3	121.48		123.28		123.17		123.21		123.06	
4	180.43		180.88		180.96		180.70		182.61	
5	146.53		145.99		153.49		152.57		147.61	
6	130.03		129.60		133.40		132.68		131.61	
7	154.60		152.93		158.91		159.13		155.75	
8	90.82	6.77 s	90.20	6.49 s	90.33	6.43 s	90.52	6.44 s	91.51	6.68 s
9	150.01		150.86		153.56		153.05		152.27	
10	105.78		106.53		106.84		106.80		107.47	
1′	121.48		122.68		123.41		123.25		124.70	
2′	117.63	6.73 d (2.0)	113.28	6.76 d (2.0)	115.39	6.76 d (2.0)	113.23	6.76 d (2.0)	122.20	6.73 d (2.1)
3′	145.14		148.25		144.51		158.72		129.76	
4′	140.04		142.40		139.61		132.47		144.61	
5′	122.94		121.96		121.21		148.00		145.84	
6′	117.06	6.92 d (2.0)	119.26	6.97 d (2.0)	118.59	6.96 d (2.0)	117.20	6.96 d (2.0)	114.81	6.90 d (2.1)
1″									29.36	3.34 d (7.5)
2″	76.03		76.78		77.68		76.54		123.96	5.35 t (7.5)
3″	131.29	5.74 d (9.8)	131.16	5.64 d (9.4)	130.98	5.64 d (10.0)	131.20	6.30 d (10.0)	133.04	
4″	122.04	6.37 d (9.8)	122.09	6.32 d (9.4)	122.03	6.35 d (10.0)	122.05	5.63 d (10.0)	17.89	1.74 s
5″	27.50	1.38 s	27.94	1.47 s	28.10	1.46 s	27.55	1.48 s	25.94	1.74 s
6″	27.50	1.38 s	27.94	1.47 s	28.10	1.46 s	27.55	1.48 s		
5-OH		12.62 s		12.62 s		12.80 s		12.97 s		
6-OH		8.74 sl							8.05	
6-OCH_3_					60.94	3.89 s	60.91	3.93 s		
7-OCH_3_	56.37	3.89 s	56.45	3.96 s	56.30	3.93 s	56.40	3.88 s	56.90	3.97 s
3′-OCH_3_			56.43	3.87 s			56.47	3.90 s		
3′-OH		8.97 sl								

a DMSO-*d*6 (500 MHz/125 MHz).

b MeOD (500 MHz/125 MHz).

c CDCl_3_ (400 MHz/100 MHz).

Diffusiflavone B (**2**): yellow amorphous solid; IR (liquid solution) ν_max_ 3397, 2972, 2918, 2849, 1662, 1576, 1459, 1361, 1295, 1273, 1246, 1206, 1183, 1168, 1134, and 1088 cm^–1^; ^1^H and ^13^C NMR data, see Table 1; positive-ion HRESIMS *m*/*z* 397.1282 [M + H] ^+^ (calcd for C_22_H_21_O_7_, 397.1290, Δ = −2.0 ppm).

Diffusiflavone C (**3**): yellow amorphous solid; IR (liquid solution) ν_max_ 3426, 2926, 2852, 1654, 1617, 1585, 1493, 1459, 1364, 1301, 1255, 1206, 1183, 1134, 1085, 1062, 1028, 988, 855, 818, and 726 cm^–1^; ^1^H and ^13^C NMR data, see Table 1; positive-ion HRESIMS *m*/*z* 397.1282 [M + H] ^+^ (calcd for C_22_H_21_O_7_, 397.1285, Δ = −0.9 ppm).

Diffusiflavone D (**4**): white amorphous solid; IR (liquid solution) ν_max_ 2918, 1651, 1625, 1493, 1459, 1427, 1312, 1289, 1209, 1137, and 789 cm^–1^; ^1^H and ^13^C NMR data, see Table 1; positive-ion HRESIMS *m*/*z* 411.1447 [M + H]^+^ (calcd for C_23_H_23_O_7_, 411.1438, Δ = −0.9 ppm).

Diffusicarpan A (**5**): yellow amorphous solid; IR (liquid solution) ν_max_ 3437, 2972, 2932, 1628, 1499, 1476, 1404, 1364, 1275, 1246, 1209, 1168, 1134, 1079, 1054, 1031, 939, 855, and 752 cm^–1^; ^1^H and ^13^C NMR data, see Table 2; positive-ion HRESIMS *m*/*z* 369.1333 [M + H]^+^ (calcd for C_21_H_21_O_6_, 369.1342, Δ = −2.6 ppm).

**Table 2:** ^1^H and ^13^C NMR Spectroscopic Data of Compounds **5** and **6** (CDCl_3_, 400 MHz)

	5		6	
position	δ_C_	δ_H,multi.(*J*,HZ)_	δ_C_	δ_H,multi.(*J*,HZ)_
1	118.83	6.84 s	122.88	6.99 s
1a	112.57		112.69	
2	115.97		116.67	
3	140.27		149.59	
4	132.91		136.74	
4a	143.66		149.47	
5				
6	66.75	3.66–3.58 m 4.32 ddd (10.8, 5.0, 1.0)	66.63	3.65 t (11) 4.33 dd (5.0, 11)
6a	40.20	3.56–3.51 m	40.02	3.55 m
7	114.79	6.73 dd (8.1, 1.0)	114.83	6.73 d (8,0)
7a	121.35		121.35	
8	103.74	6.44 d (8.1)	103.72	6.44 d (8,0)
9	148.05		148.06	
10	130.65		136.71	
10a	146.05		149.21	
11				
11a	79.18	5.51 d (6.6)	79.34	5.53 d (6.5)
1′				
2′	77.20		76.75	
3′	129.26	5.54 d (9.8)	129.23	5.54 d (9.5)
4′	121.85	6.28 d (9.8)	121.89	6.28 d (9.5)
5′	28.13	1.47 s	28.11	1.46 s
6′	27.89	1.43 s	27.81	1.42 s
4-OH		5.31 sl		
9-OCH_3_			61.06	3.86 s
9-OH				
10-OCH_3_	56.45	3.84 s	56.45	3.85 s

Diffusicarpan B (**6**): yellow amorphous solid; ^1^H and ^13^C NMR data, see Table 2; positive-ion HRESIMS m/z 383.1125 [M + H]^+^ (calcd for C_22_H_23_O_6_, 383.1128, Δ = −0.6 ppm).

Diffusiflavone E (**9**): white amorphous solid; IR (liquid solution) ν_max_ 3426, 3406, 2923, 2854, 1662, 1614, 1576, 1499, 1459, 1441, 1367, 1301, 1209, 1131, 1082, and 1056 cm^–1^; ^1^H and ^13^C NMR data, see Table 1; positive-ion HRESIMS *m*/*z* 385.1282 [M + H]^+^ (calcd for C_21_H_21_O_7_, 385.1286, Δ = −1.0 ppm).

### Cell Culture of RAW 264.7

RAW 264.7 cell line (ATCC No. TIB71) was used in the study, which is monocytes/macrophages derived from a tumor induced in the *Mus musculus* species. For in vitro stimulation assays, they were cultured in DMEM (VITROGEN) supplemented with 10% fetal bovine serum and 1% antibiotic (streptomycin and penicillin) and were maintained in cell culture flasks in an incubator at 37 °C with a 5% CO_2_ atmosphere.

### Cell Viability

Cell viability was estimated by an MTT assay (Mosmann 1983). For that, 100 μL of complete RPMI-1640 medium containing 10 μL of MTT solution (MTT 5 mg/mL in PBS) was added to each well. After 4 h of incubation, the MTT-containing medium was discarded, and the precipitate was solubilized in 100 μL of DMSO. Optical density was read at 570 nm using a microplate spectrophotometer BioTek ELx800, Thermo Fisher Scientific Inc. (Waltham, MA, US).

### Nitric Oxide and Cytokine Production

Nitric oxide production was indirectly estimated by measurement of nitrite, a major stable product of nitric oxide, using Griess solution (Griess, 1879). Briefly, 50 μL of the culture supernatant was mixed with an equal amount of Griess solution (0.1% *N*-[1-naphthyl] ethylenediamine and 1% sulfanilamide in 5% *ortho*-phosphoric acid) at room temperature for 10 min. Optical density was read at 540 nm using a BioTek ELx800 microplate spectrophotometer, Thermo Fisher Scientific Inc. (Waltham, MA, US). Sodium nitrite was used for the standard curve.

IL-1β and IL-6 levels in the culture supernatant were determined by sandwich ELISA, according to the manufacturer’s instructions. Optical density was read at 450 nm using a microplate spectrophotometer from Thermo Fisher Scientific Inc. (Waltham, MA, US).

### Statistical Analysis

Inhibition curves were fitted by nonlinear regression analysis assuming a sigmoidal concentration–response curve model and the half-maximal inhibitory concentration was estimated. All data were expressed as mean ± standard error of the mean (SEM) and analyzed using GraphPad Prism software version 9.1.0, GraphPad Software Inc. (San Diego, CA, US), using one-way analysis of variance followed by Tukey’s test for multiple comparisons. The results were considered statistically significant when *p* < 0.05.

## Supporting Information Available

Nuclear magnetic resonance and infrared spectra; fragmentation patterns obtained by mass spectrometry, which aid in the structural characterization of the investigated compounds; and data from in vitro pharmacological activity assays, specifically regarding the cell viability of the RAW 264.7 macrophage lineage (PDF)
