# Supplementary material for: Target Isolation of Prenylated Isoflavonoids and Pterocarpans from Acosmium diffusissimum Using LC–MS/MS-Based Molecular Networking
Source: ACS Omega. 2025 Mar 27;10(13):13645–54. doi: 10.1021/acsomega.5c00866 (PMC11983342; doi:10.1021/acsomega.5c00866)

Supplementary Information

Target isolation of prenylated isoflavonoids and pterocarpans from *Acosmium diffusissimum* using LC-MS/MS based Molecular Networking

Gabriela Ribeiro de Sousa[1], Natanael Ramos de Lima Teles[1], Carlos Vinicius Azevedo da Silva[2], Mariana Costa Aragão[1], Domingos Benício Oliveira Silva Cardoso[3], Francisco Allysson Assis Ferreira Gadelha[4], Marcia Regina Piuvezam[4], Josean Fechine Tavares[1], Marcelo Sobral da Silva[1], José Maria Barbosa Filho[1*]

[1]Laboratório Multiusuário de Caracterização e Análises, Programa de Pós-Graduação em Produtos Naturais e Sintéticos Bioativos, Centro de Ciências da Saúde, Universidade Federal da Paraíba, João Pessoa 58051-900, Paraíba, Brazil.

[2]Grupo de Pesquisa em Metabolômica e Espectrometria de Massas, Universidade Estadual do Amazonas (UEA), 690065-130 Manaus-AM, Brazil

[3]Instituto de Biologia, Universidade Federal da Bahia, Salvador 40170-115, Bahia, Brazil.

[4]Laboratório de Imunofarmacologia, Programa de Pós-Graduação em Produtos Naturais e Sintéticos Bioativos, Centro de Ciências da Saúde, Universidade Federal da Paraíba, João Pessoa 58051-900, Paraíba, Brazil.

CONTENT

Figure S1. Base peak chromatograms of the fraction of flavonoids of *A. diffusissimum* stems in positive mode and peaks of the isolated substances indicated by their respective numbers 6

Table S1: LC-ESI/MSn and LC-HRESIMS data of compounds of cluster A 7

Scheme S1. Fragmentation proposal of compound 1 (m/z 383, rt: 41.1 min, cluster A) 8

Figure S2. HRESIMS and LC-ESIMS/MS spectrum of compound 1 ([M + H] $^{+}$, positive mode) 9

Figure S3. ^{1}H NMR spectrum of 1 at 500 MHz in DMSO-*d6* 10

Figure S4. ^{1}H NMR spectrum of 1 at 500 MHz in DMSO-*d6* (expansion: 5.5 – 9.3 ppm) 10

Figure S5. ^{13}C NMR spectrum of 1 at 125 MHz in DMSO-*d6* 11

Figure S6. ^{13}C NMR spectrum of 1 at 125 MHz in DMSO-*d6* (expansion: 115 – 155 ppm) 11

Figure S7. ^{13}C NMR DEPT135 spectrum of 1 at 125 MHz in DMSO-*d6* 12

Figure S8. HSQC spectrum of 1 at 500 MHz in DMSO-*d6* 12

Figure S9. HMBC spectrum of 1 at 500 MHz in DMSO-*d6* 13

Figure S10. COSY spectrum of 1 at 500 MHz in DMSO-*d6* 13

Figure S11. IR spectrum of 1 (liquid solution) 14

Scheme S2. Fragmentation proposal of compound 2 (m/z 397, rt: 44.9 min, cluster A) 15

Figure S12. HRESIMS and LC-ESIMS/MS spectrum of 2 ([M + H] $^{+}$, positive mode) 16

Figure S13. ^{1}H NMR spectrum of 2 at 400 MHz in $CDCl_3$ 17

Figure S14. ^{1}H NMR spectrum of 2 at 400 MHz in $CDCl_3$ (expansion: 3.6 – 8.8 ppm) 17

Figure S15. ^{13}C NMR spectrum of 2 at 100 MHz in $CDCl_3$ 18

Figure S16. ^{13}C NMR spectrum of 2 at 100 MHz in $CDCl_3$ (expansion: 106 - 156 ppm) 18

Figure S17. DEPT-135 spectrum of 2 at 100 MHz in $CDCl_3$ 19

Figure S18. HSQC spectrum of 2 at 400 MHz in $CDCl_3$ 19

Figure S19. HMBC spectrum of 2 at 400 MHz in $CDCl_3$ 20

Figure S20. COSY spectrum of 2 at 400 MHz in $CDCl_3$ 20

Figure S21. NOESY spectrum of 2 at 400 MHz in $CDCl_3$ 21

Figure S22. IR spectrum of 2 (liquid solution) 21

Scheme S3. Fragmentation proposal of compound 3 (*m/z* 397, rt: 47.4 min, cluster A)....................22
Figure S23. HRESIMS and LC-ESIMS/MS spectrum of compound 3 ([M + H] $^+$, positive mode)....................23
Figure S24. ^{1}H NMR spectrum of 3 at 400 MHz in $CDCl_3$....................24
Figure S25. ^{1}H NMR spectrum of 3 at 400 MHz in $CDCl_3$ (4.0 – 8.5 ppm)....................24
Figure S26. ^{13}C NMR spectrum of 3 at 400 MHz in $CDCl_3$....................25
Figure S27. ^{13}C NMR spectrum of 3 at 400 MHz in $CDCl_3$ (expansion: 102 - 164 ppm)....................25
Figure S28. HSQC spectrum of 3 at 400 MHz in $CDCl_3$....................26
Figure S29. HMBC spectrum of 3 at 400 MHz in $CDCl_3$....................26
Figure S30. NOESY spectrum of 3 at 400 MHz in $CDCl_3$....................27
Figure S31. COSY spectrum of 3 at 400 MHz in $CDCl_3$....................27
Figure S32. IR spectrum of 3 (liquid solution)....................28
Scheme S4. Fragmentation proposal of compound 4 (m/z 411, rt: 51.8 min, cluster A)....................29
Figure S33. HRESIMS and LC-ESIMS/MS spectrum of compound 4 ([M + H] $^+$, positive mode)....................30
Figure S34. ^{1}H NMR spectrum of 4 at 500 MHz in $CDCl_3$....................31
Figure S35. ^{1}H NMR spectrum of 4 at 500 MHz in $CDCl_3$ (expansion: 3.2 – 8.2 ppm)....................31
Figure S36. ^{13}C NMR spectrum of 4 at 500 MHz in $CDCl_3$....................32
Figure S37. HSQC spectrum of 4 at 500 MHz in $CDCl_3$....................32
Figure S38. HMBC spectrum of 4 at 500 MHz in $CDCl_3$....................33
Figure S39. HMBC spectrum of 4 at 500 MHz in $CDCl_3$ (expansion:)....................33
Figure S40. IR spectrum of 4 (liquid solution)....................34
Scheme S5. Fragmentation proposal of compound 5 (m/z 369, rt: 36.4 min, cluster D)....................35
Figure S41. HRESIMS and LC-ESIMS/MS spectrum of 5 ([M + H] $^+$, positive mode)....................36
Figure S42. ^{1}H NMR spectrum of 5 at 400 MHz in $CDCl_3$....................37
Figure S43. ^{1}H NMR spectrum of 5 at 400 MHz in $CDCl_3$ (expansion: 3.3 – 4.4 ppm)....................37
Figure S44. ^{1}H NMR spectrum of 5 at 400 MHz in $CDCl_3$ (expansion: 5.2 – 7.3 ppm)....................38
Figure S45. ^{13}C NMR spectrum of 5 at 500 MHz in $CDCl_3$....................38
Figure S46. ^{13}C NMR spectrum of 5 at 500 MHz in $CDCl_3$ (expansion: 100 – 150 ppm)....................39

Figure S47. DEPT135 spectrum of 5 at 500 MHz in $CDCl_3$ 39

Figure S48. HSQC spectrum of 5 at 500 MHz in $CDCl_3$ 40

Figure S49. HMBC spectrum of 5 at 500 MHz in $CDCl_3$ 40

Figure S50. COSY spectrum of 5 at 500 MHz in $CDCl_3$ 41

Figure S51. IR spectrum of 5 (liquid solution) 41

Scheme S6. Fragmentation proposal of compound 6 (m/z 383, rt: 42.7 min, cluster D) 42

Figure S52. HRESIMS and LC-ESIMS/MS spectrum of 6 ($[M + H]^+$, positive mode) 43

Figure S53. ^1H NMR spectrum of 6 at 400 MHz in $CDCl_3$ 44

Figure S54. ^1H NMR spectrum of 6 at 400 MHz in $CDCl_3$ (expansion: 3.4 – 4.4 ppm) 44

Figure S55. ^1H NMR spectrum of 6 at 400 MHz in $CDCl_3$ (expansion: 5.0 – 7.3 ppm) 45

Figure S56. ^{13}C NMR spectrum of 6 at 100 MHz in $CDCl_3$ 45

Figure S57. HSQC spectrum of 6 at 400 MHz in $CDCl_3$ 46

Figure S58. HMBC spectrum of 6 at 400 MHz in $CDCl_3$ 46

Figure S59. COSY spectrum of 6 at 400 MHz in $CDCl_3$ 47

Figure S60. NOESY spectrum of 6 at 400 MHz in $CDCl_3$ 47

Table S2: LC-ESIMS[3] and LC-HRESIMS data of compounds 48

Figure S61. HRESIMS spectrum of 9 ($[M + H]^+$, positive mode) 50

Figure S62. ^1H NMR spectrum of 9 at 500 MHz in MeOD 51

Figure S63. ^1H NMR spectrum of 9 at 500 MHz in MeOD (expansion: 5.1 – 8.7 ppm) 51

Figure S64. ^{13}C NMR spectrum of 9 at 125 MHz in MeOD 52

Figure S65. ^{13}C NMR spectrum of 9 at 125 MHz in MeOD (expansion: 90 – 160 ppm) 52

Figure S66. DEPT135 spectrum of 9 at 125 MHz in MeOD 53

Figure S67. HSQC spectrum of 9 at 500 MHz in MeOD 53

Figure S68. HMBC spectrum of 9 at 500 MHz in MeOD 54

Figure S69. COSY spectrum of 9 at 500 MHz in MeOD 54

Figure S70. IR spectrum of 9 (liquid solution) 55

Figure S71. HMBC, ^1H-^1H COSY and NOESY corrections of compounds from stems of *Acosmium diffusissimum* 56

Figure S72. Cell viability of RAW 264.7 macrophages assessed using the MTT method. RAW 264.7 macrophages were exposed to the compounds at different concentrations of 1.25, 2.5, 5, 10 and 20 µg/mL. The results are presented as mean ± standard error of the mean where values for *p<0.05, **p<0.01, ***p<0.001 and

****p<0.0001 when compared to the control group (CTR) were considered significant. The data was analyzed using one-way ANOVA followed by the Bonferroni post-test for comparisons between established groups. 57

Figure S1. Base peak chromatograms of the fraction of flavonoids of *A. diffusissimum* stems in positive mode and peaks of the isolated substances indicated by their respective numbers

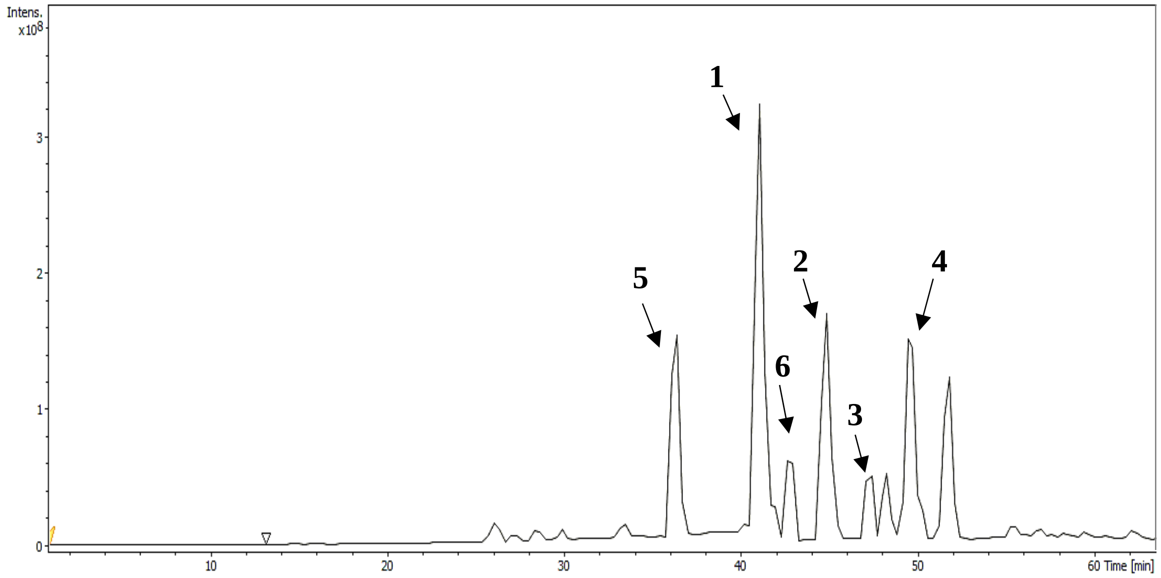

Table S1: LC-ESI/MSn and LC-HRESIMS data of compounds of cluster A

Rt (min)	*Meas.* m/z	m/z $[M+H]^+$	Formula	Error (ppm)	MS^n *m/z*	Compound	Cluster
33.2	415.1407	415	$C_{22}H_{23}O_8$	-4.8	MS^2 [415]: 397 (100), 379 (10), 355 (24), 343 (47), 327 (8.8), 241 (19), 183 (80); MS^3 [415 → 397]: 355 (100), 327 (6.9), 241 (4.7), 183 (20)	-	A
41.1	383.1128	383	$C_{21}H_{19}O_7$	- 0.6	MS^2 [383]: 365 (53), 337 (20), 227 (18), 183 (100); MS^3 [383 → 183]: 151 (35)	1	A
44.9	397.1290	397	$C_{22}H_{21}O_7$	- 2.0	MS^2 [397]: 382 (100), 365 (36), 337 (21), 241 (9.6), 183 (49); MS^3 [397 → 382]: 364 (100), 346 (37), 227 (7.1), 200 (96), 183 (62), 155 (4.2), 136 (5.2)	2	A
47.1	397.1285	397	$C_{22}H_{21}O_7$	- 0.9	MS^2 [397]: 379 (40), 351 (22), 227 (14), 197 (100), 183 (8.1); MS^3 [397 → 197]: 182 (100), 163 (54), 151 (21), 139 (12)	3	A
49.8	367.1194	367	$C_{21}H_{19}O_6$	-4.9	MS^2 [367]: 349 (31), 325 (14), 211 (17), 183 (100); MS^3 [367 → 183]: 167 (8.6), 165 (65), 155 (24)	-	A
51.6	411.1447	411	$C_{23}H_{23}O_7$	- 2.2	MS^2 [411]: 396 (100), 379 (36), 351 (13), 197 (50); MS^3 [411 → 396]: 378 (100), 363 (34), 332 (2.1), 227 (4.2), 200 (27), 179 (7.2)	4	A
56.8	381.1338	381	$C_{22}H_{21}O_6$	-1.3	MS^2 [381]: 366 (76), 363 (19), 351 (19), 348 (29), 339 (13), 330 (8.0), 211 (9.5), 197 (100), 183 (23), 136 (4.8); MS^3 [381 → 197]: 182 (15), 164 (40), 143 (14)	-	A

Scheme S1. Fragmentation proposal of compound 1 (m/z 383, rt: 41.1 min, cluster A)

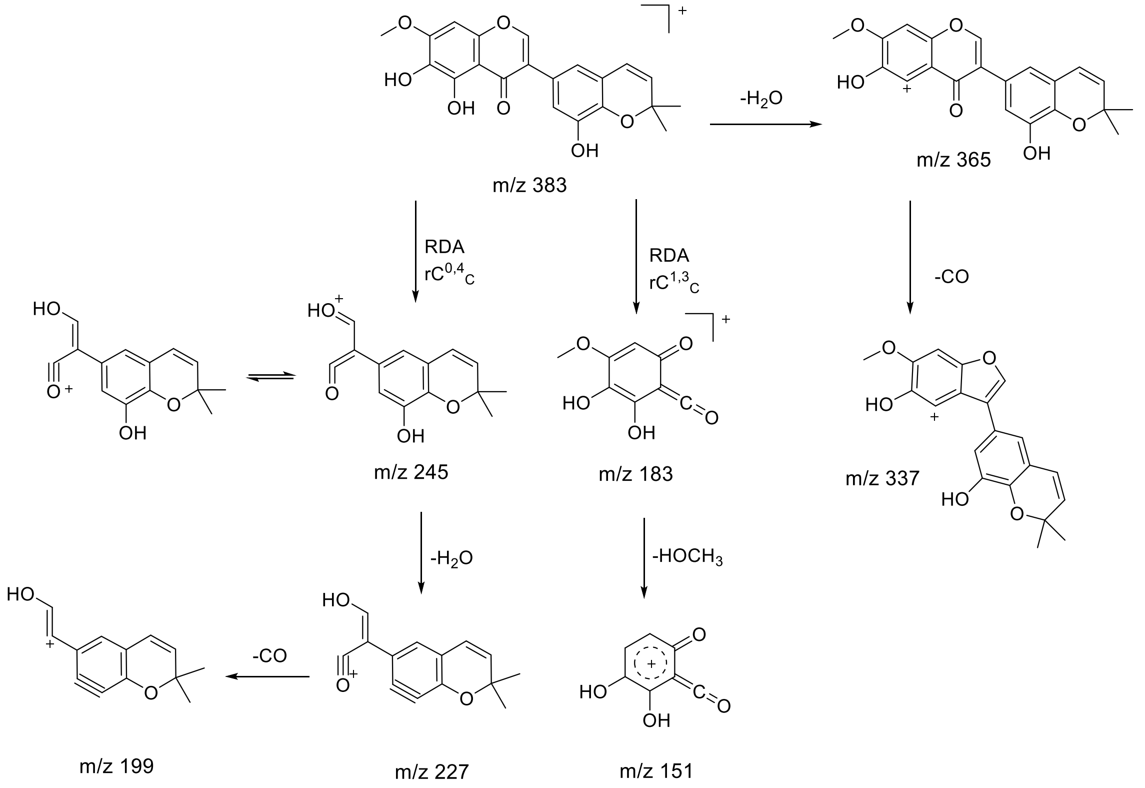

Figure S2. HRESIMS and LC-ESIMS/MS spectrum of compound 1 ($[M + H]^+$, positive mode)

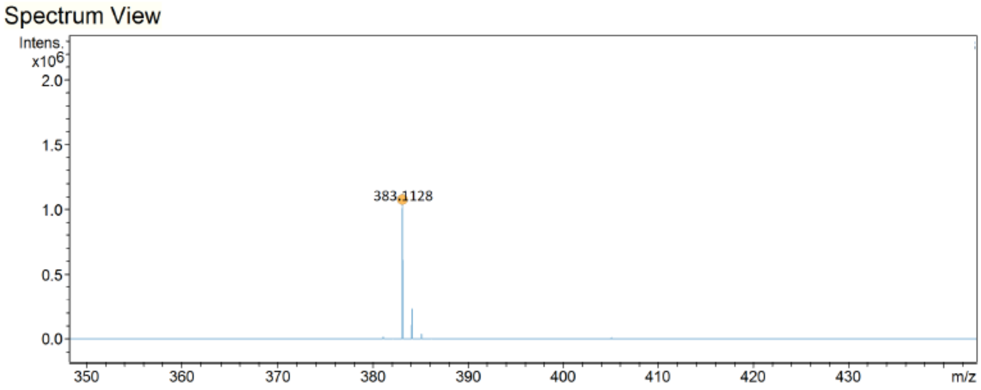

Meas. m/z	#	Ion Formula	m/z	err [ppm]	mSigma	# mSigma	Score	rdb	e⁻ Conf	N-Rule
383.1128	1	C21H19O7	383.1125	-0.6	2.0	1	100.00	12.5	even	ok

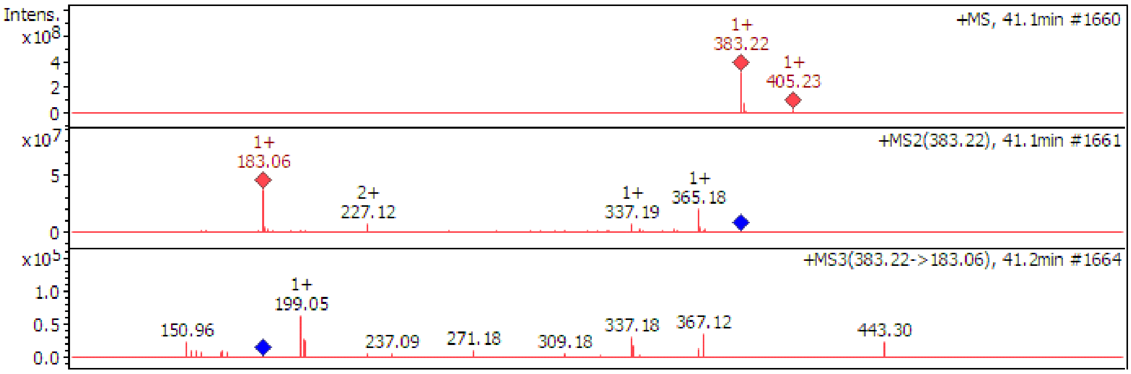

Figure S3. ^{1}H NMR spectrum of 1 at 500 MHz in DMSO-*d6*

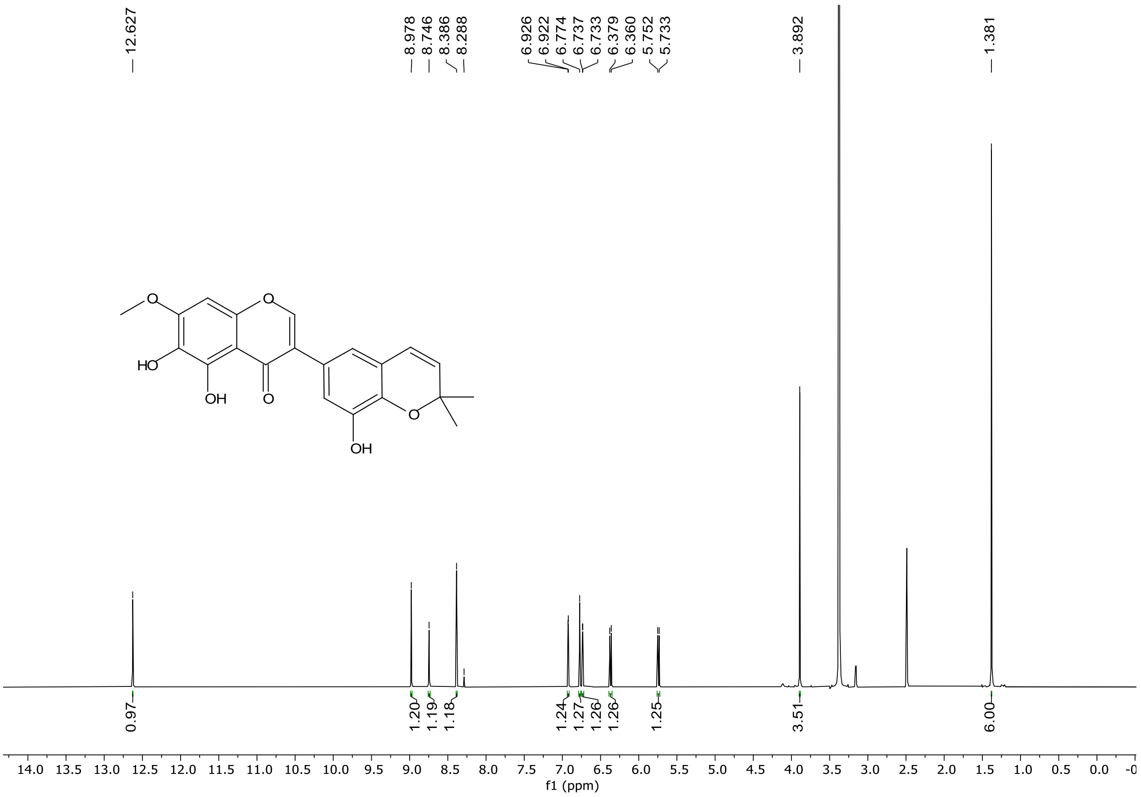

Figure S4. ^{1}H NMR spectrum of 1 at 500 MHz in DMSO-*d6* (expansion: 5.5 – 9.3 ppm)

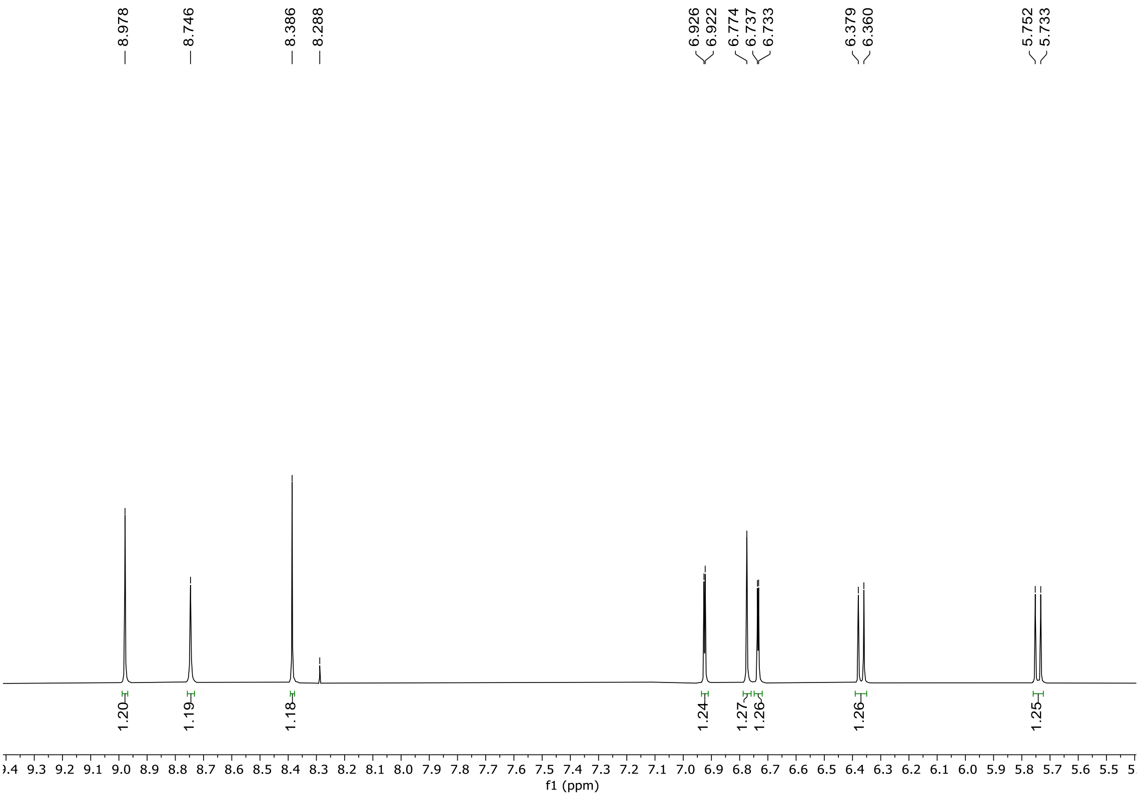

Figure S5. ^{13}C NMR spectrum of 1 at 125 MHz in DMSO-*d6*

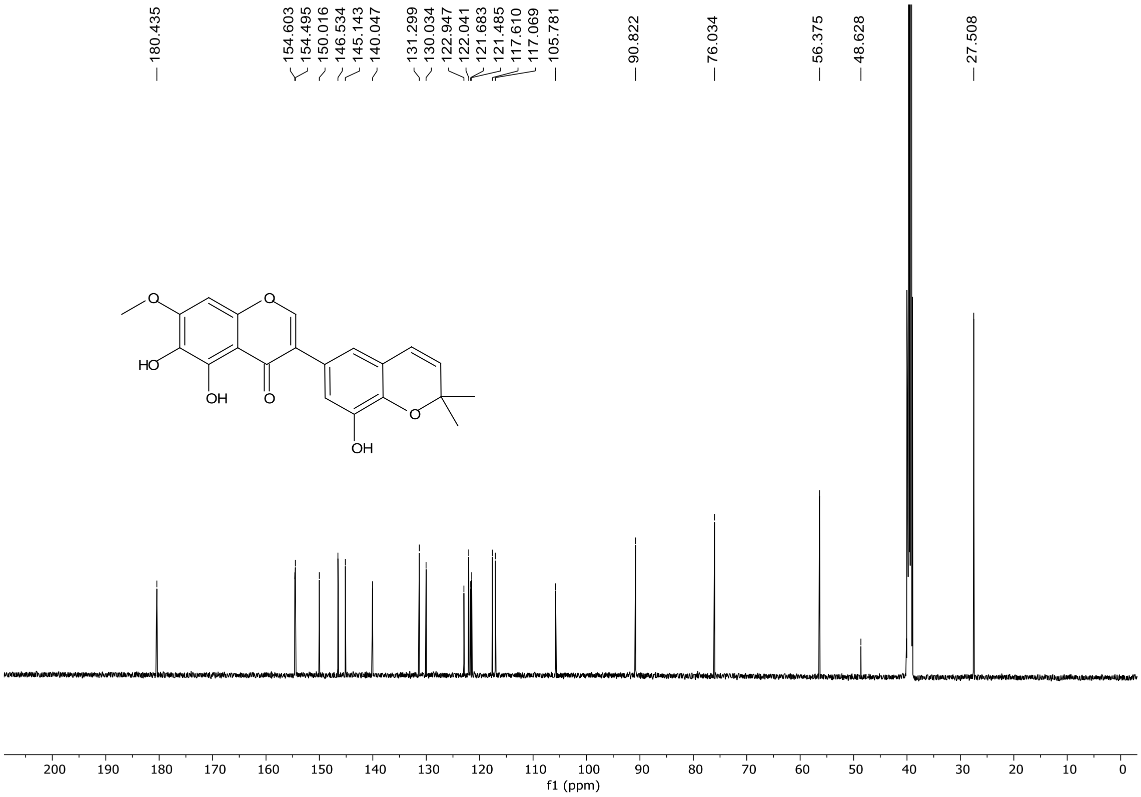

Figure S6. ^{13}C NMR spectrum of 1 at 125 MHz in DMSO-*d6* (expansion: 115 – 155 ppm)

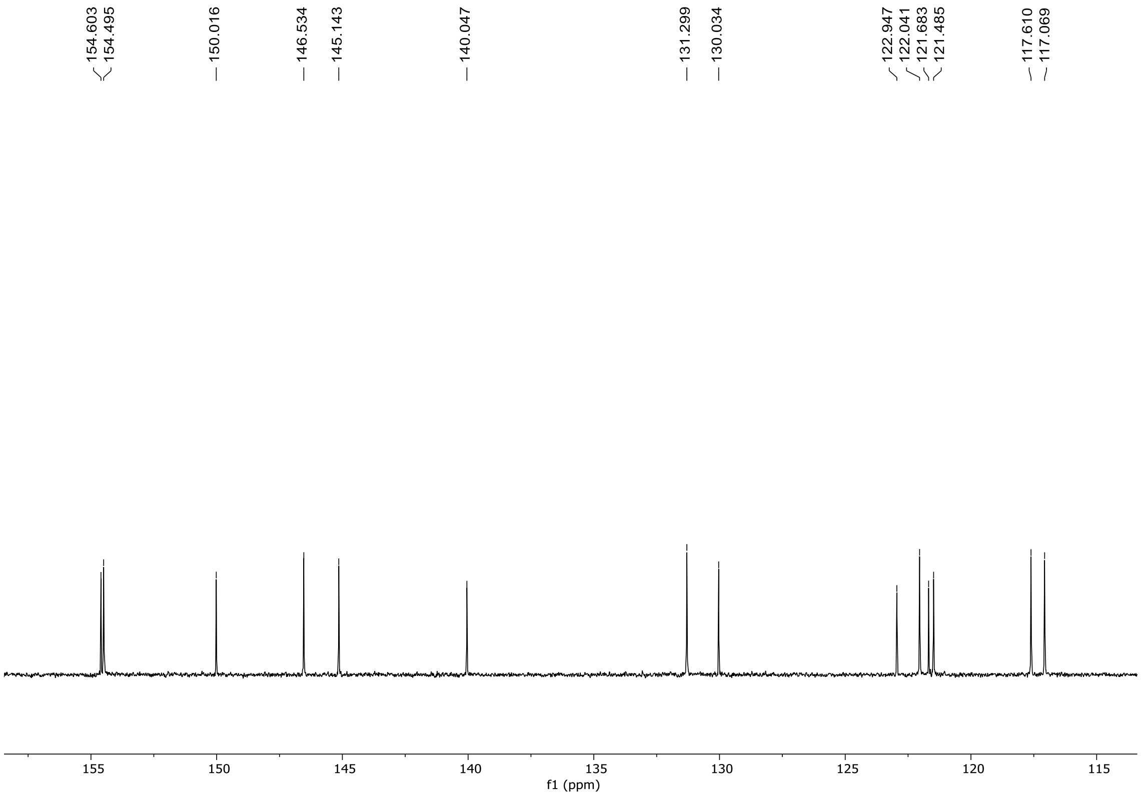

Figure S7. ^{13}C NMR DEPT135 spectrum of 1 at 125 MHz in DMSO-*d6*

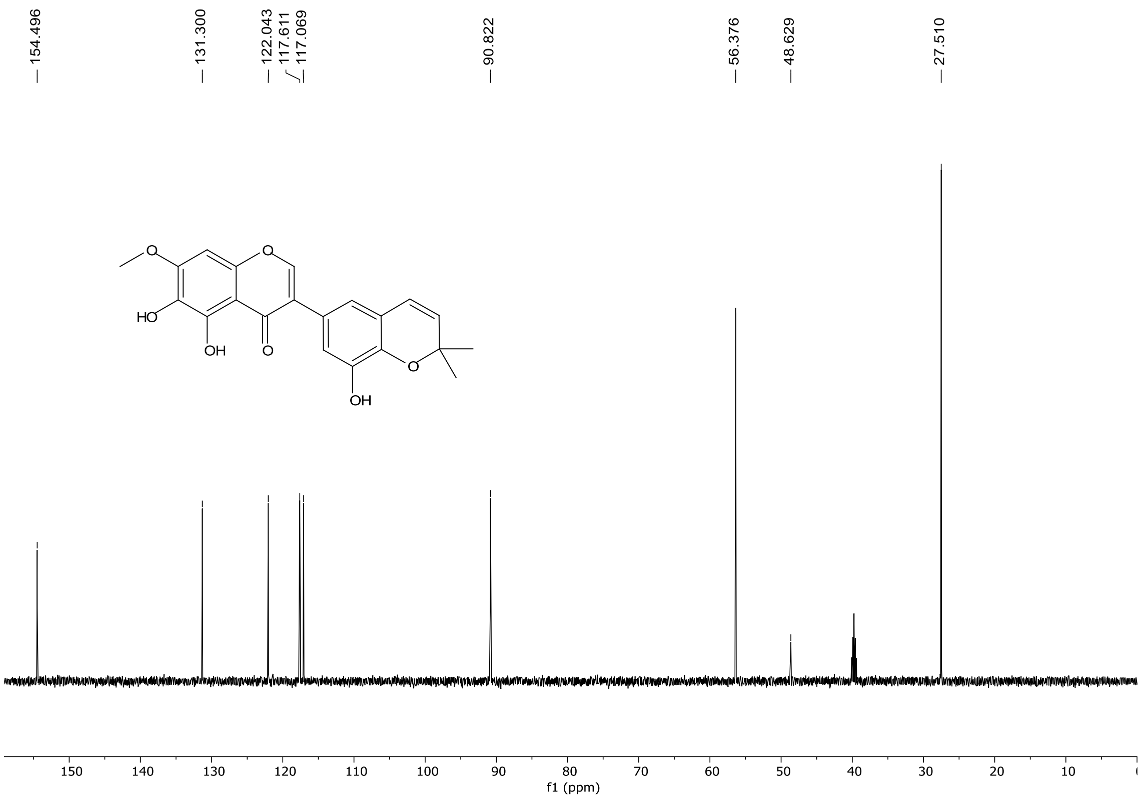

Figure S8. HSQC spectrum of 1 at 500 MHz in DMSO-*d6*

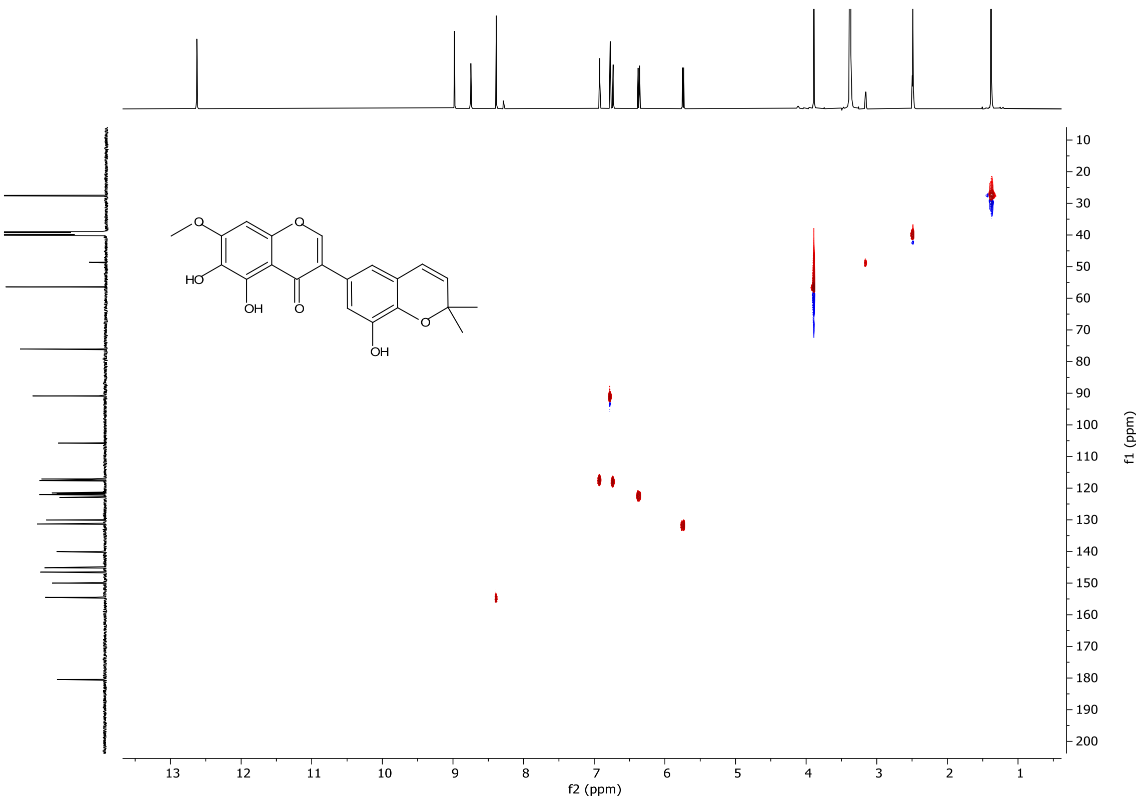

Figure S9. HMBC spectrum of 1 at 500 MHz in DMSO-*d6*

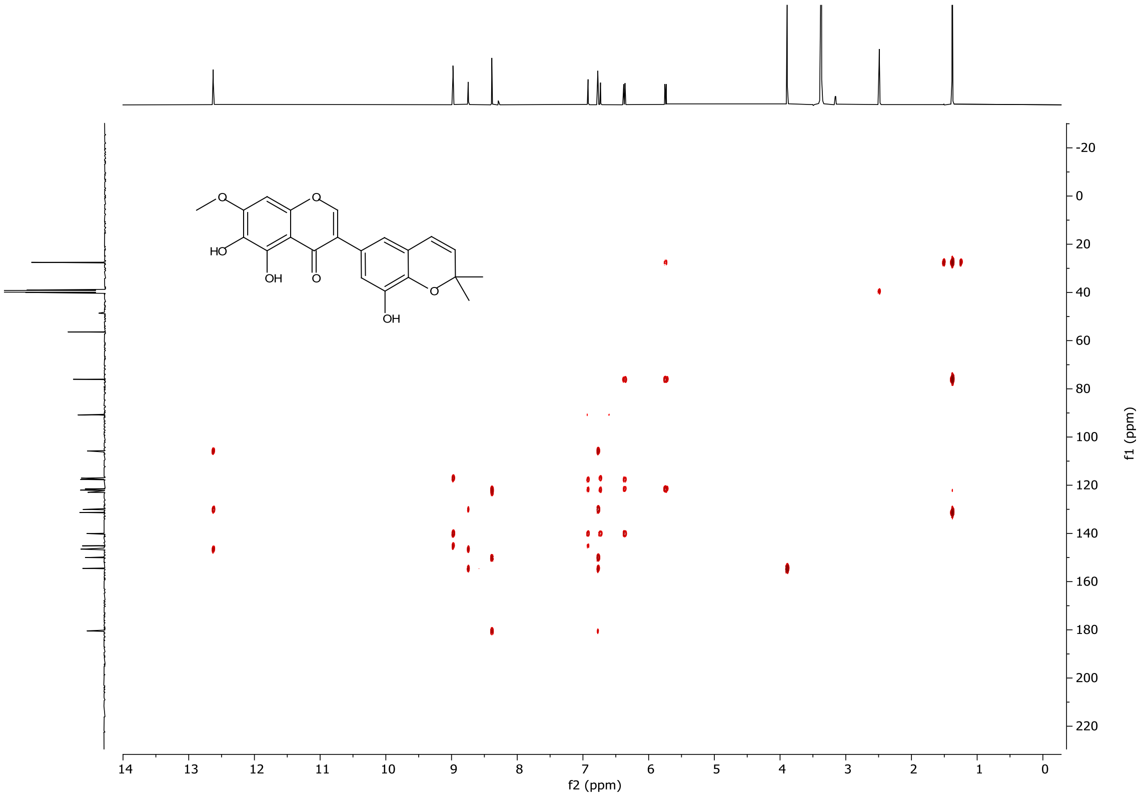

Figure S10. COSY spectrum of 1 at 500 MHz in DMSO-*d6*

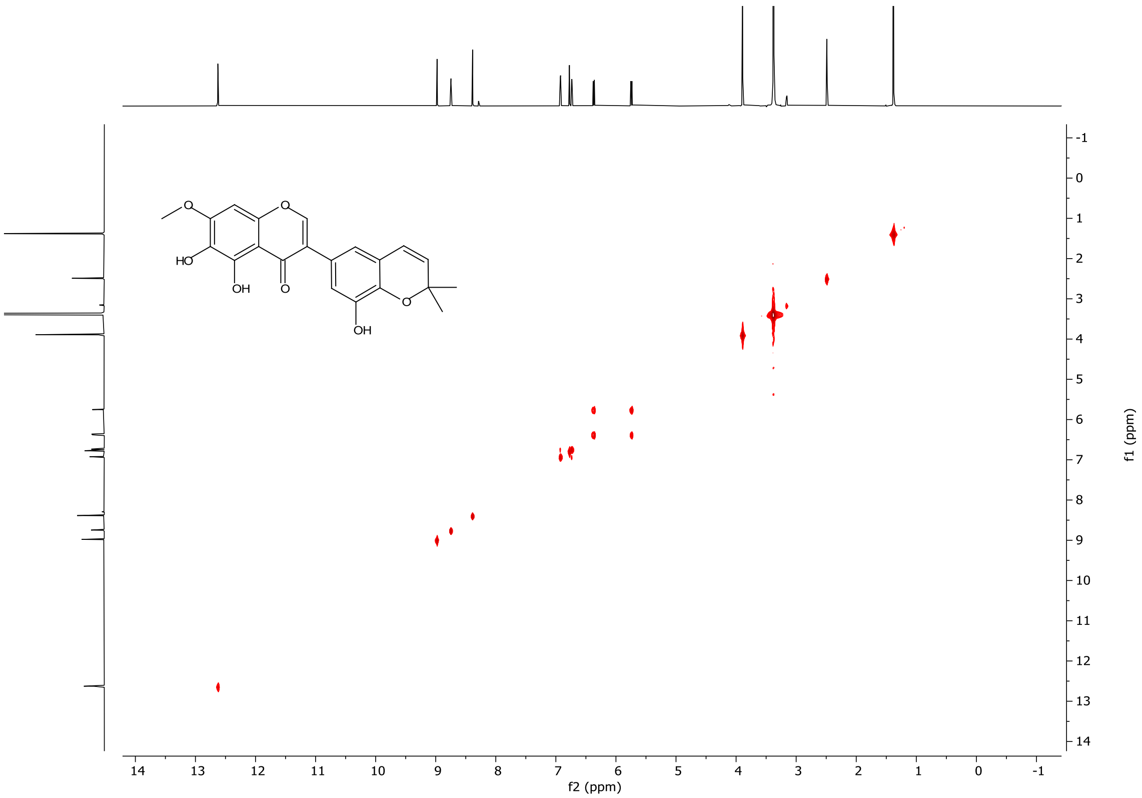

Figure S11. IR spectrum of 1 (liquid solution)

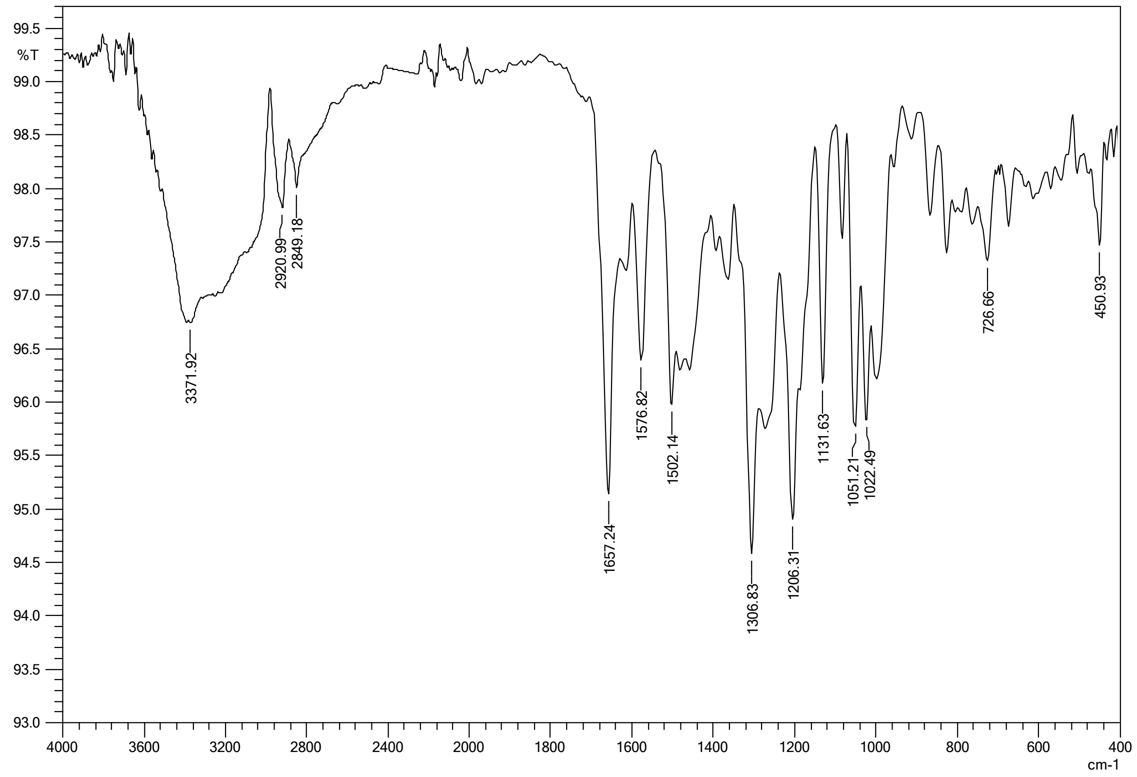

Scheme S2. Fragmentation proposal of compound 2 (m/z 397, rt: 44.9 min, cluster A)

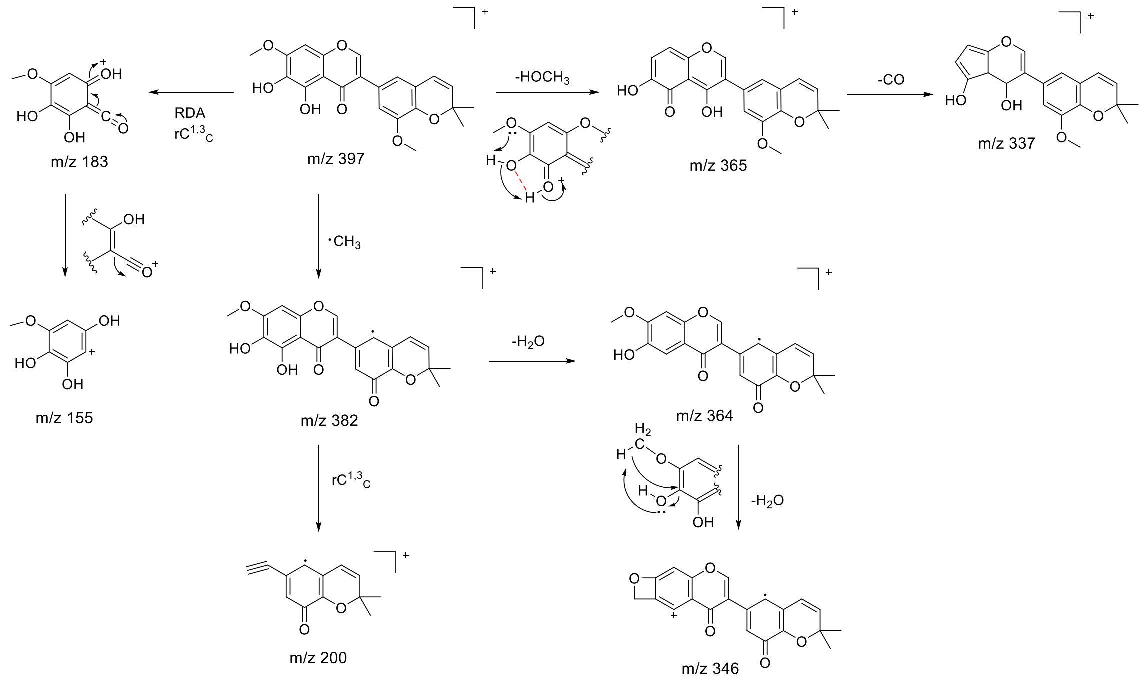

Figure S12. HRESIMS and LC-ESIMS/MS spectrum of 2 ([M + H]$^{+}$, positive mode)

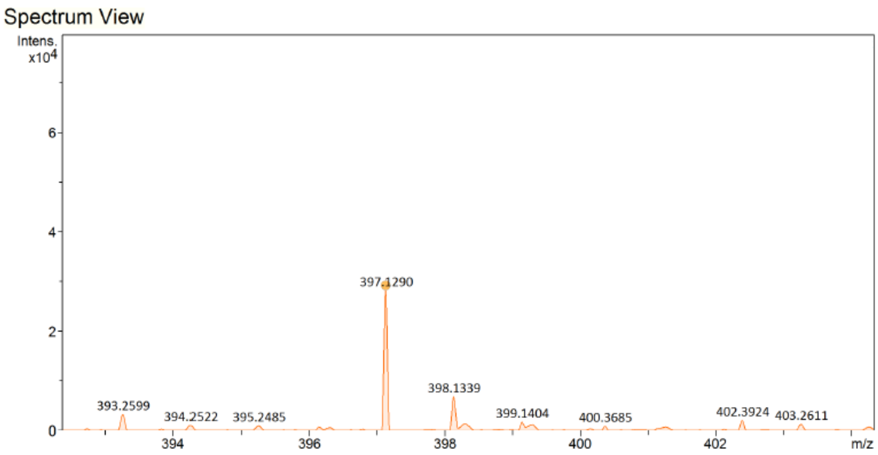

Meas. m/z	#	Ion Formula	m/z	err [ppm]	mSigma	# mSigma	Score	rdb	e⁻ Conf	N-Rule
397.1290	1	C22H21O7	397.1282	-2.0	9.4	1	100.00	12.5	even	ok

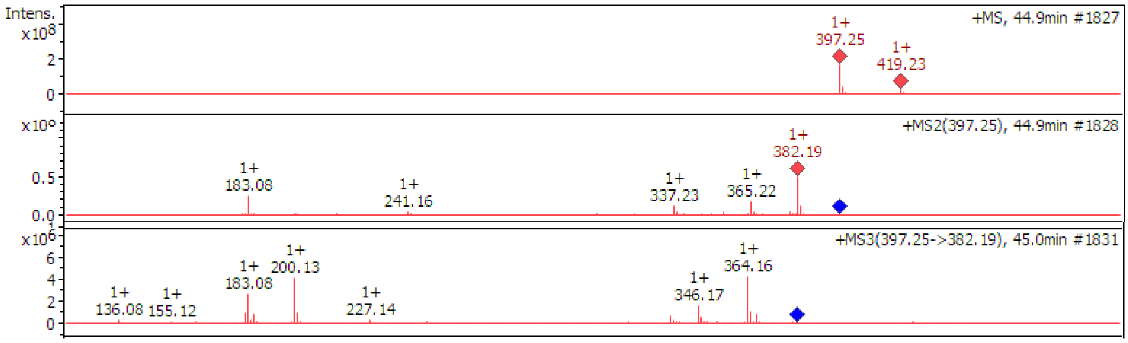

Figure S13. ^{1}H NMR spectrum of 2 at 400 MHz in $CDCl_3$

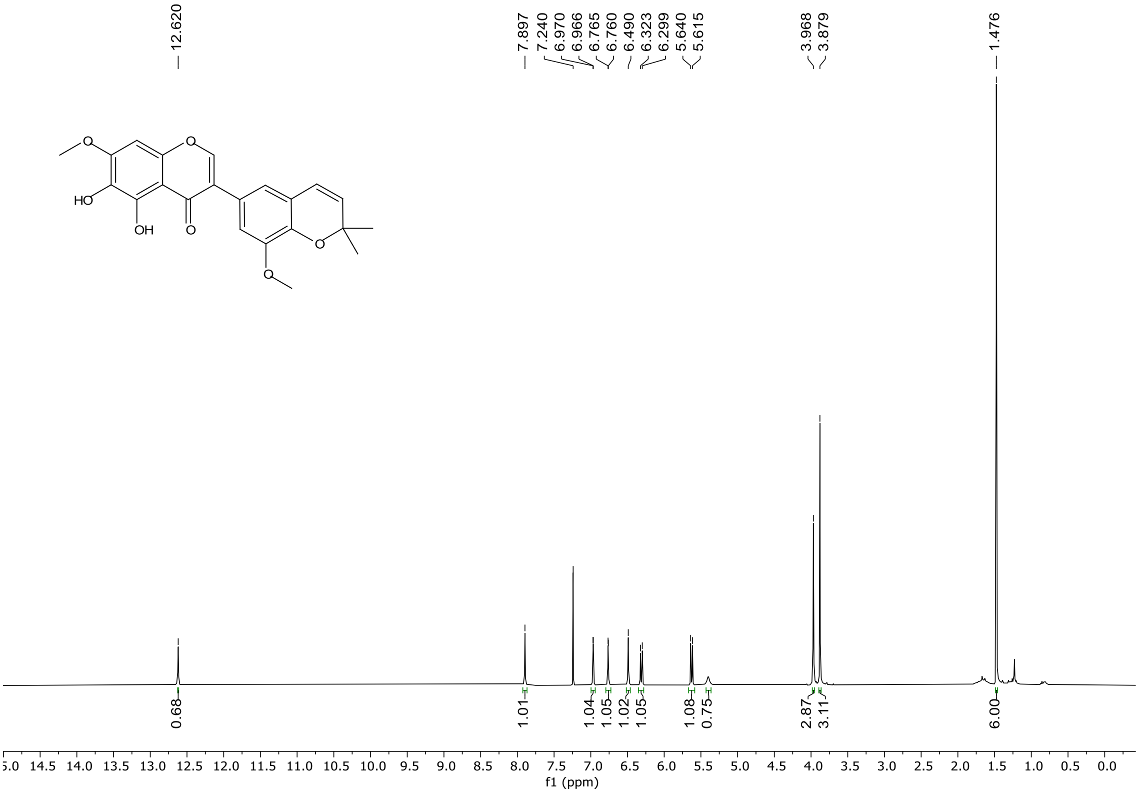

Figure S14. ^{1}H NMR spectrum of 2 at 400 MHz in $CDCl_3$ (expansion: 3.6 – 8.8 ppm)

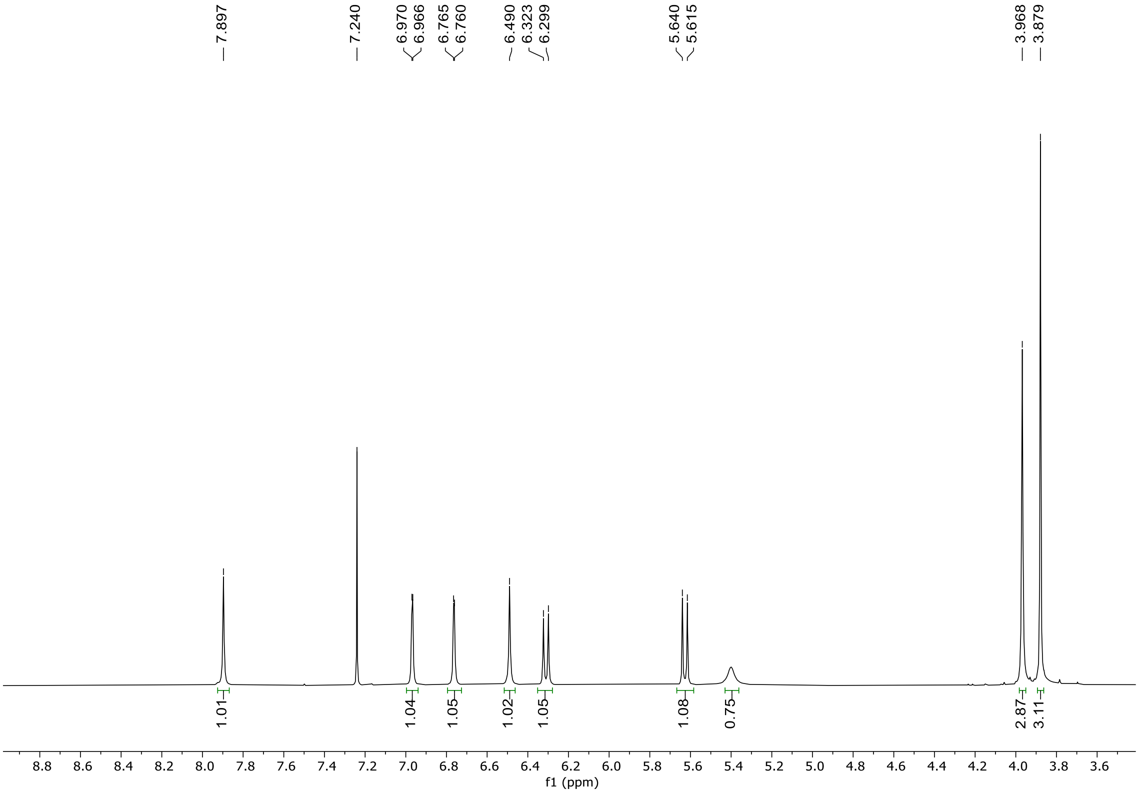

Figure S15. ^{13}C NMR spectrum of 2 at 100 MHz in CDCl$_3$

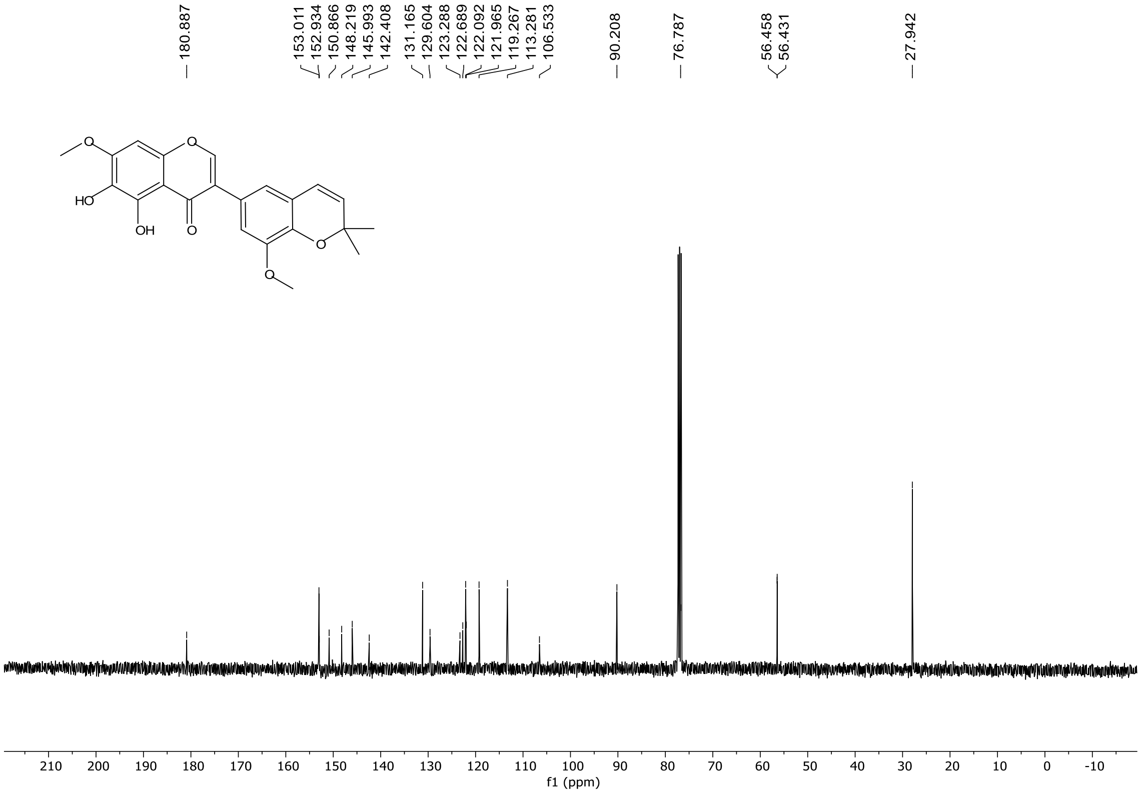

Figure S16. ^{13}C NMR spectrum of 2 at 100 MHz in CDCl$_3$ (expansion: 106 - 156 ppm)

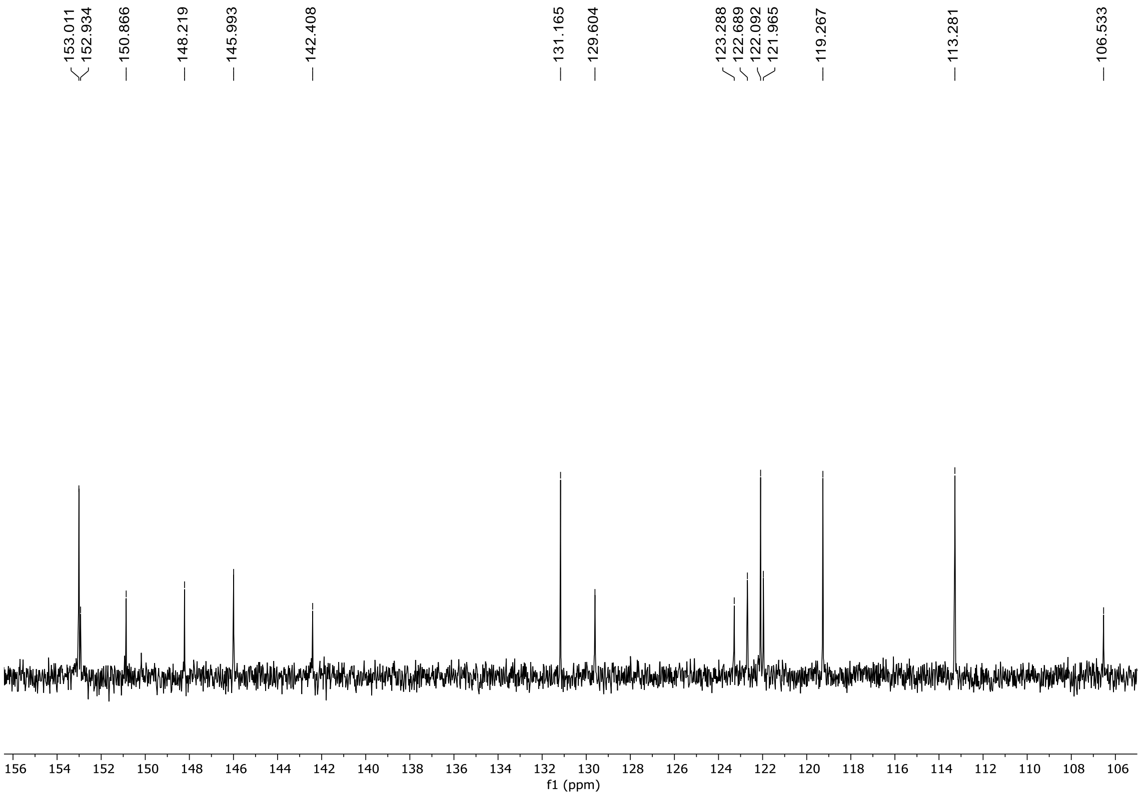

Figure S17. DEPT-135 spectrum of 2 at 100 MHz in $CDCl_3$

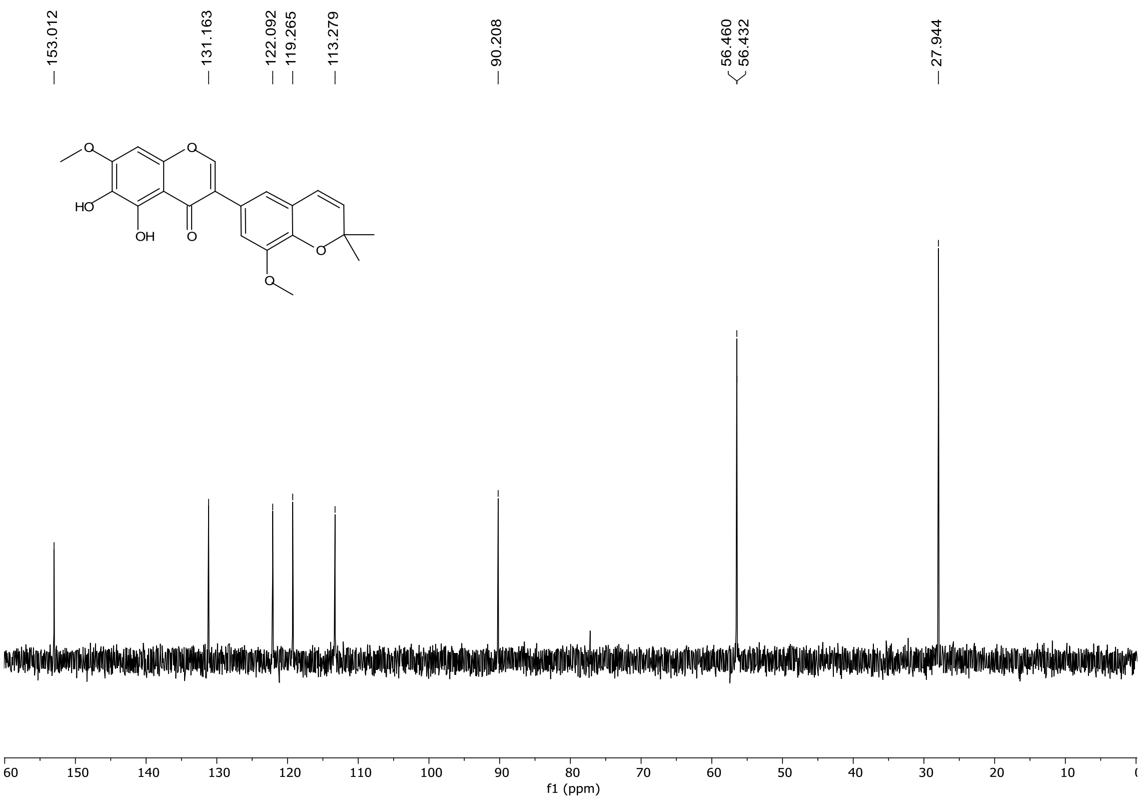

Figure S18. HSQC spectrum of 2 at 400 MHz in $CDCl_3$

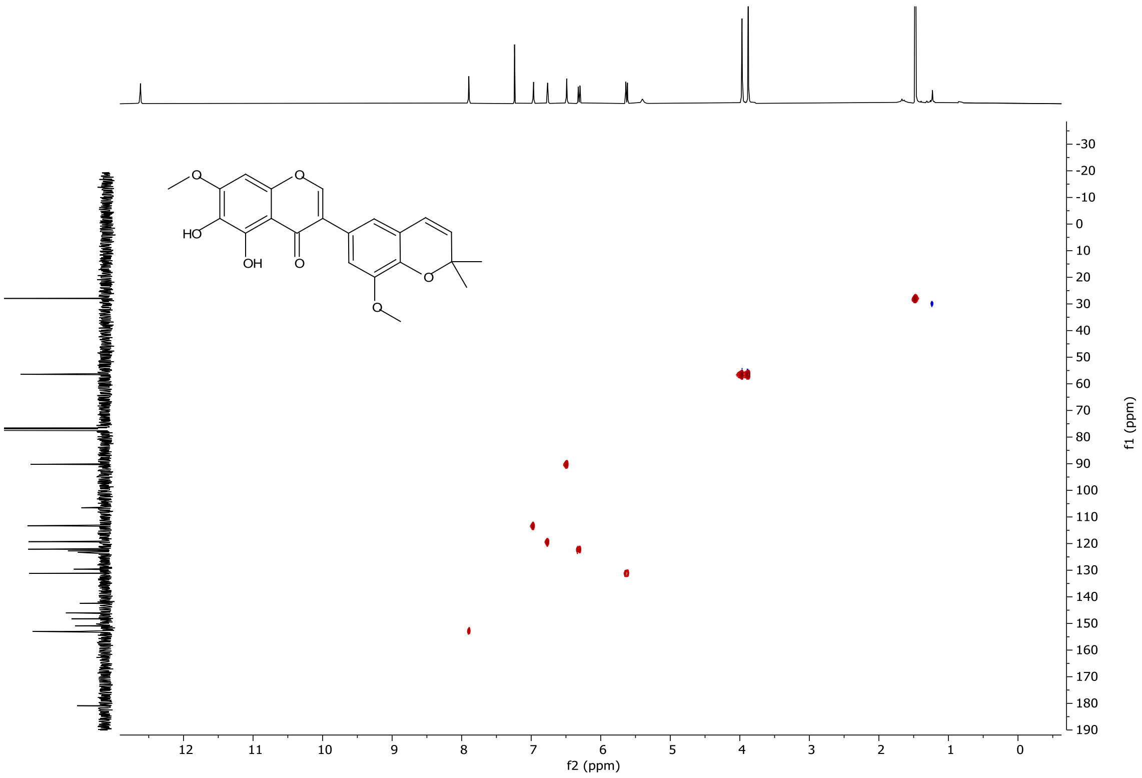

Figure S19. HMBC spectrum of 2 at 400 MHz in $CDCl_3$

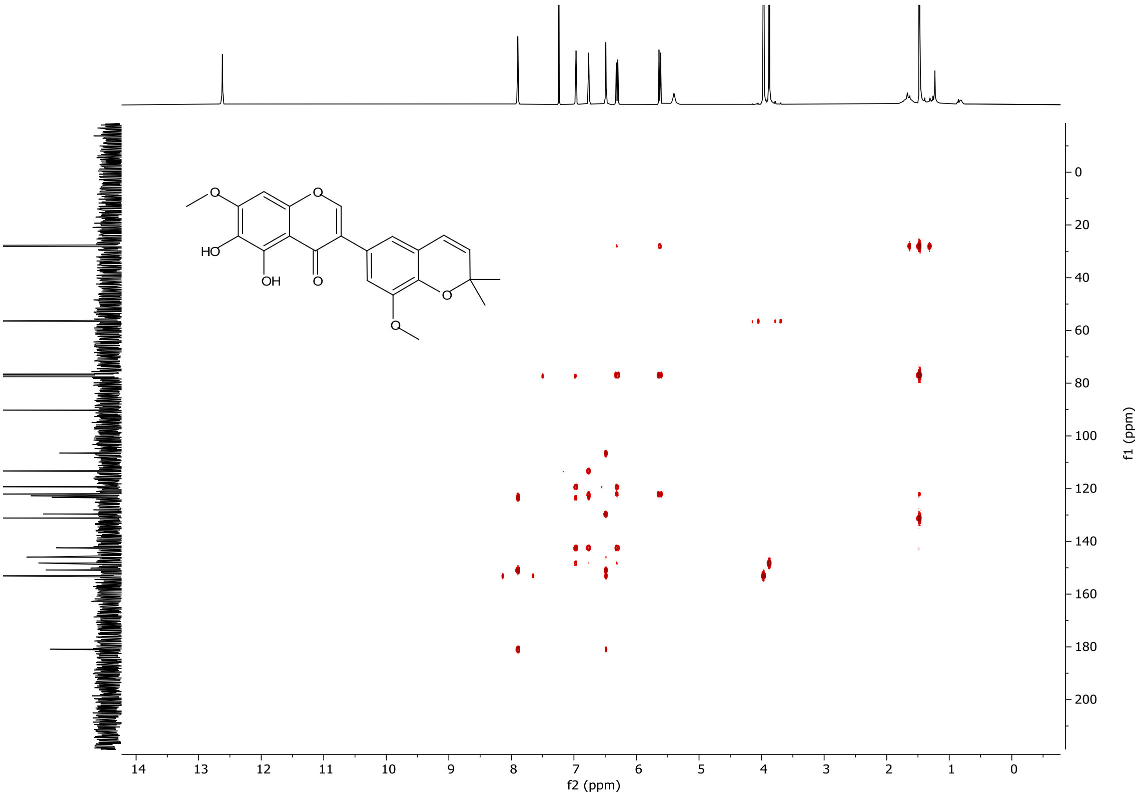

Figure S20. COSY spectrum of 2 at 400 MHz in $CDCl_3$

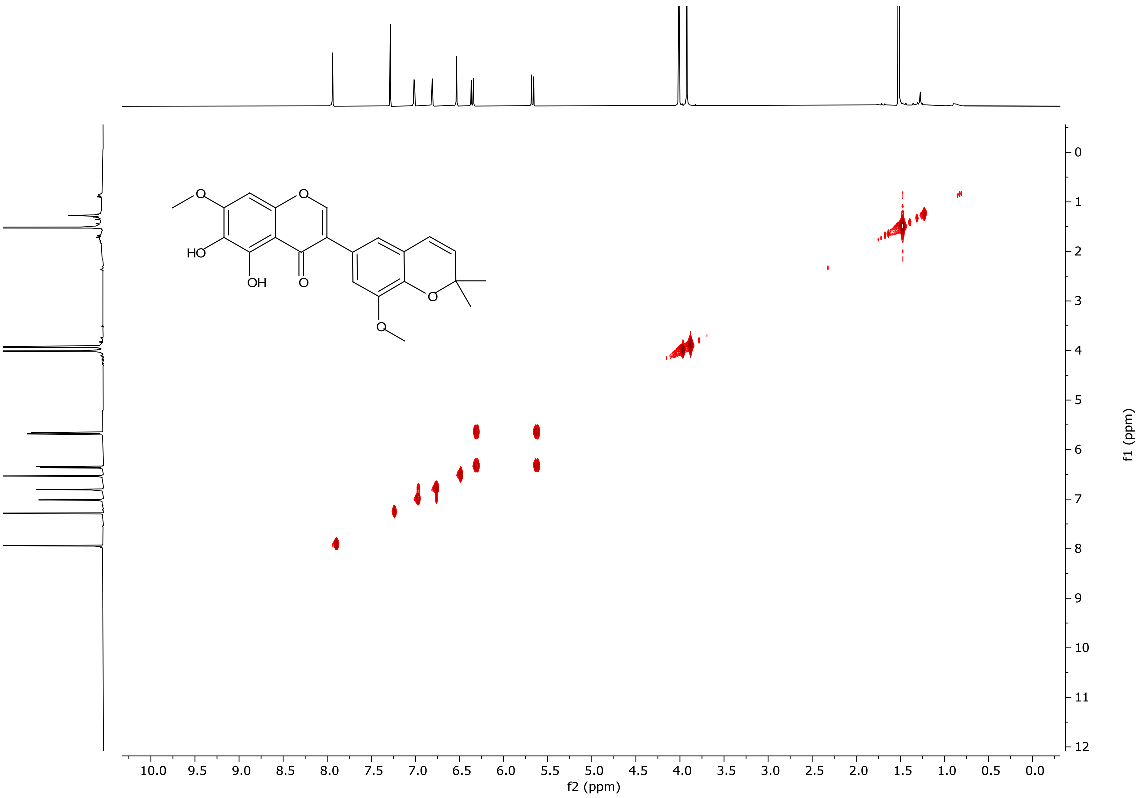

Figure S21. NOESY spectrum of 2 at 400 MHz in $CDCl_3$

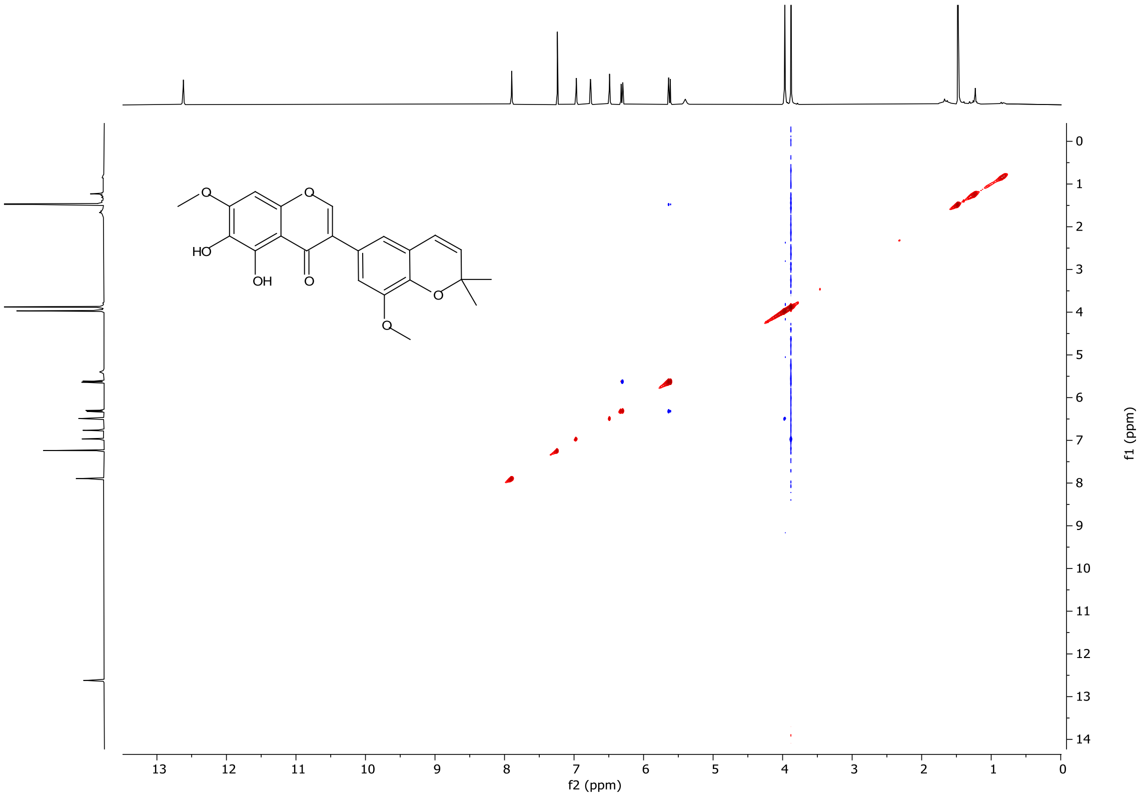

Figure S22. IR spectrum of 2 (liquid solution)

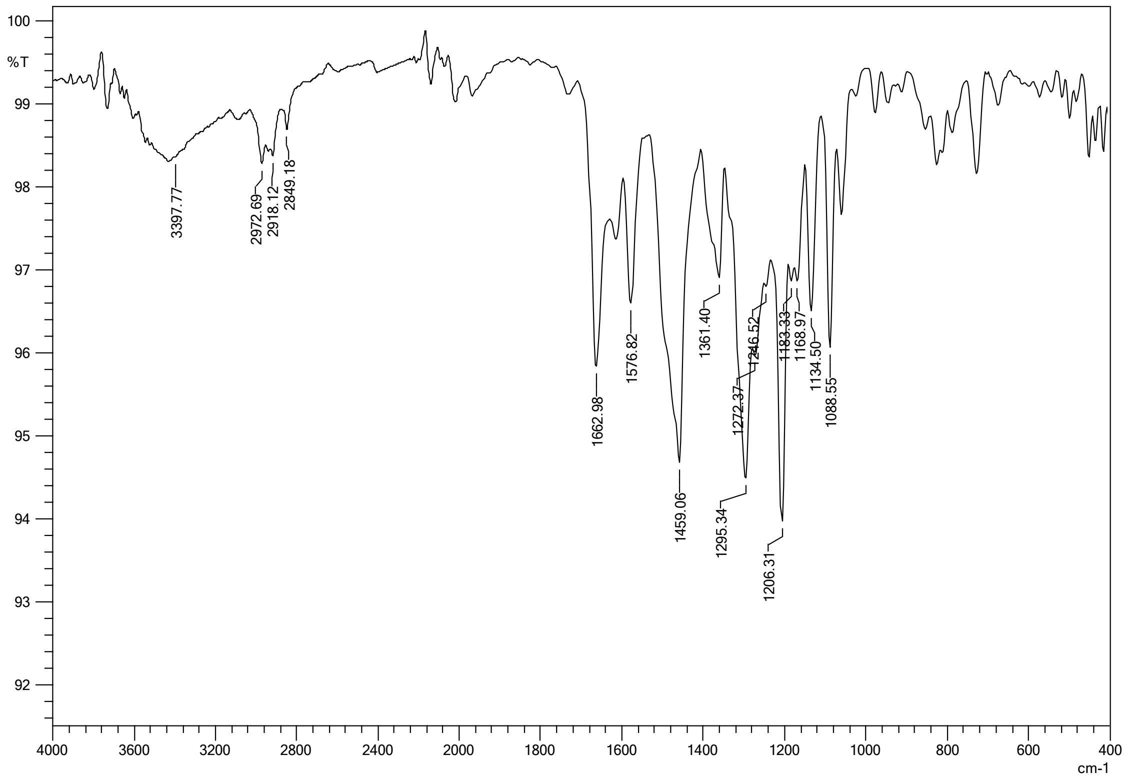

Scheme S3. Fragmentation proposal of compound 3 (*m/z* 397, rt: 47.4 min, cluster A)

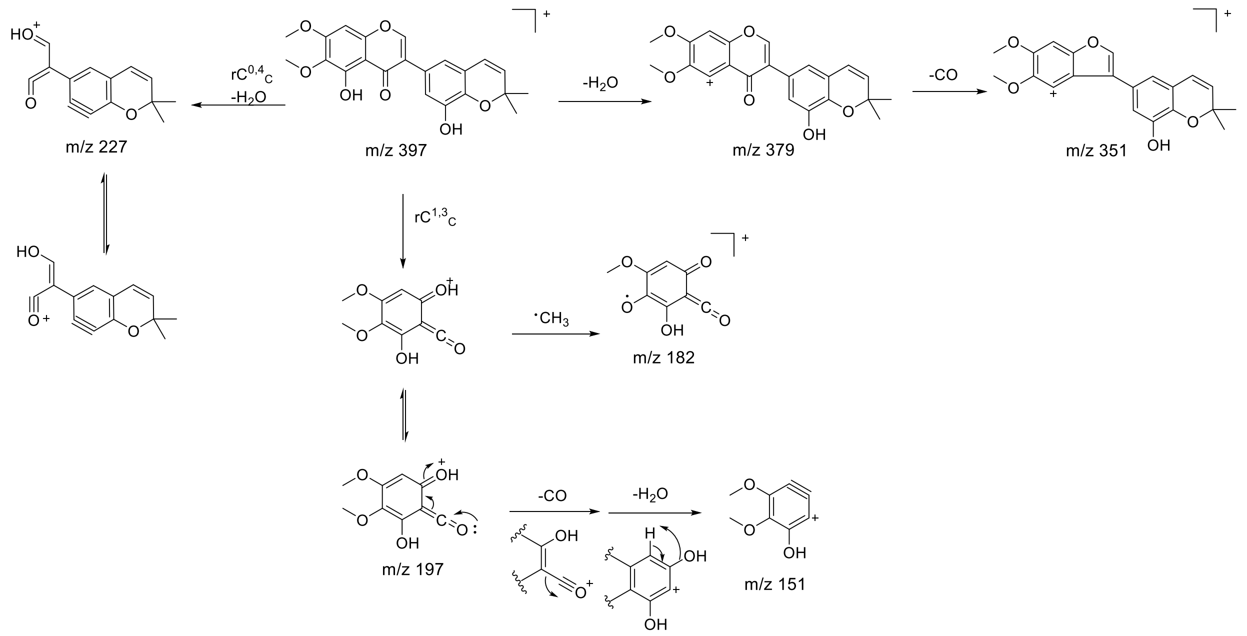

Figure S23. HRESIMS and LC-ESIMS/MS spectrum of compound 3 ([M + H] +, positive mode)

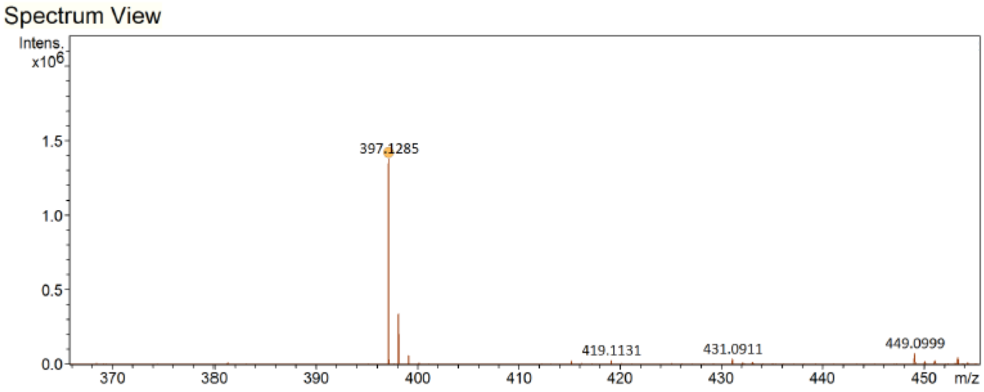

Meas. m/z	#	Ion Formula	m/z	err [ppm]	mSigma	# mSigma	Score	rdb	e⁻ Conf	N-Rule
397.1285	1	C22H21O7	397.1282	-0.9	2.4	1	100.00	12.5	even	ok

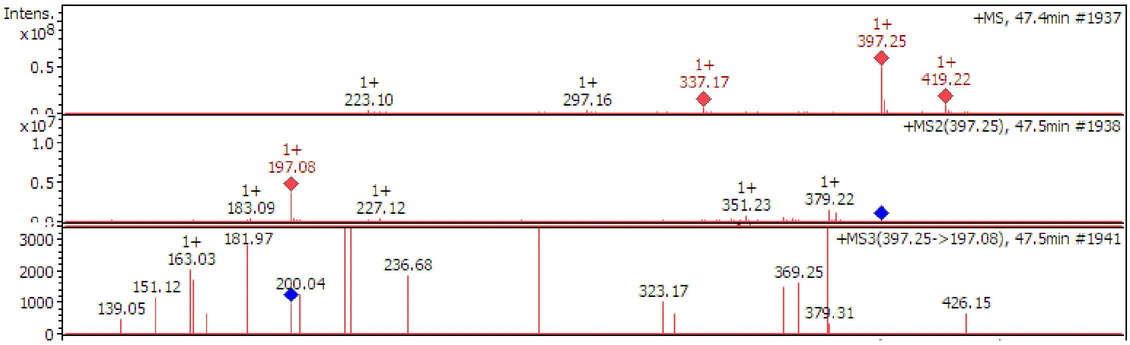

Figure S24. ^{1}H NMR spectrum of 3 at 400 MHz in $CDCl_3$

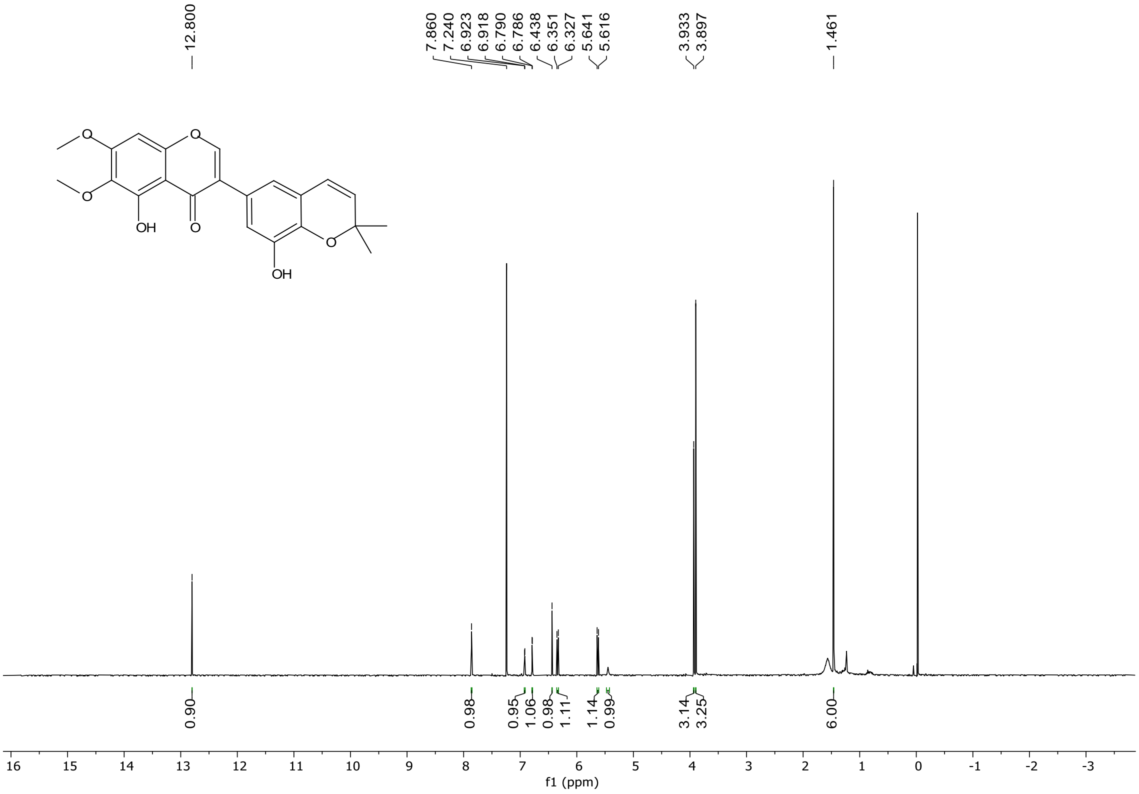

Figure S25. ^{1}H NMR spectrum of 3 at 400 MHz in $CDCl_3$ (4.0 – 8.5 ppm)

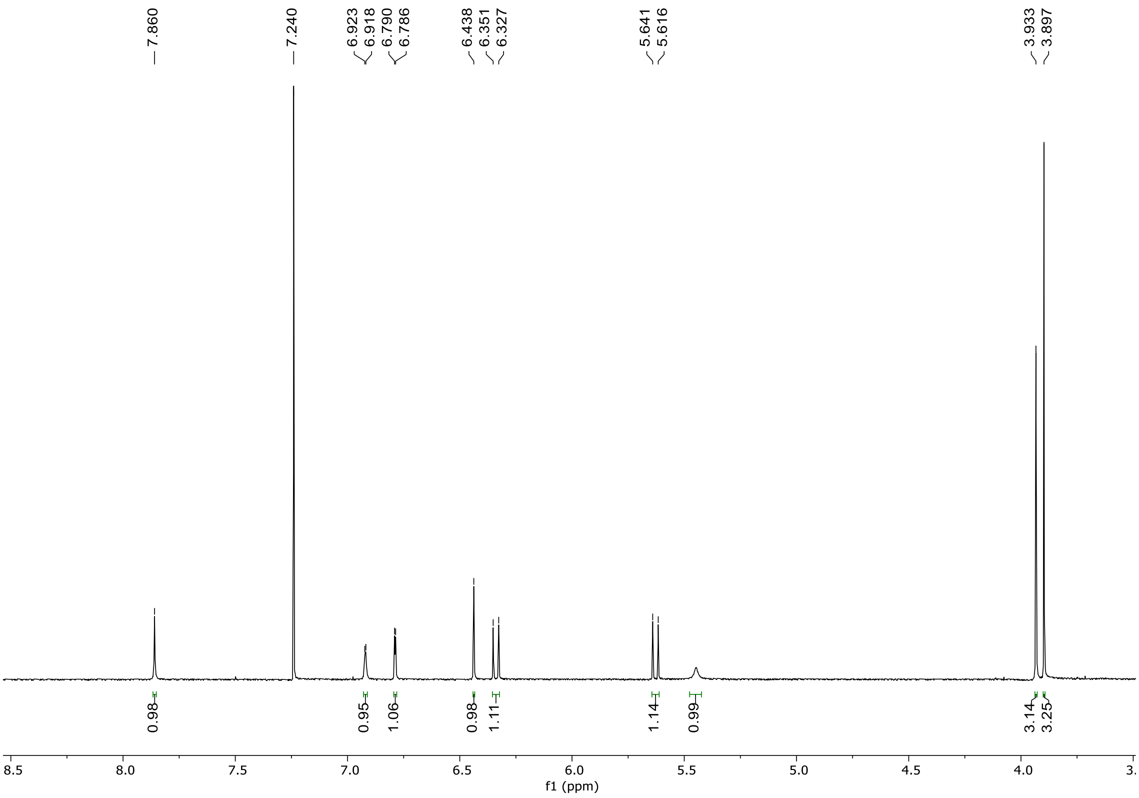

Figure S26. ^{13}C NMR spectrum of 3 at 400 MHz in $CDCl_3$

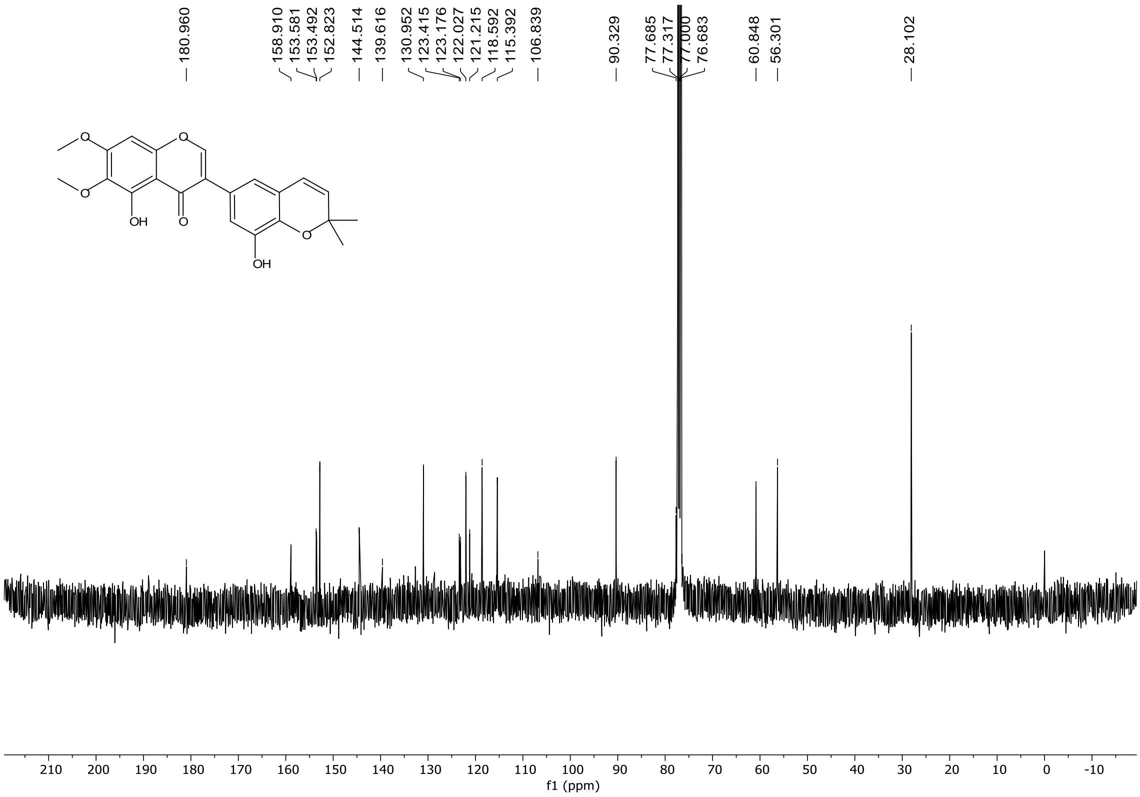

Figure S27. ^{13}C NMR spectrum of 3 at 400 MHz in $CDCl_3$ (expansion: 102 - 164 ppm)

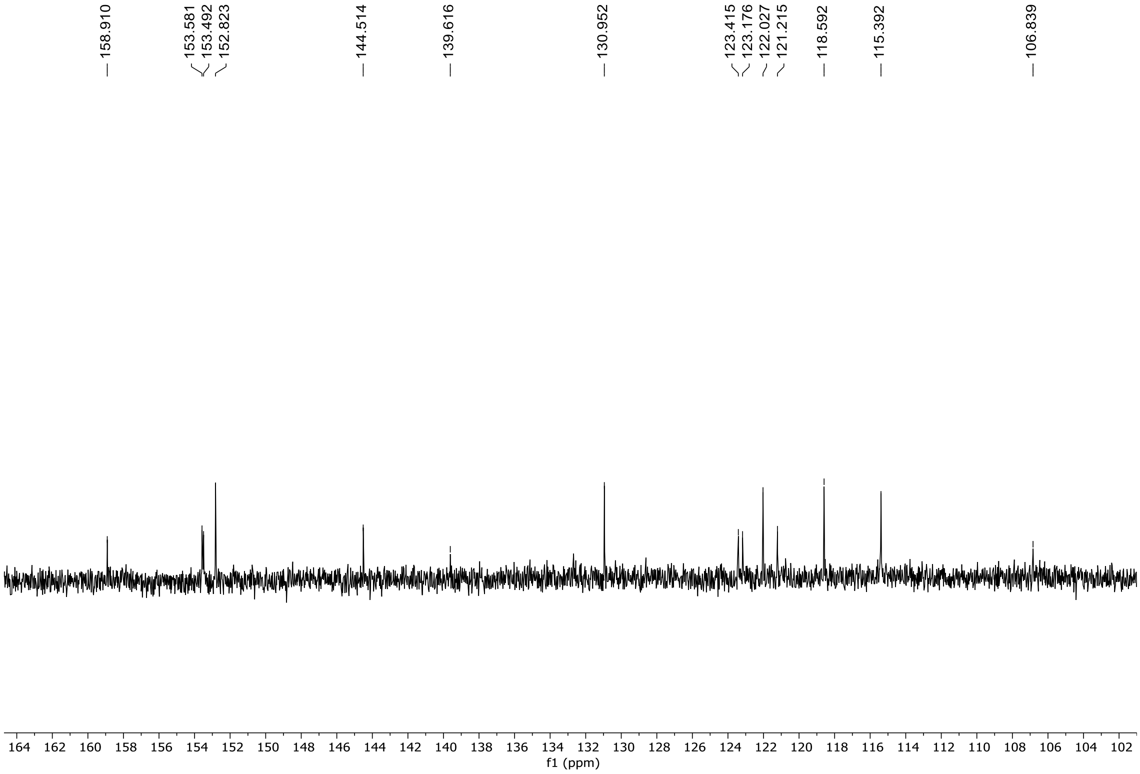

Figure S28. HSQC spectrum of 3 at 400 MHz in $CDCl_3$

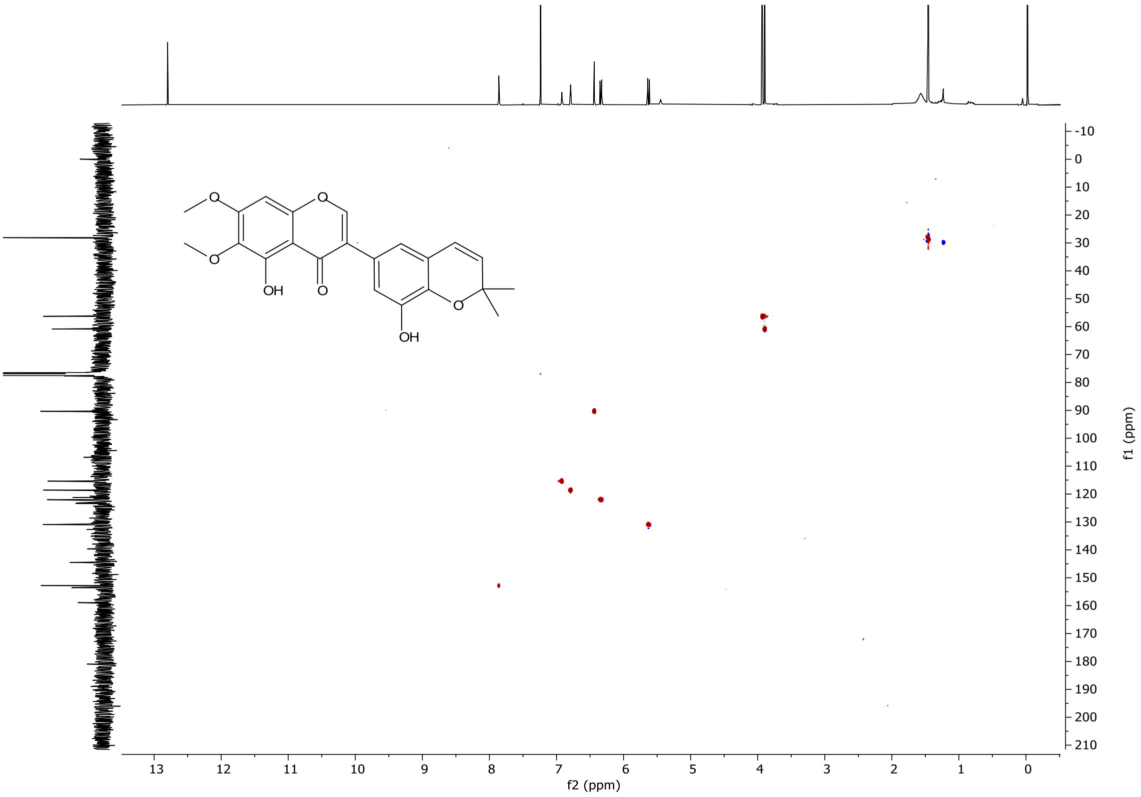

Figure S29. HMBC spectrum of 3 at 400 MHz in $CDCl_3$

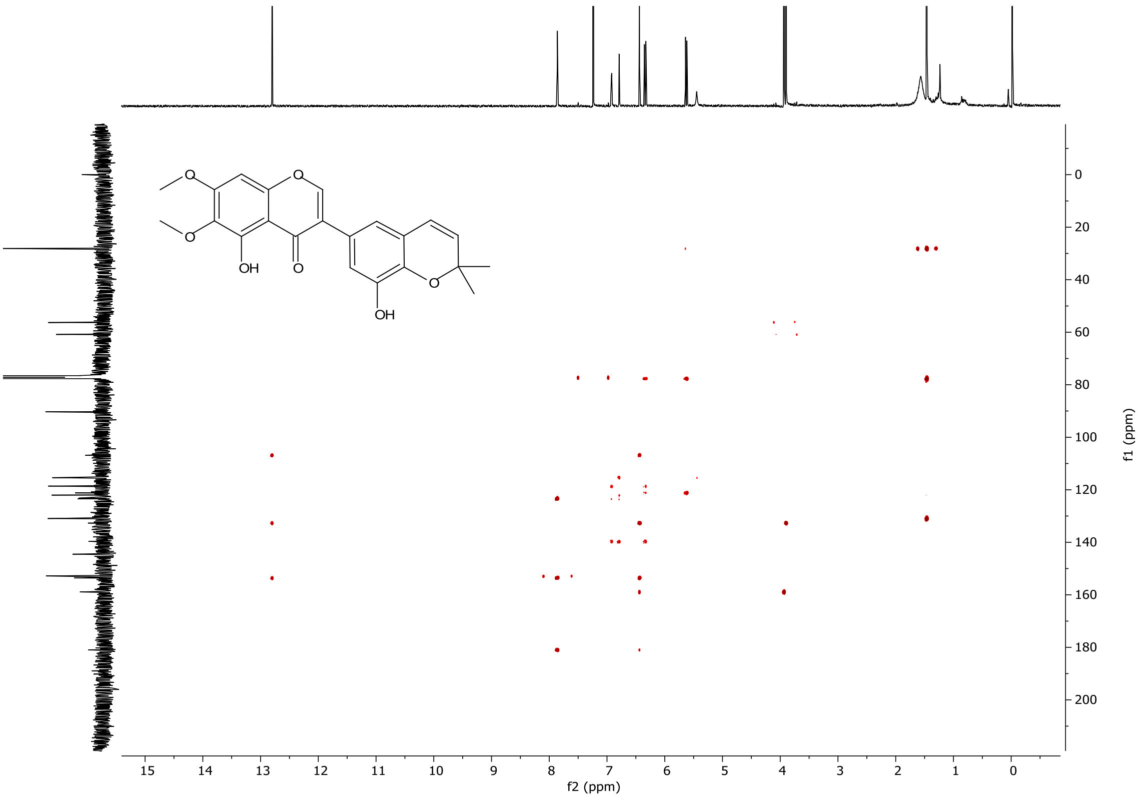

Figure S30. NOESY spectrum of 3 at 400 MHz in CDCl$_3$

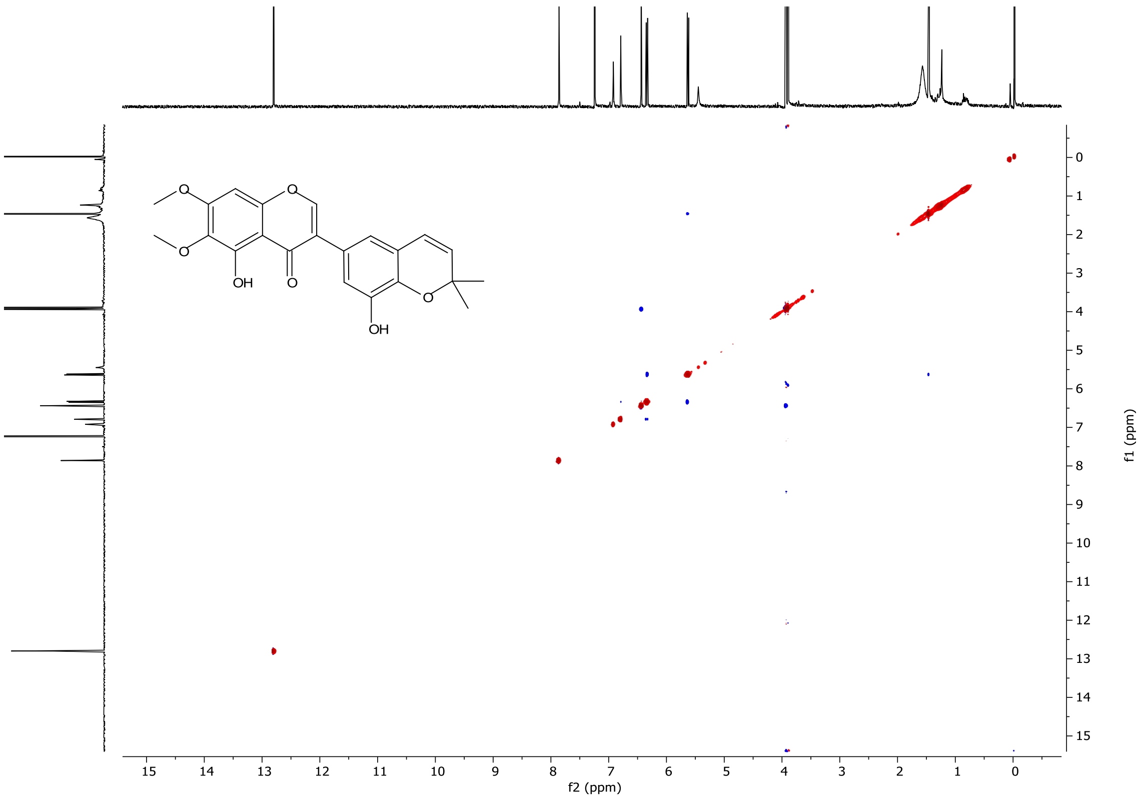

Figure S31. COSY spectrum of 3 at 400 MHz in CDCl$_3$

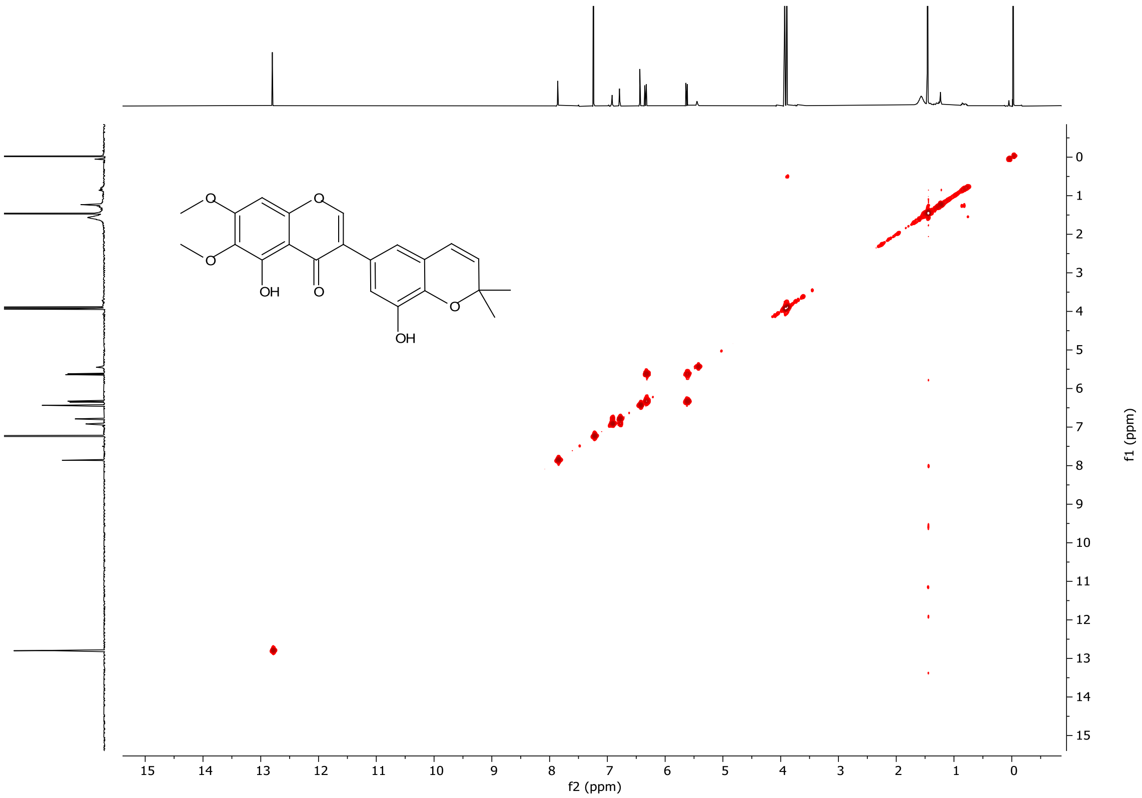

Figure S32. IR spectrum of 3 (liquid solution)

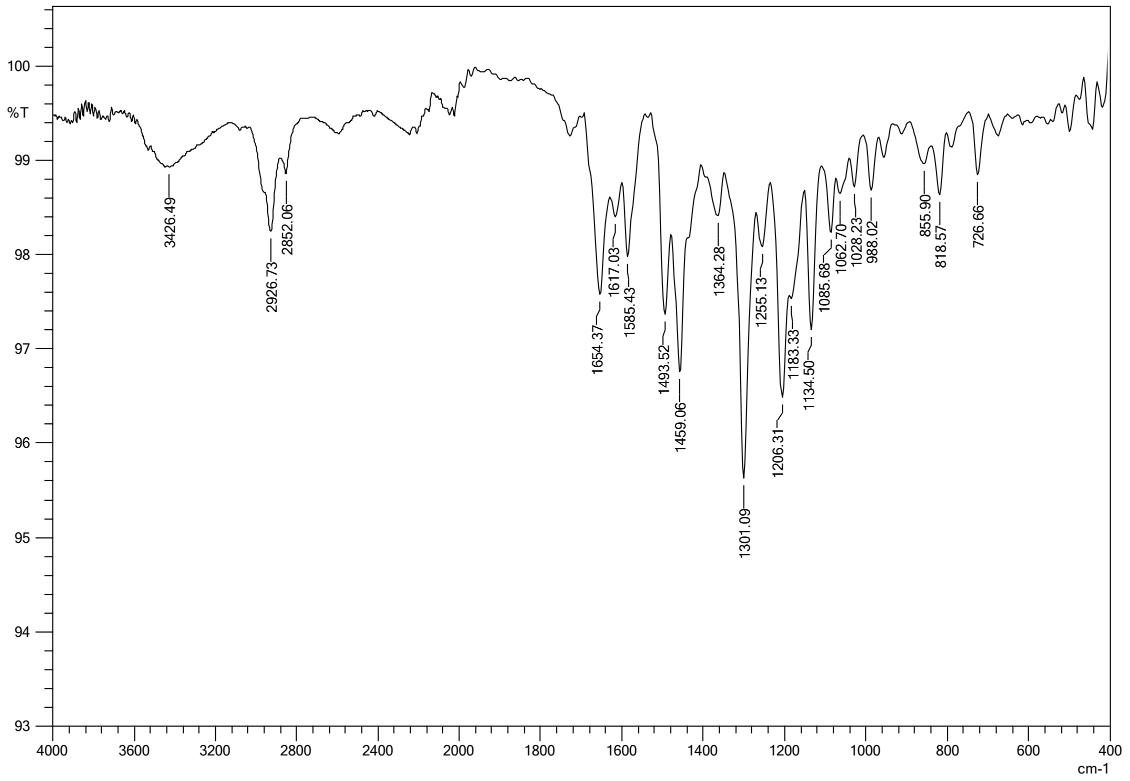

Scheme S4. Fragmentation proposal of compound 4 (m/z 411, rt: 51.8 min, cluster A)

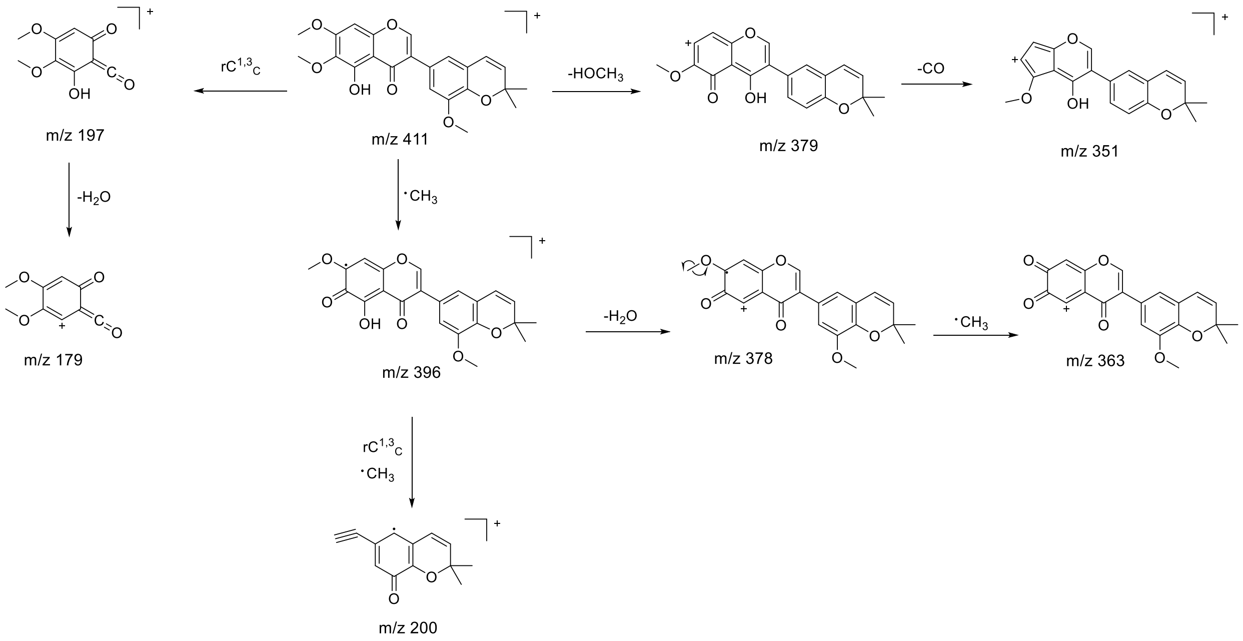

Figure S33. HRESIMS and LC-ESIMS/MS spectrum of compound 4 ([M + H] +, positive mode)

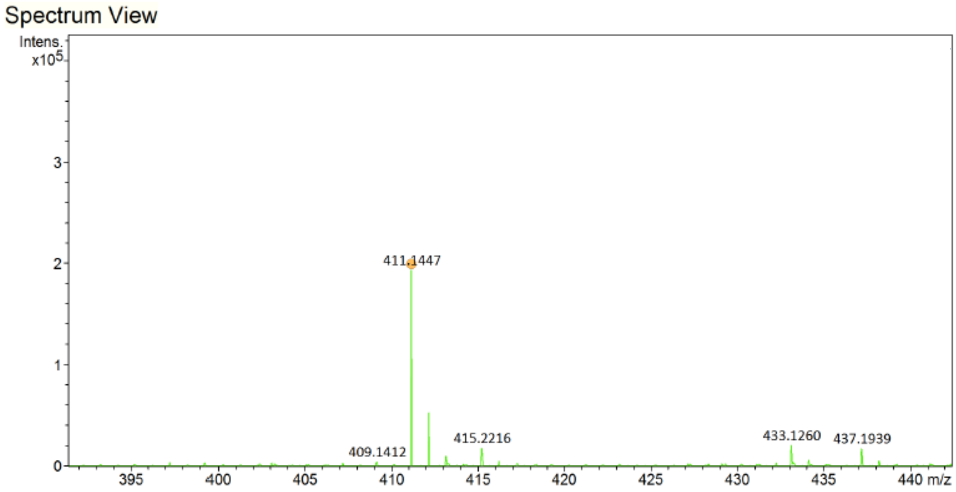

Meas. m/z	#	Ion Formula	m/z	err [ppm]	mSigma	# mSigma	Score	rdb	e⁻ Conf	N-Rule
411.1447	1	C23H23O7	411.1438	-2.2	11.9	1	100.00	12.5	even	ok

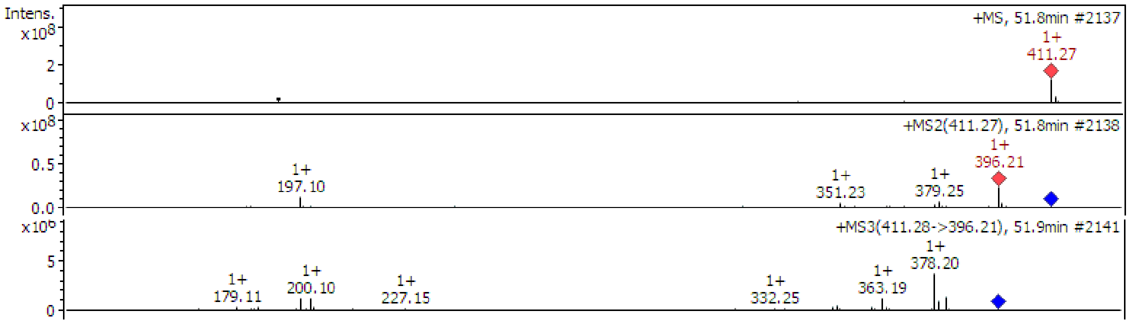

Figure S34. ^{1}H NMR spectrum of 4 at 500 MHz in $CDCl_3$

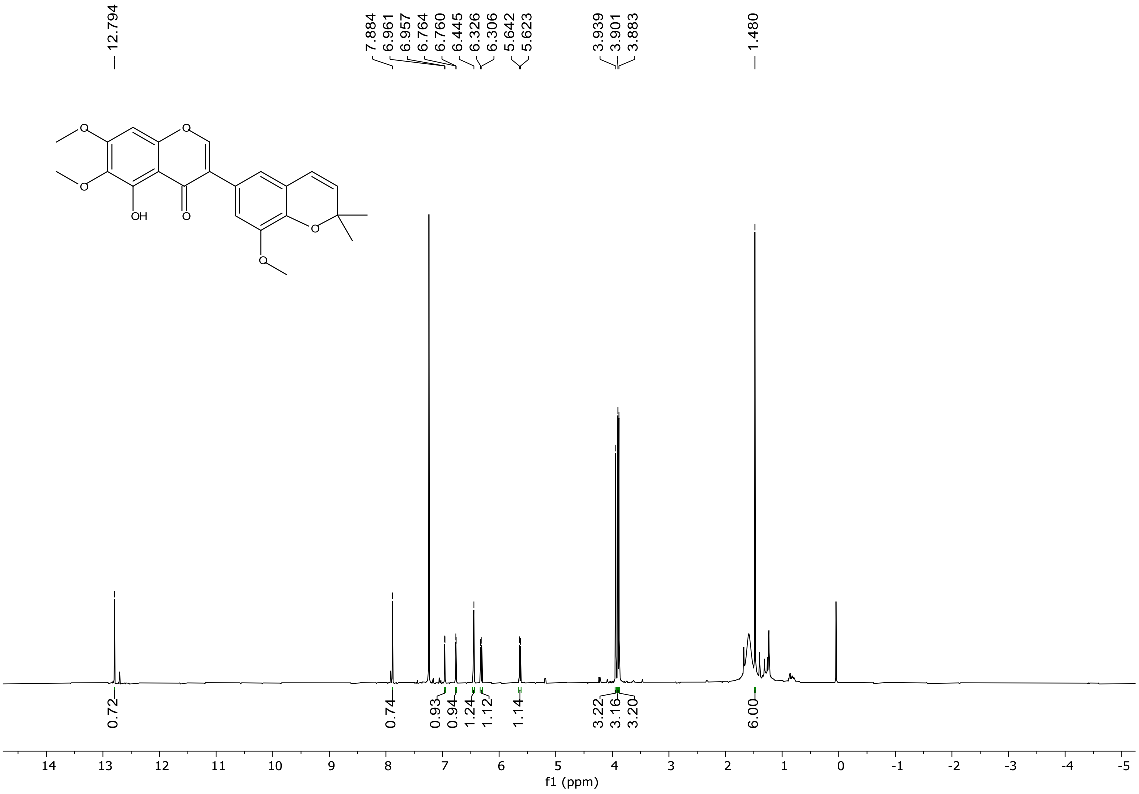

Figure S35. ^{1}H NMR spectrum of 4 at 500 MHz in $CDCl_3$ (expansion: 3.2 – 8.2 ppm)

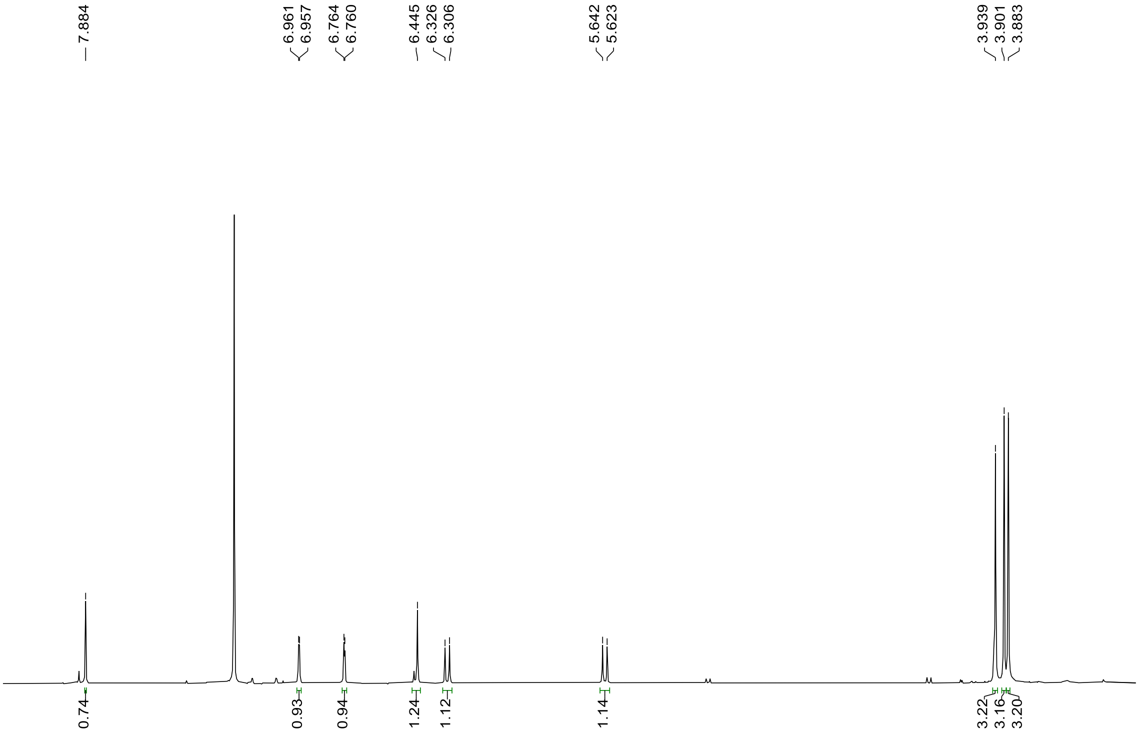

Figure S36. ^{13}C NMR spectrum of 4 at 500 MHz in CDCl$_3$

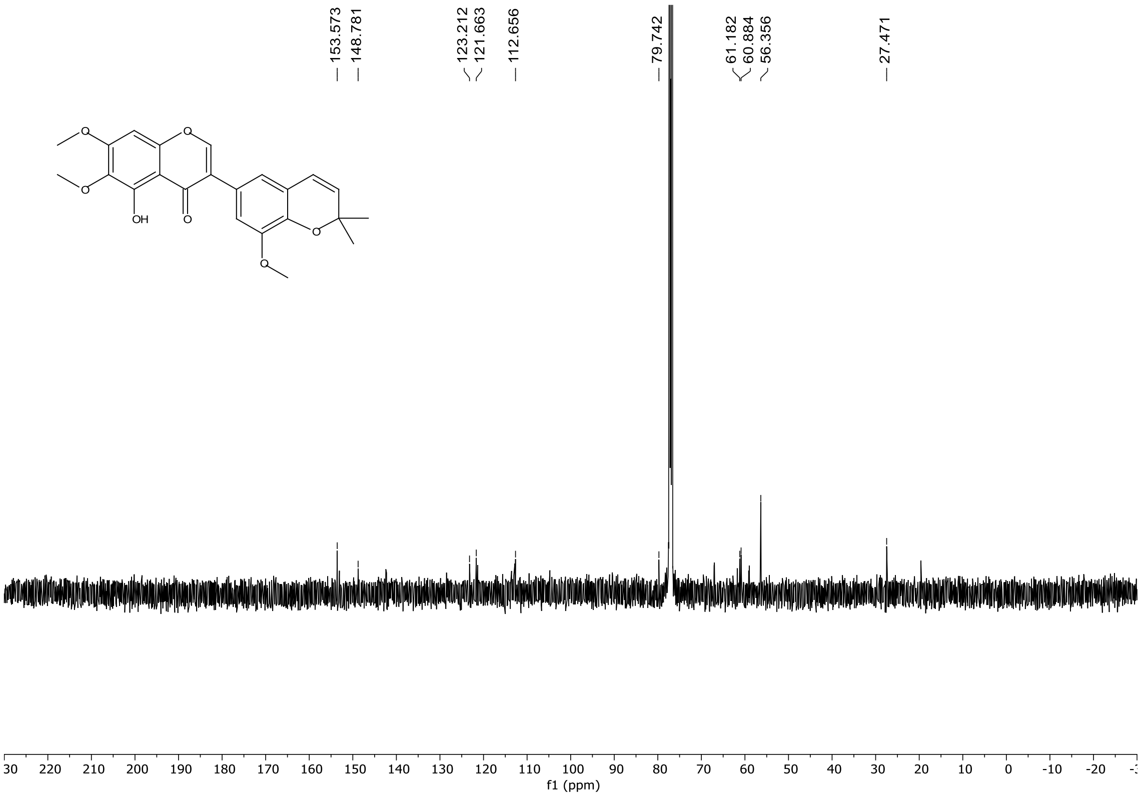

Figure S37. HSQC spectrum of 4 at 500 MHz in CDCl$_3$

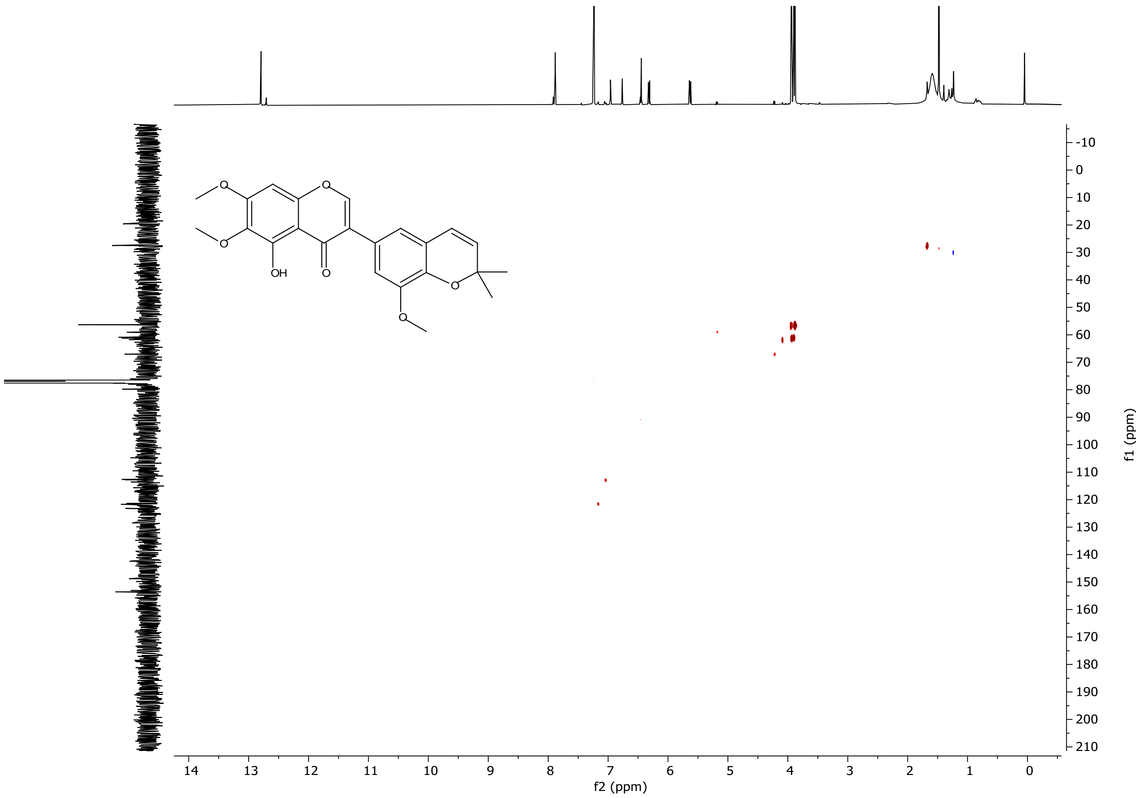

Figure S38. HMBC spectrum of 4 at 500 MHz in $CDCl_3$

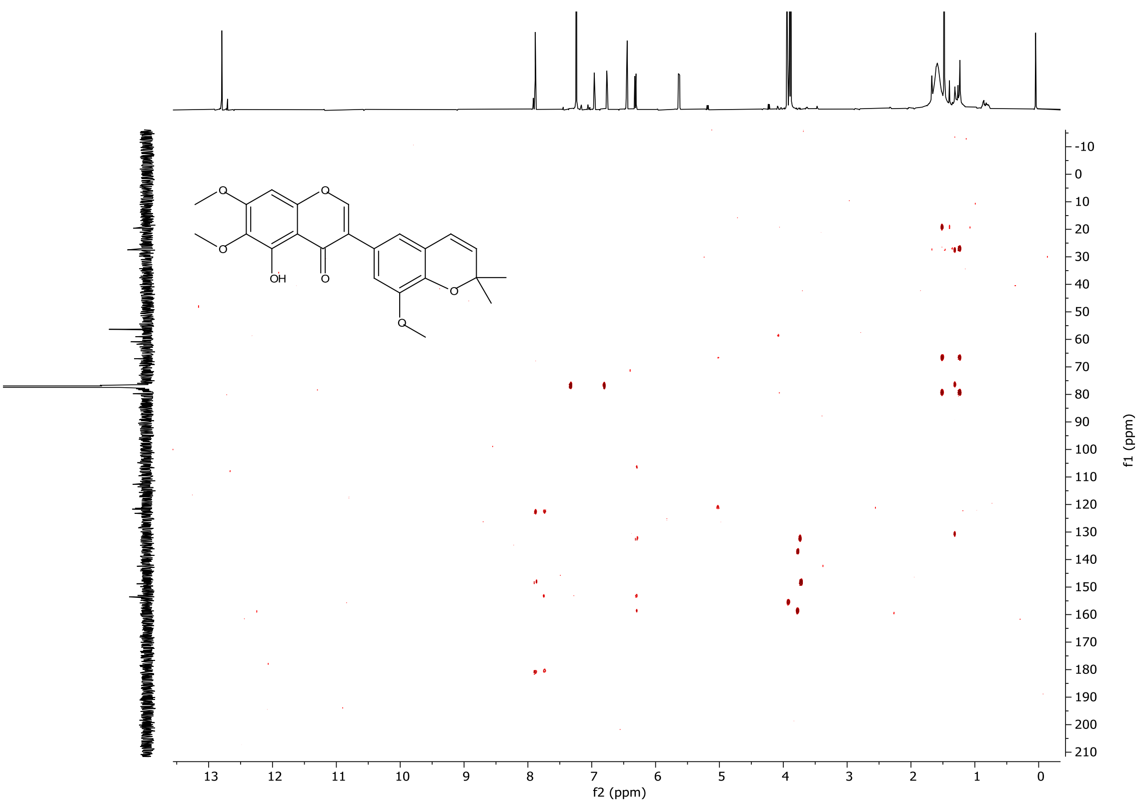

Figure S39. HMBC spectrum of 4 at 500 MHz in $CDCl_3$ (expansion:)

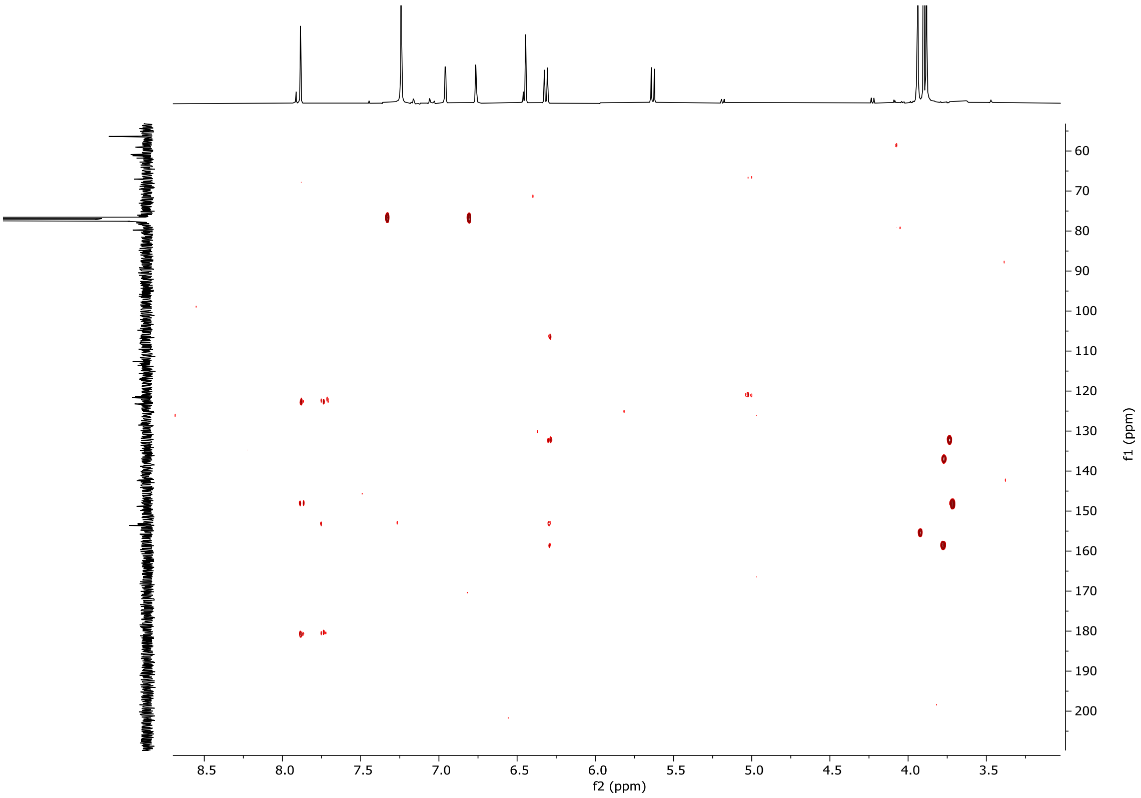

Figure S40. IR spectrum of 4 (liquid solution)

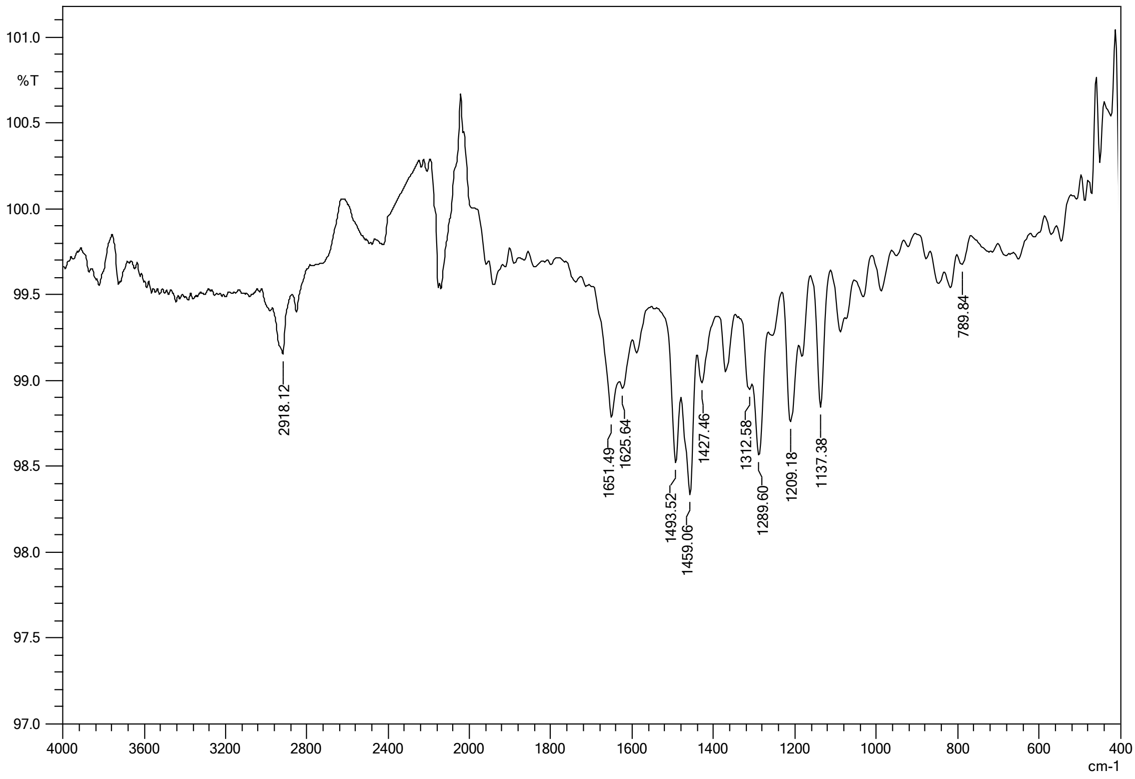

Scheme S5. Fragmentation proposal of compound 5 (m/z 369, rt: 36.4 min, cluster D)

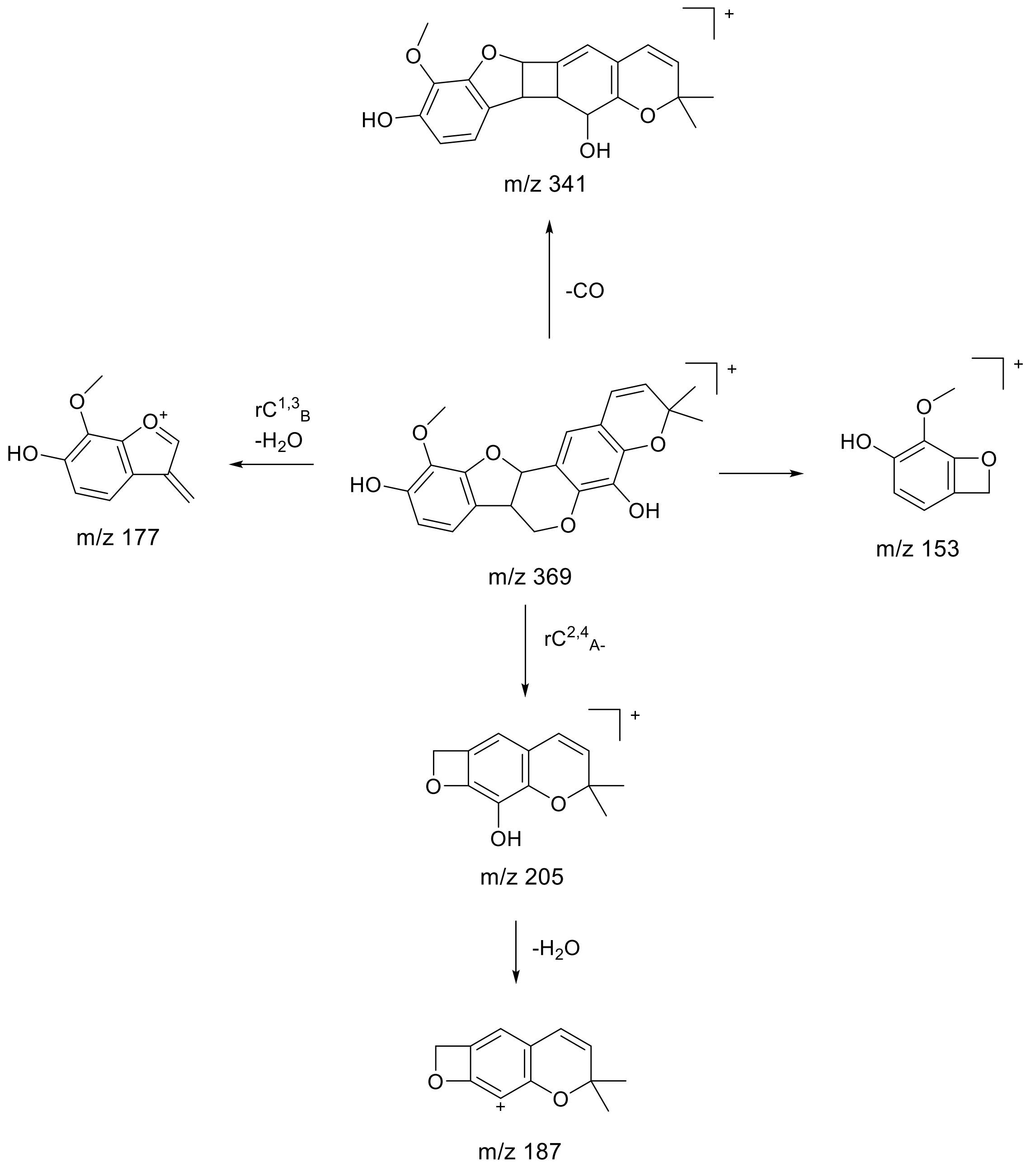

Figure S41. HRESIMS and LC-ESIMS/MS spectrum of 5 ([M + H] $^+$, positive mode)

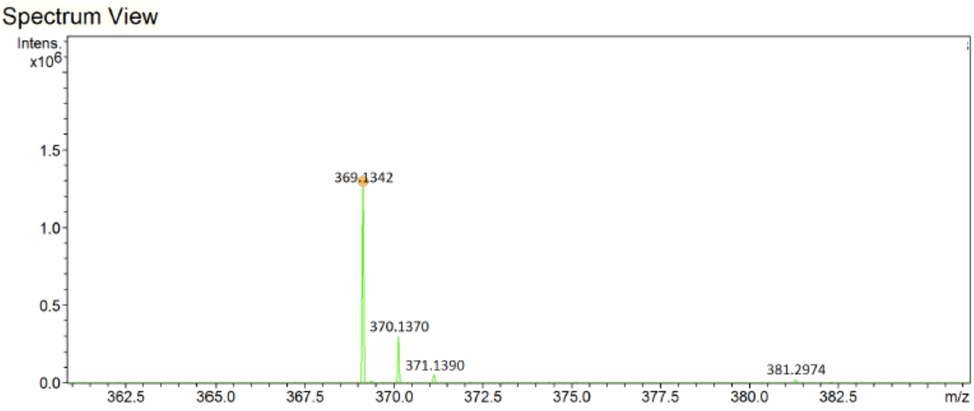

Meas. m/z	#	Ion Formula	m/z	err [ppm]	mSigma	# mSigma	Score	rdb	e¯ Conf	N-Rule
369.1342	1	C21H21O6	369.1333	-2.6	4.6	1	100.00	11.5	even	ok

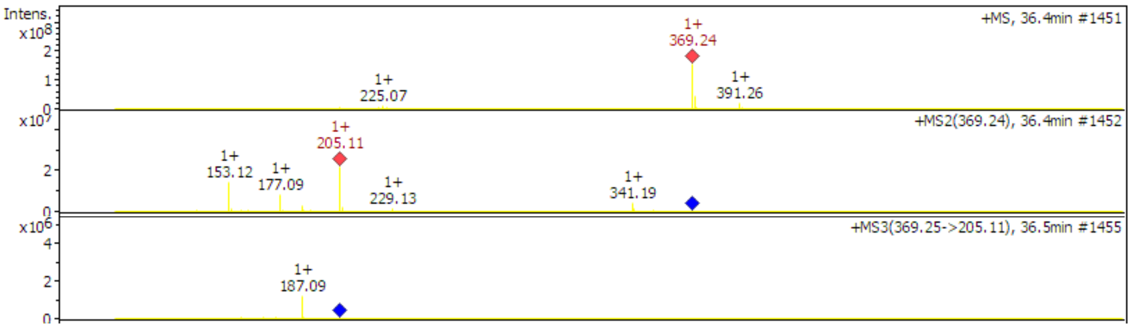

Figure S42. ^{1}H NMR spectrum of 5 at 400 MHz in $CDCl_3$

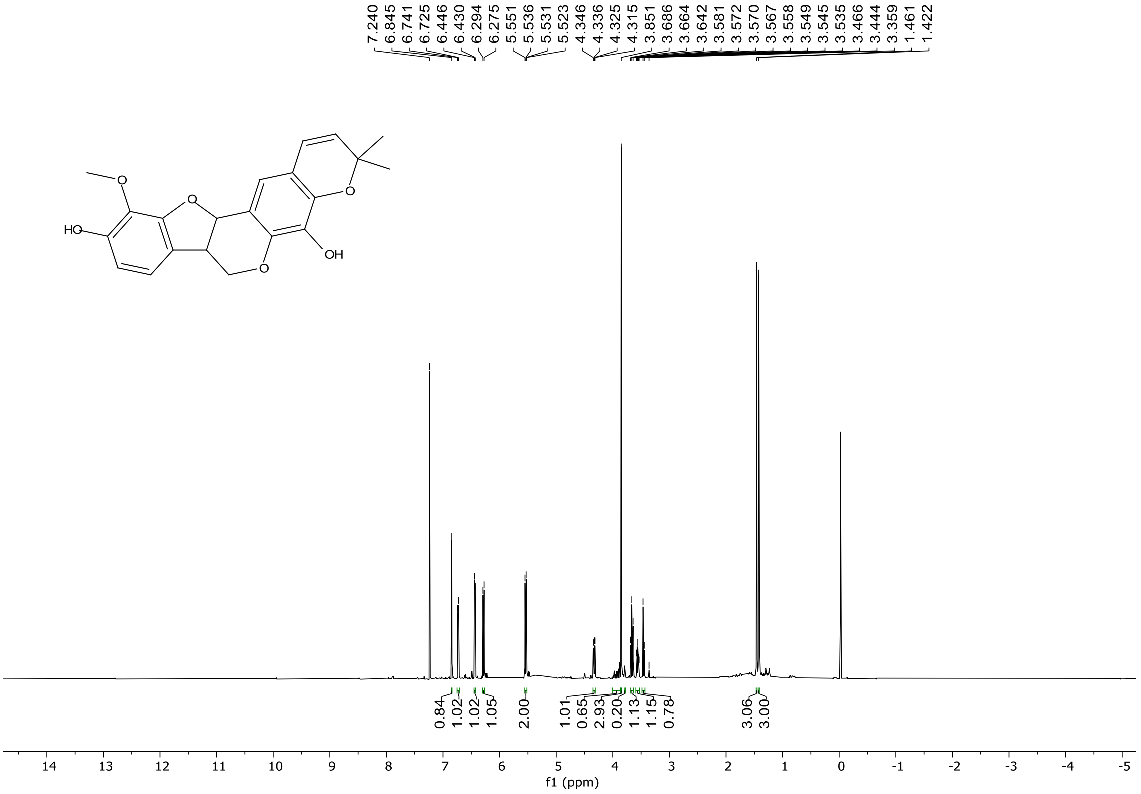

Figure S43. ^{1}H NMR spectrum of 5 at 400 MHz in $CDCl_3$ (expansion: 3.3 – 4.4 ppm)

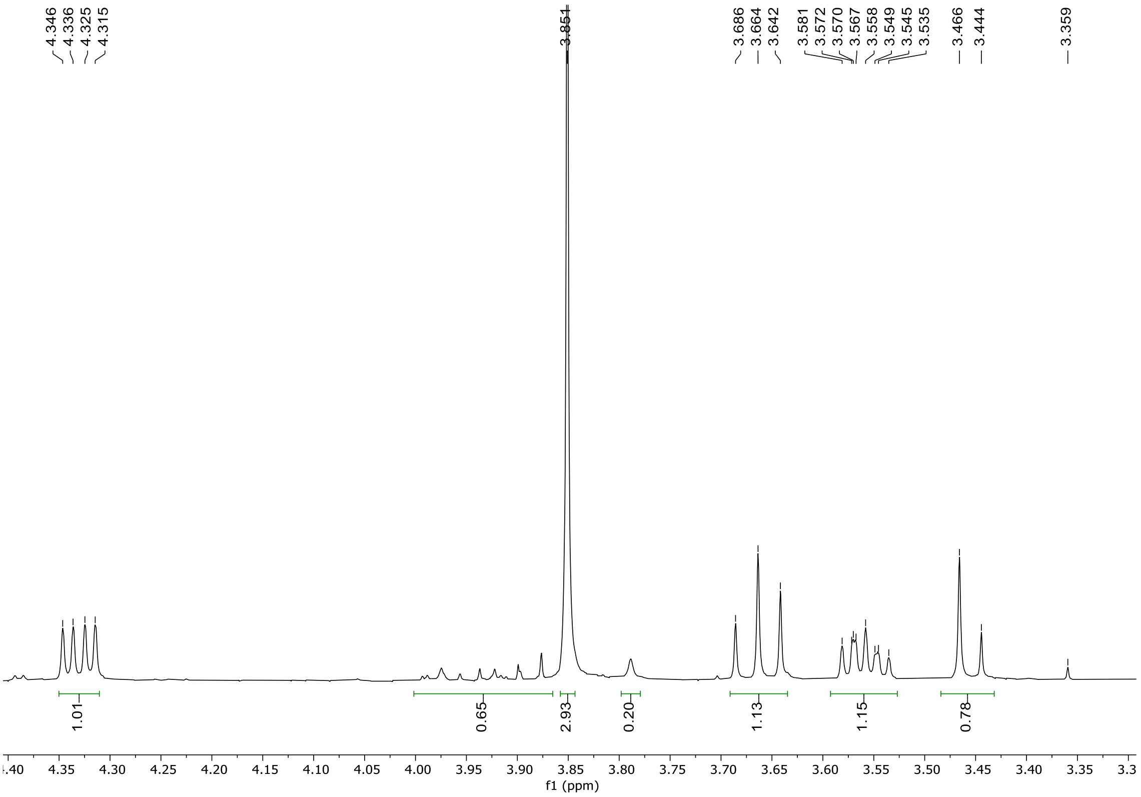

Figure S44. ^{1}H NMR spectrum of 5 at 400 MHz in CDCl$_3$ (expansion: 5.2 – 7.3 ppm)

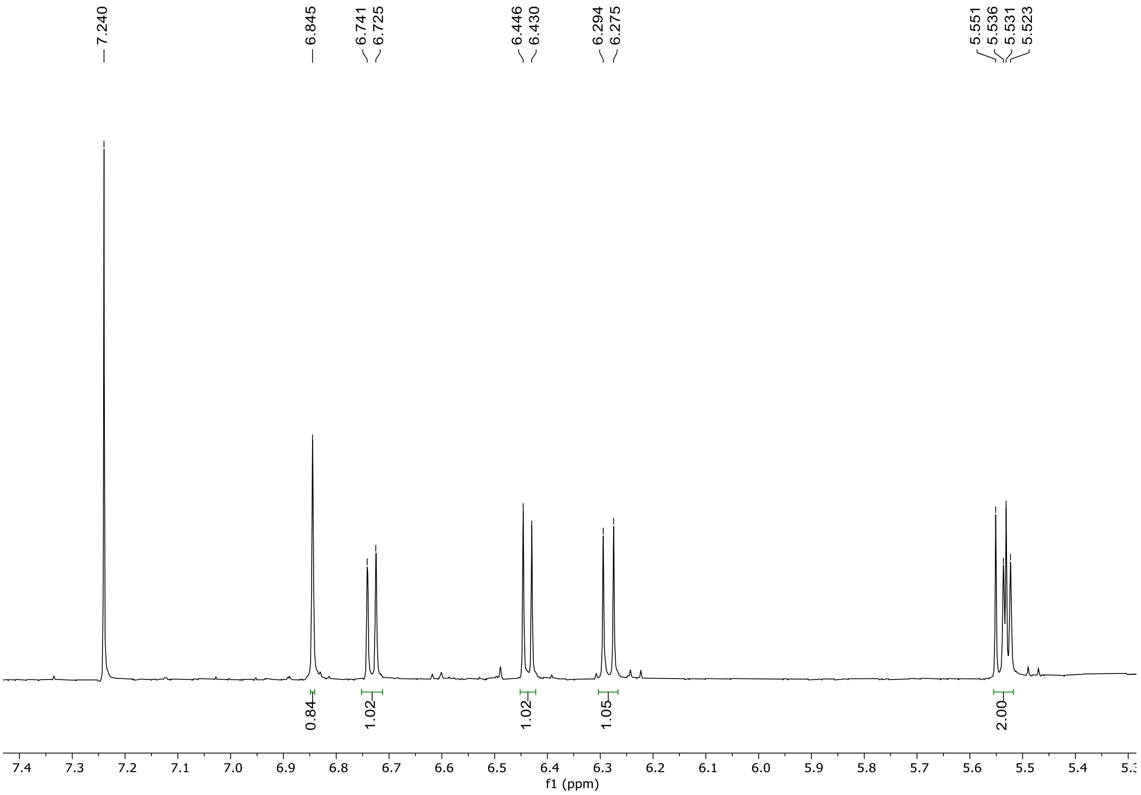

Figure S45. ^{13}C NMR spectrum of 5 at 500 MHz in CDCl$_3$

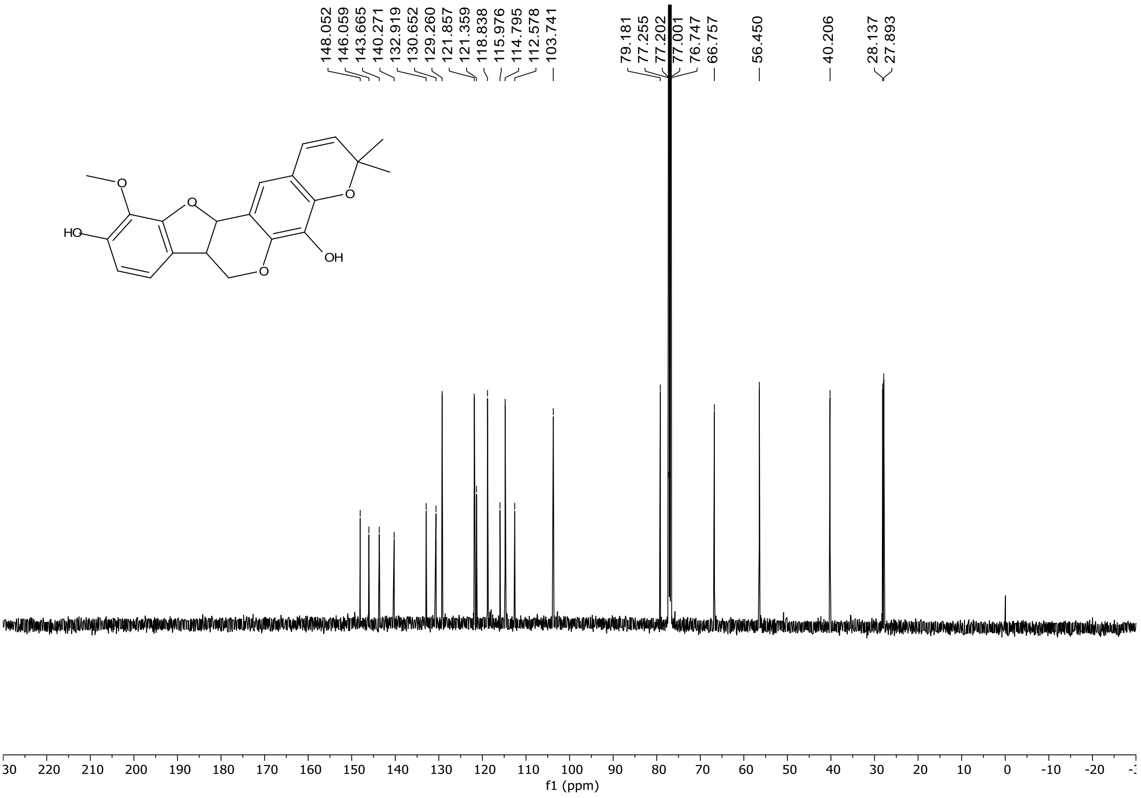

Figure S46. ^{13}C NMR spectrum of 5 at 500 MHz in $CDCl_3$ (expansion: 100 – 150 ppm)

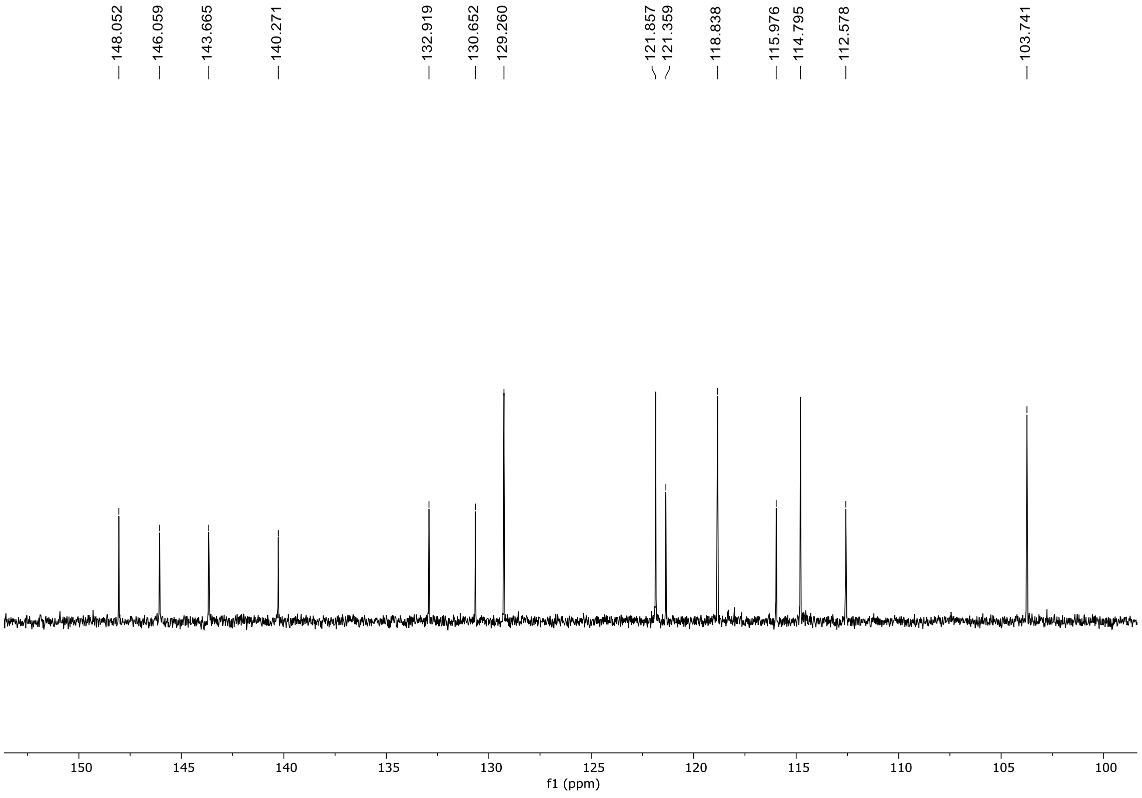

Figure S47. DEPT135 spectrum of 5 at 500 MHz in $CDCl_3$

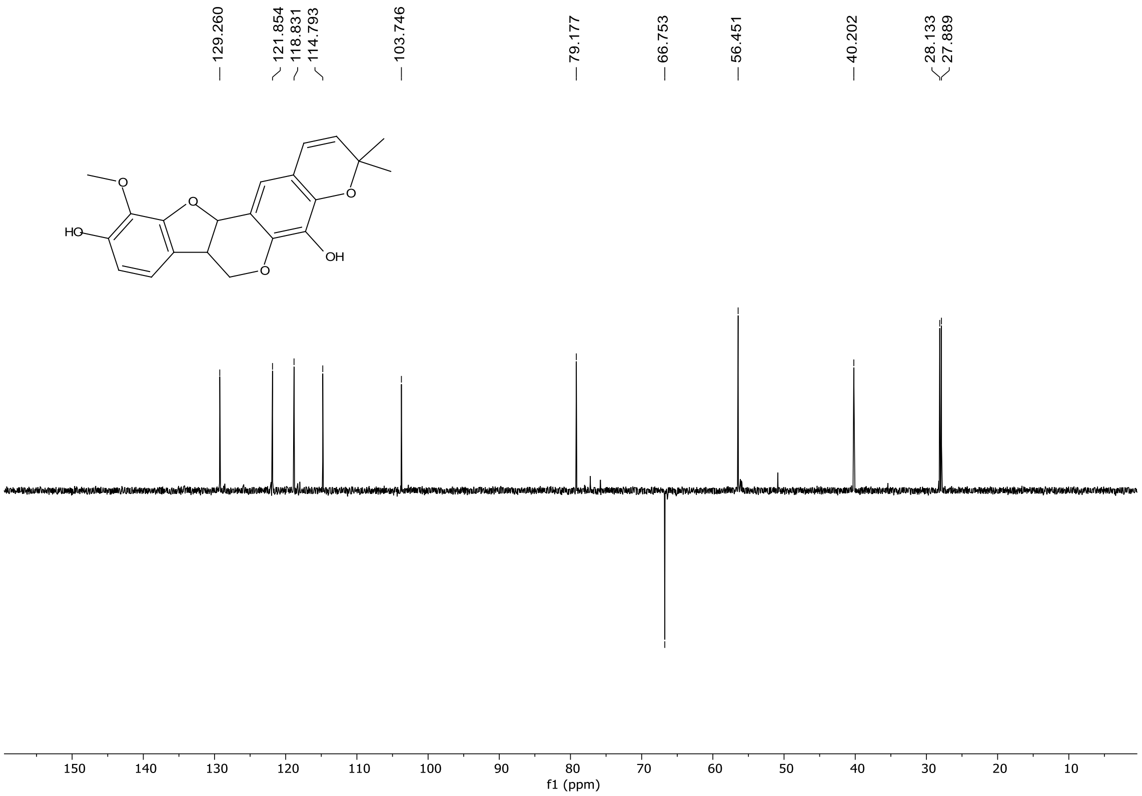

Figure S48. HSQC spectrum of 5 at 500 MHz in $CDCl_3$

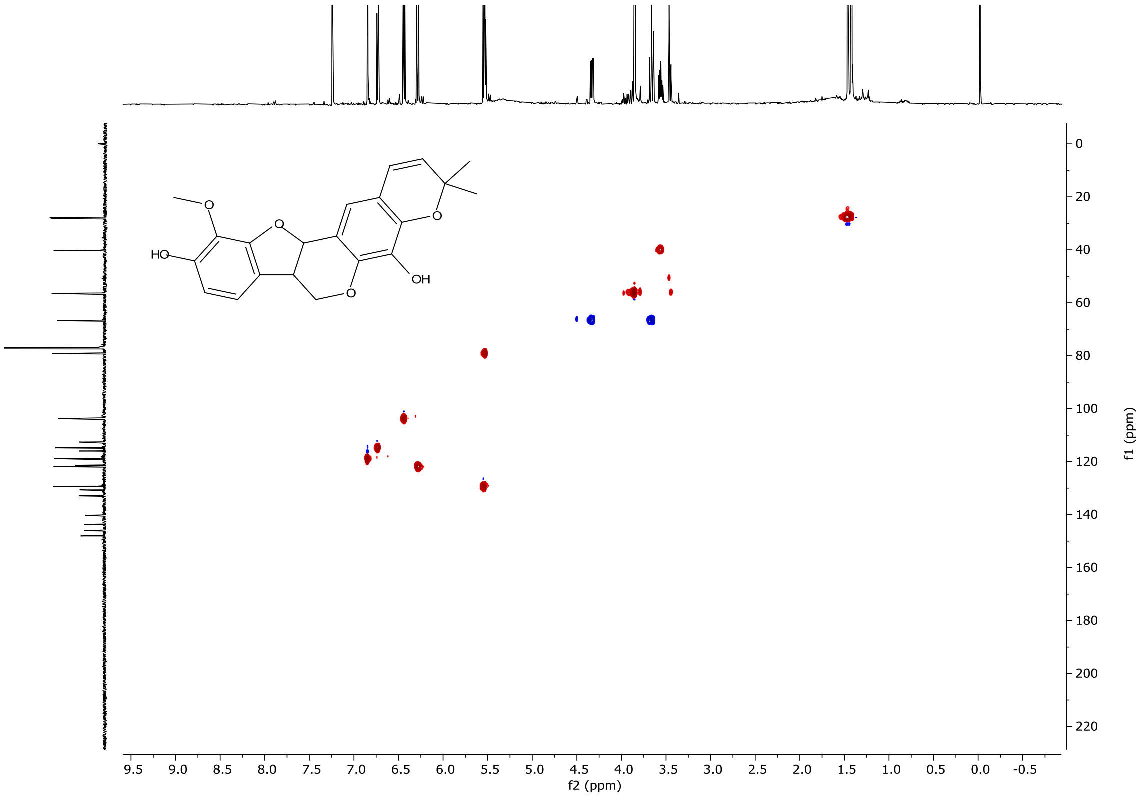

Figure S49. HMBC spectrum of 5 at 500 MHz in $CDCl_3$

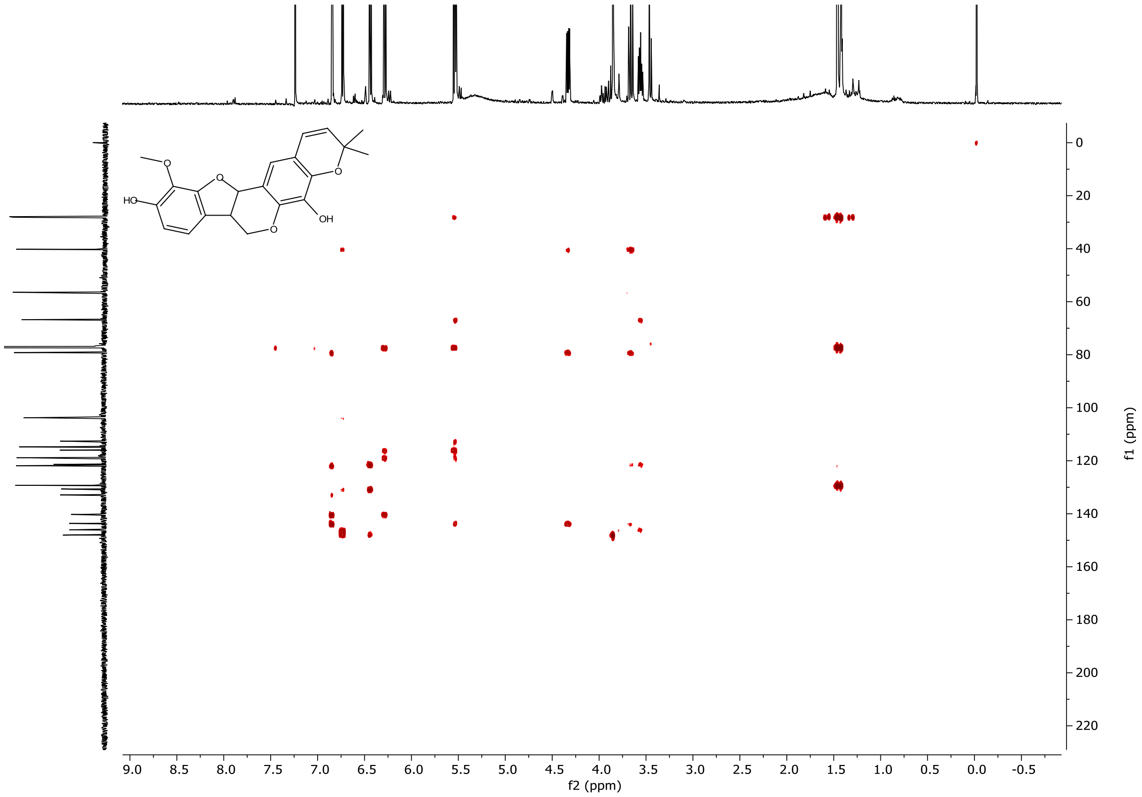

Figure S50. COSY spectrum of 5 at 500 MHz in $CDCl_3$

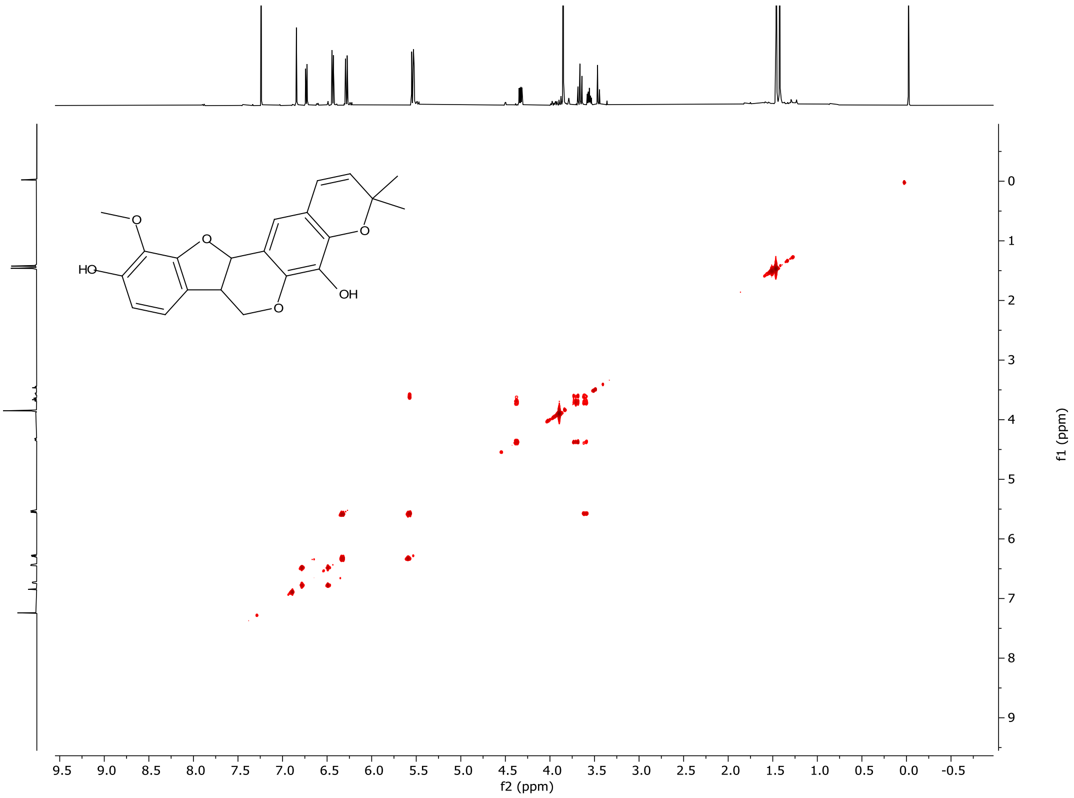

Figure S51. IR spectrum of 5 (liquid solution)

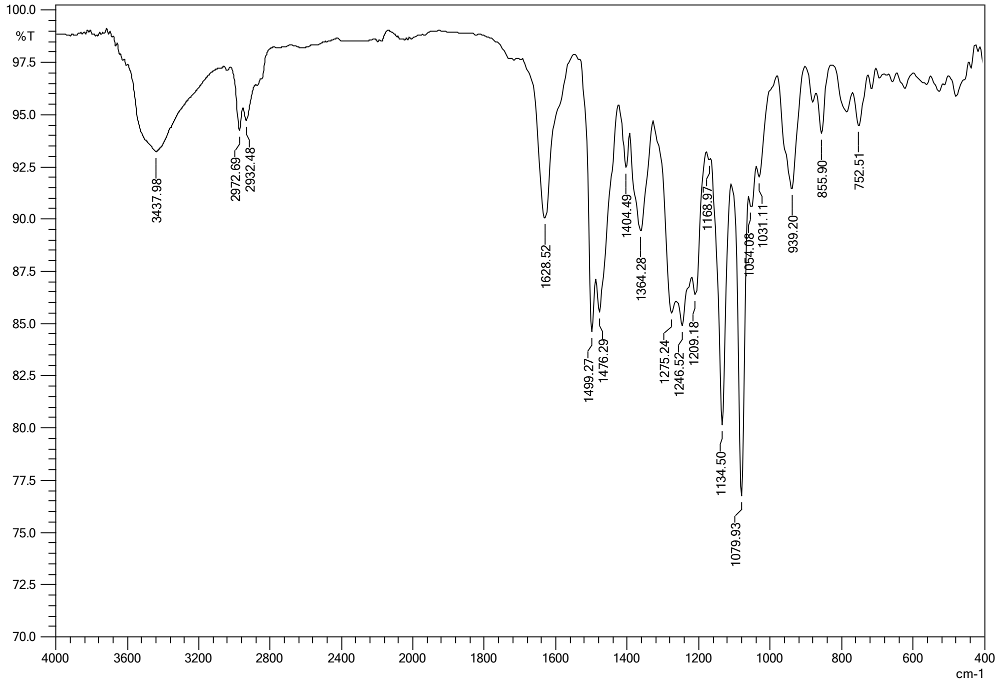

Scheme S6. Fragmentation proposal of compound 6 (m/z 383, rt: 42.7 min, cluster D)

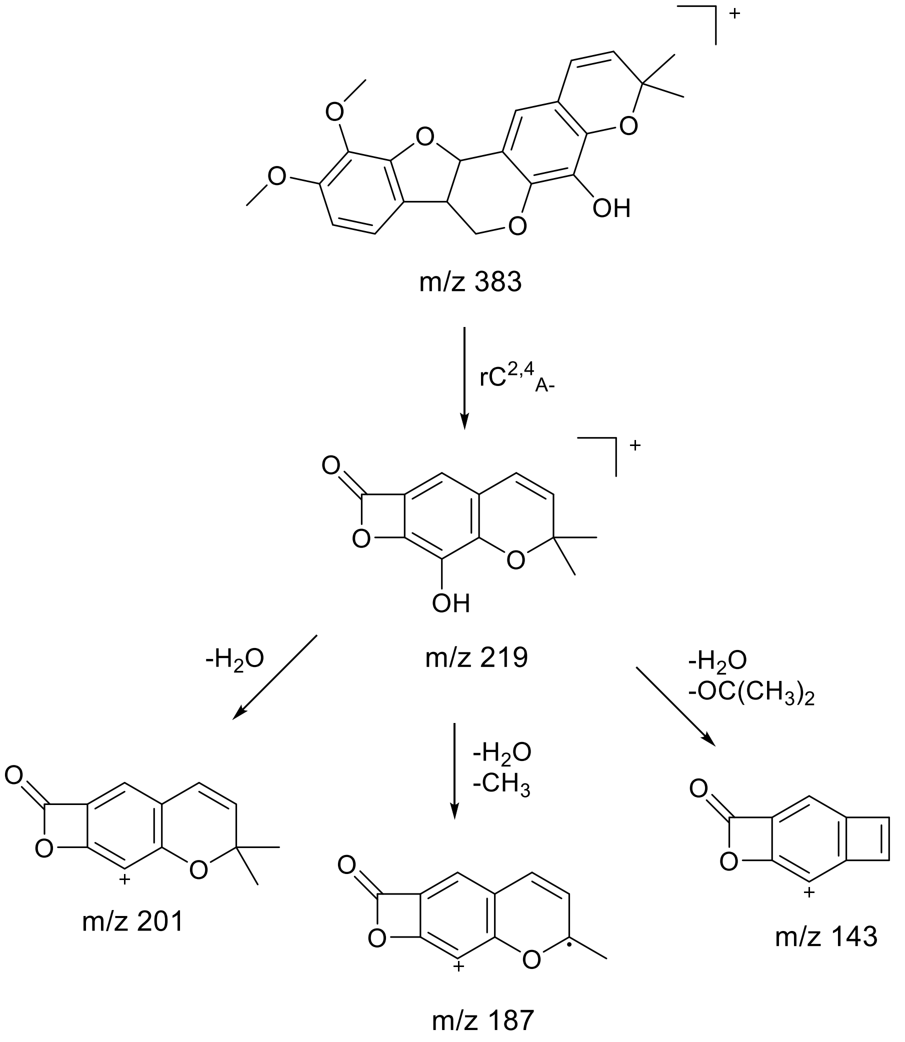

Figure S52. HRESIMS and LC-ESIMS/MS spectrum of 6 ([M + H]$^+$, positive mode)

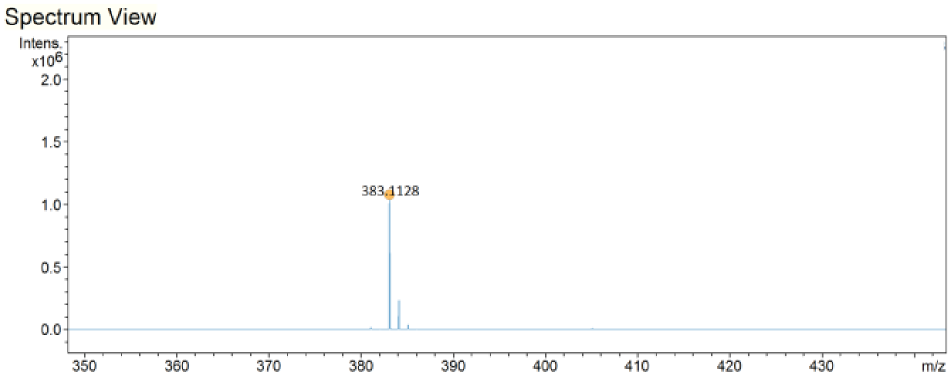

Meas. m/z	#	Ion Formula	m/z	err [ppm]	mSigma	# mSigma	Score	rdb	e⁻ Conf	N-Rule
383.1128	1	C22H23O6	383.1125	-0.6	2.0	1	100.00	12.5	even	ok

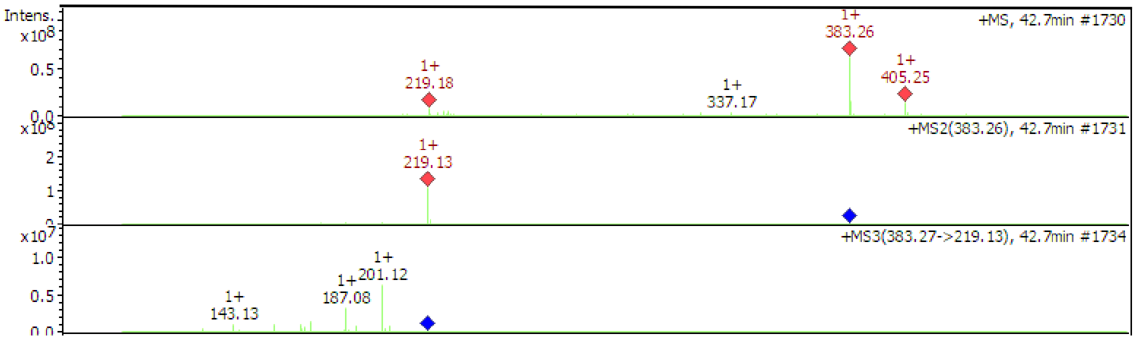

Figure S53. ^{1}H NMR spectrum of 6 at 400 MHz in $CDCl_3$

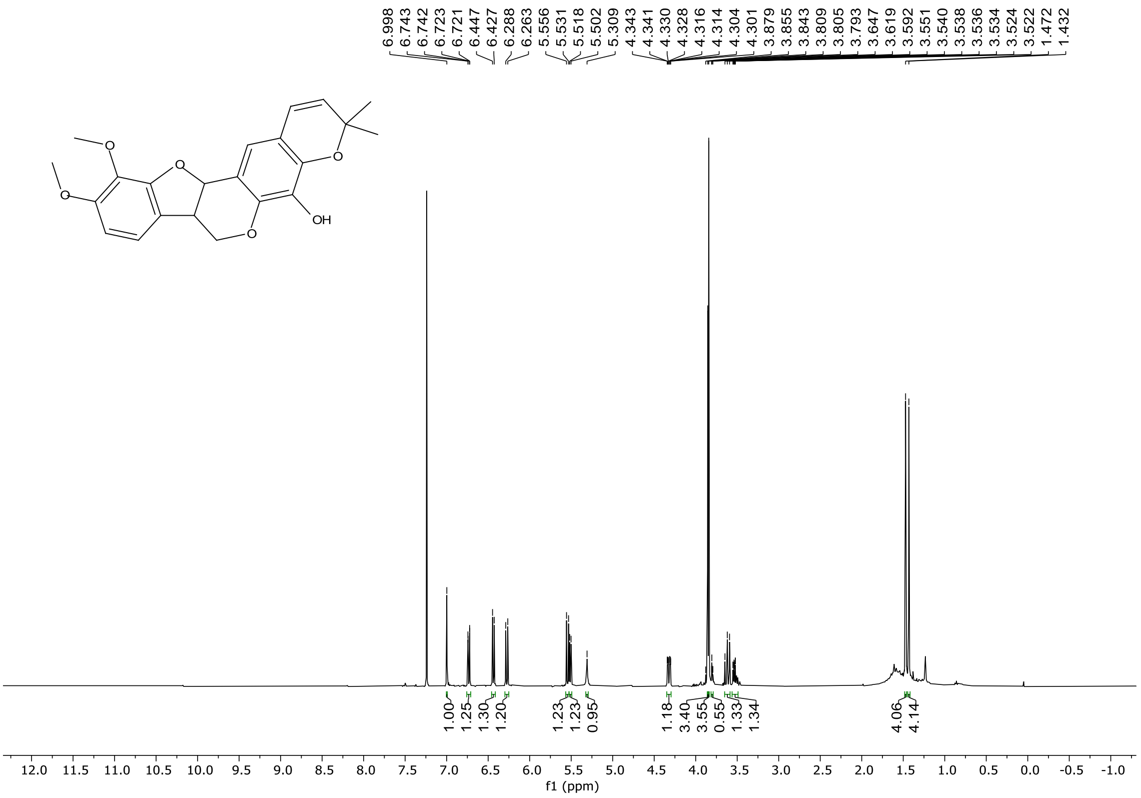

Figure S54. ^{1}H NMR spectrum of 6 at 400 MHz in $CDCl_3$ (expansion: 3.4 – 4.4 ppm)

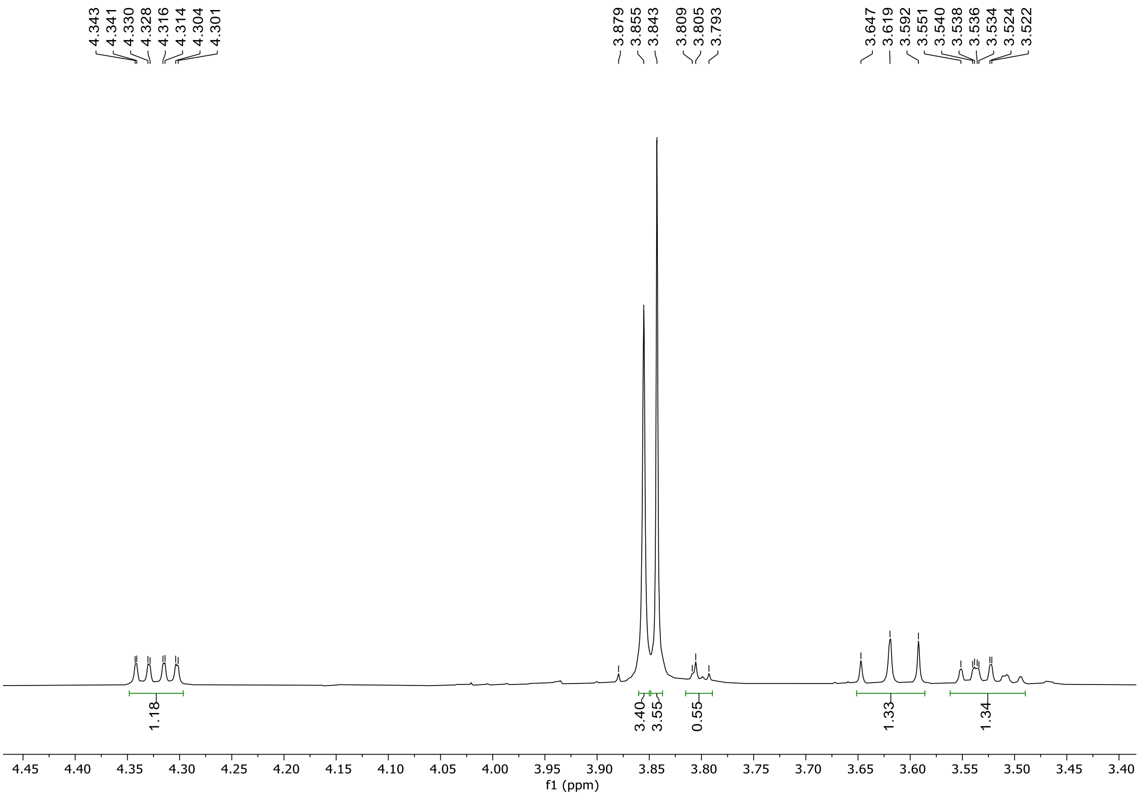

Figure S55. ^{1}H NMR spectrum of 6 at 400 MHz in $CDCl_3$ (expansion: 5.0 – 7.3 ppm)

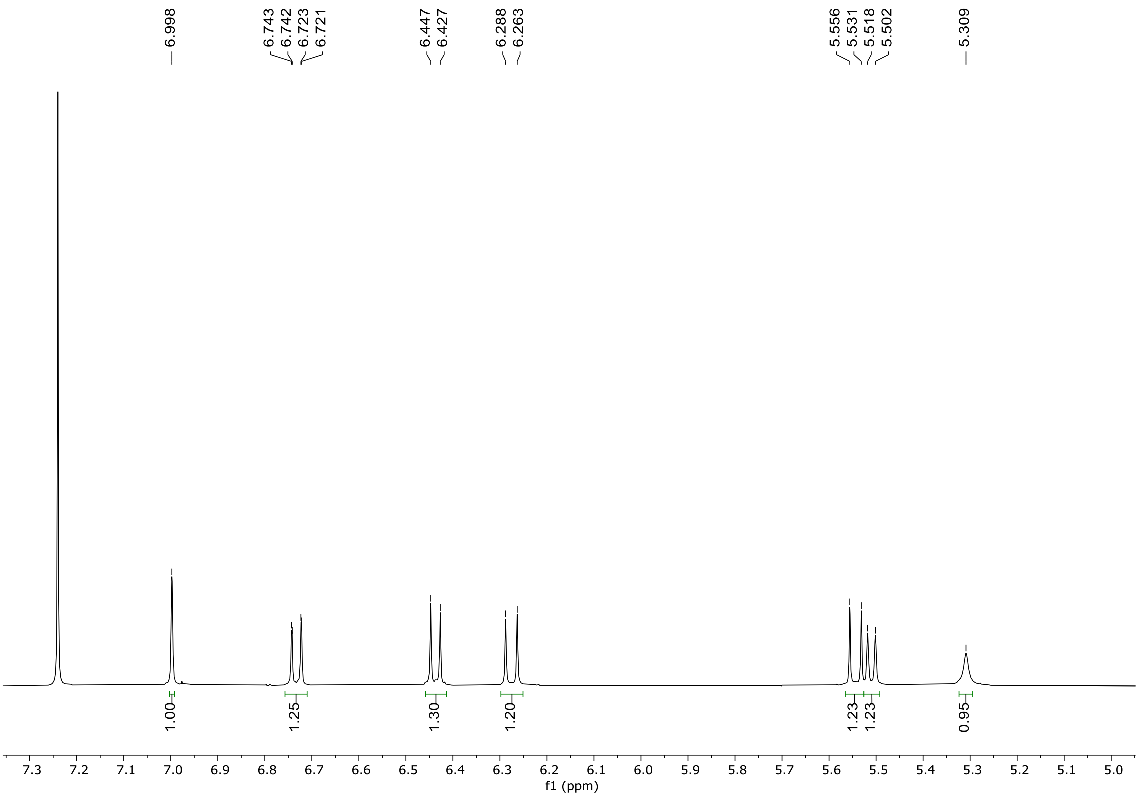

Figure S56. ^{13}C NMR spectrum of 6 at 100 MHz in $CDCl_3$

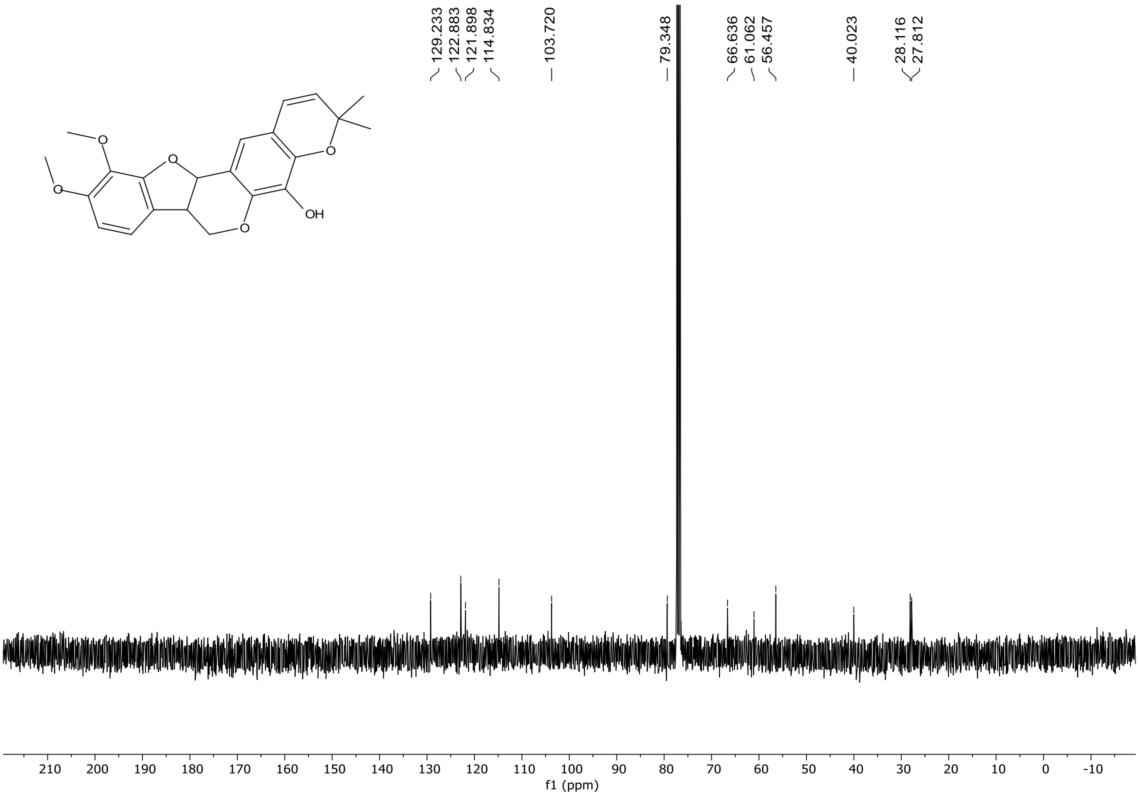

Figure S57. HSQC spectrum of 6 at 400 MHz in CDCl$_3$

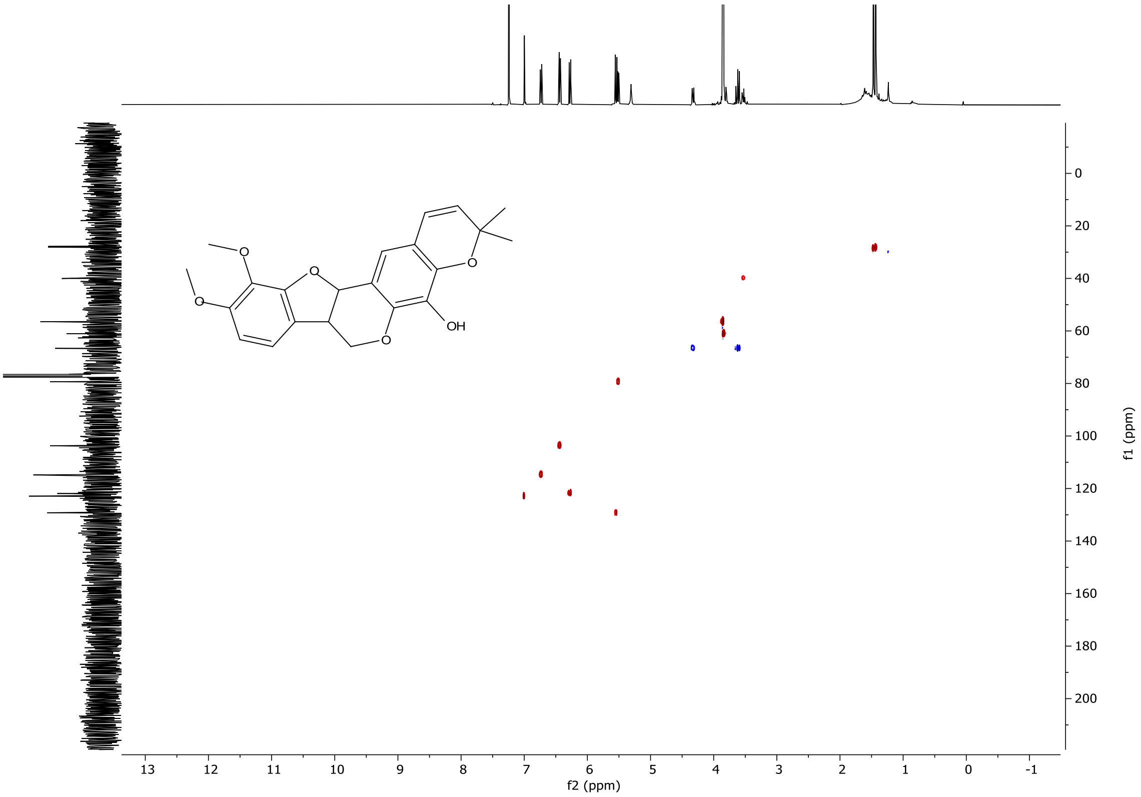

Figure S58. HMBC spectrum of 6 at 400 MHz in CDCl$_3$

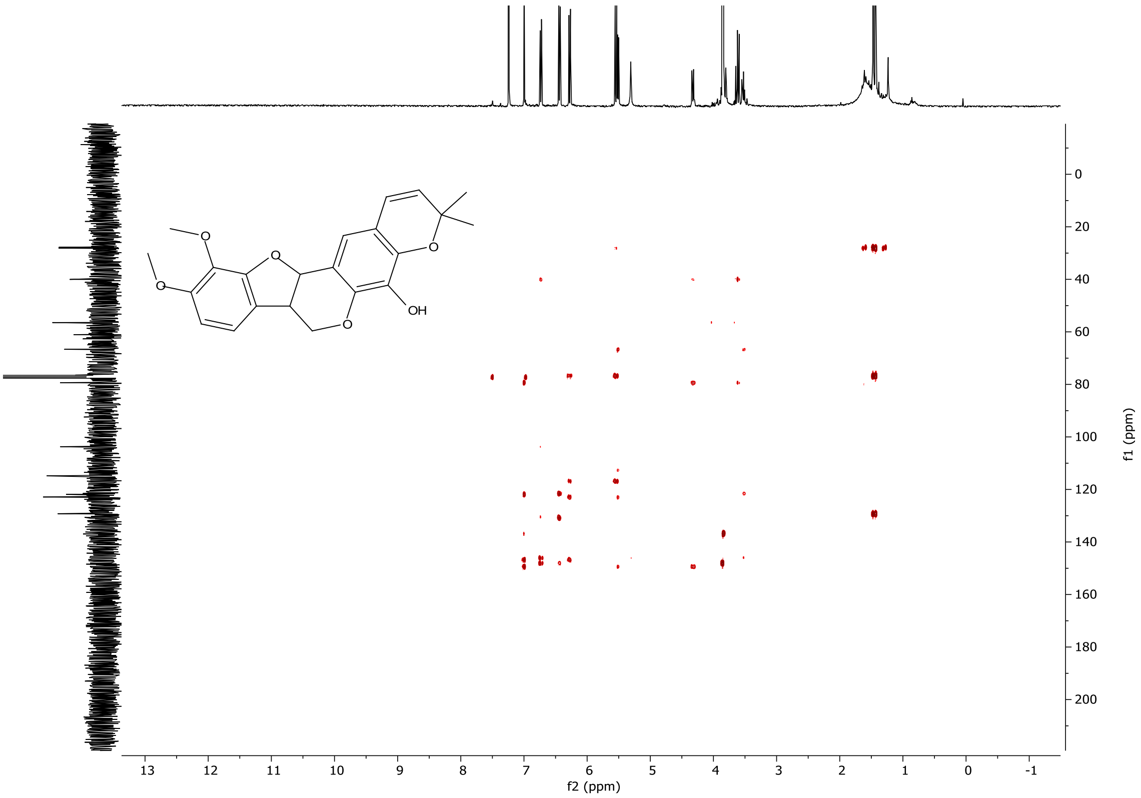

Figure S59. COSY spectrum of 6 at 400 MHz in $CDCl_3$

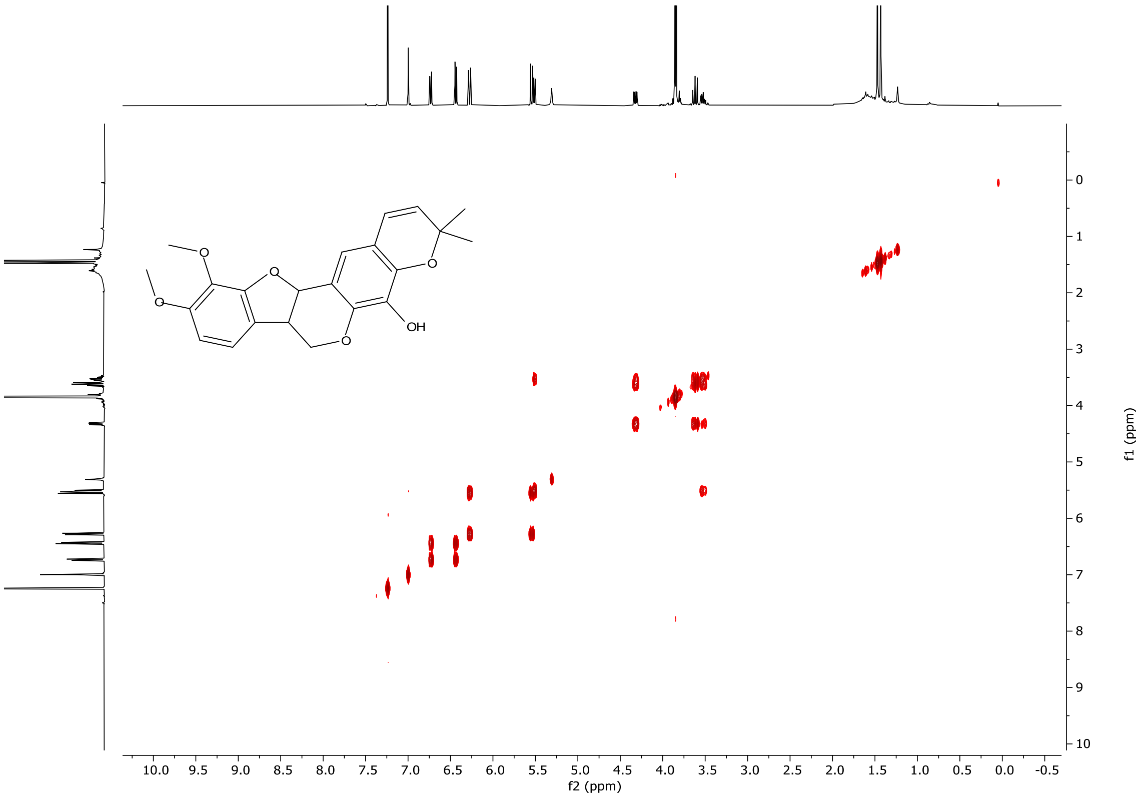

Figure S60. NOESY spectrum of 6 at 400 MHz in $CDCl_3$

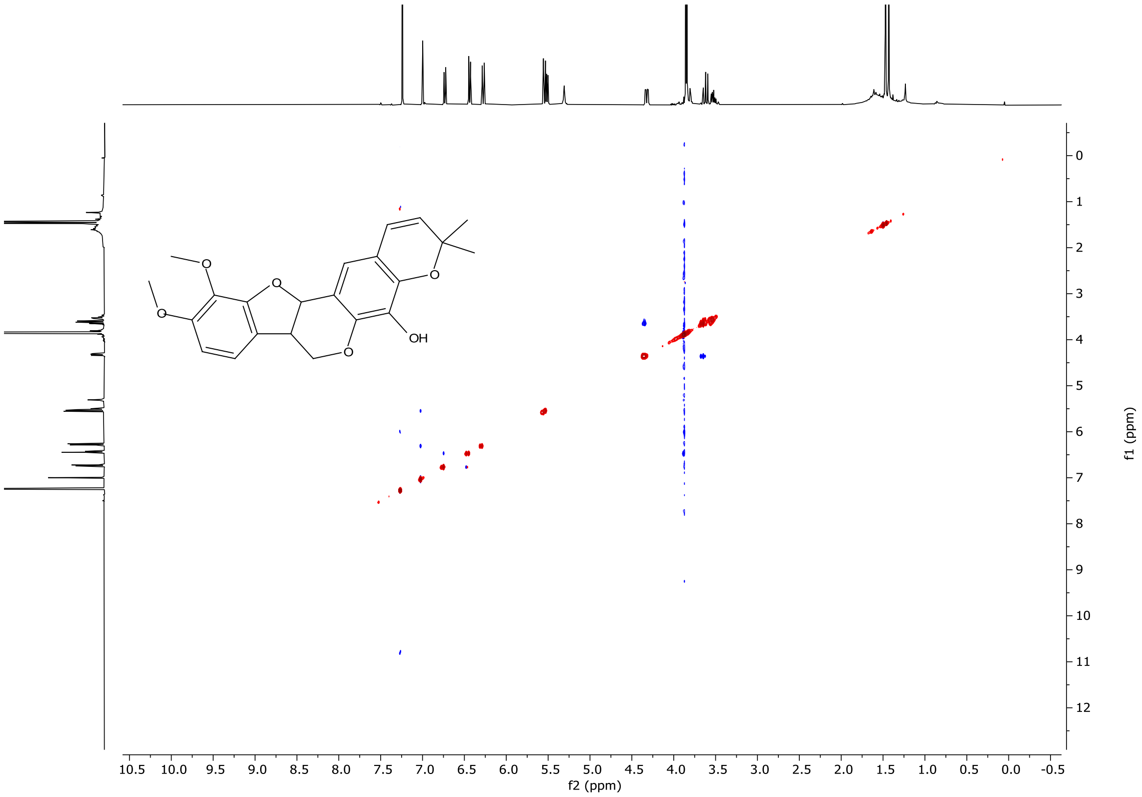

Table S2: LC-ESIMS^3 and LC-HRESIMS data of compounds

R.t.	*Meas.* m/z	m/z	Formula	Erro (ppm)	EM^n *m/z*	Composto	Cluster	Reference
24.0	331.1176	331	$C_{17}H_{15}O_7$	0.1	MS^2 [331]: 299 (100), 153 (40); MS^3 [331 → 299]: 239 (100)	3,7-Di-*O*-methylquercetin	E	-
27.0	389.1243	389	$C_{20}H_{21}O_8$	-3.2	MS^2 [389]: 374 (100), 356 (78), 328 (20), 300 (3), 297 (3), 285 (2), 268 (1), 237 (0.6), 211 (8), 181 (1.6), 111 (0.8); MS^3 [389 → 374]: 356 (100), 328 (15), 313 (3), 269 (1), 211 (9.9)	Umuhengerin	C	Zhang et al, 2011
28.4	375.1448	375	$C_{20}H_{23}O_7$	-2.7	MS^2 [375]: 343 (100), 315 (16), 221 (23); MS^3 [375 → 343]: 329 (100), 315 (98), 311 (55), 283 (27), 251 (9.7), 191 (13), 161 (20)	5,7,3',4',5'-pentamethoxy-flavanone	E	Zhang et al, 2011
36.4	369.1353	369	$C_{21}H_{21}O_6$	-5.6	MS^2 [369]: 229 (5.8), 205 (100), 177 (47), 153 (58); MS^3 [369 → 205]: 187 (100), 159 (14)	5	D	-
42.7	383.1510	383	$C_{22}H_{23}O_6$	-5.3	MS^2 [383]: 219 (100); MS^3 [383 → 219]: 201 (100), 187 (48), 143 (15)	6	D	-
55.3	355.2148	355	$C_{20}H_{19}O_6$	-2.1	MS^2 [355]: 339 (10), 285 (100), 269 (87); MS^3 [355 → 285]: 269 (100)	6-Prenylorobol (7) *	B	-

*** Confirmed by internal standard**

6-prenylorobol (**7**) - ^{1}H NMR (CD_3OD, 400 MHz) δ_H 7.99 (s, H-2), δ_H 7.01 (d, 2.0Hz, H-2'), δ_H 6.83 (m, 2H, H-5', H-6'), δ_H 6.37 (s, 1H, H-8), δ_H 5.23 (m, H-2''), δ_H 3.30 (d, 6.5Hz, H-1''), δ_H 1.77 (s, 3H, Me-5), δ_H 1.66 (s, 3H, Me-6). ^{13}C NMR (CD_3OD, 100 MHz) δ_C 17.89 (Me-6), δ_C 22.27 (C1''), δ_C 25.93 (Me-5), δ_C 93.91 (C8), δ_C 106.08 (C10), δ_C 113.10 (C6), δ_C 116.37 (C5'), δ_C 117.50 (C2'), δ_C 121.76 (C6'), δ_C 123.35 (C2''), δ_C 124.03 (C3), δ_C 124.66 (C1''), δ_C 132.12 (C3''), δ_C 146.17 (C3'), δ_C 146.70 (C4'), δ_C 154.55 (C2), δ_C 157. 48 (C9), δ_C 160.45 (C5), δ_C 163.63 (C7), δ_C 182.25 (C=O).

3-*O*-metilquercetin (**8**) - ^{1}H NMR (CD_3OD, 400 MHz) δ_H 7.63 (d, 2.0Hz, H6'), δ_H 7.54 (dd, 8.5, 2.0Hz, H2'), δ_H 6.92 (d, 8.5Hz, H3'), δ_H 6.40 (d, 2.0Hz, H8), δ_H 6.21 (d, 2.0Hz, H6), δ_H 3.79 (s, 3H). ^{13}C NMR (CD_3OD, 100 MHz) δ_C 59.14 (Me-3), δ_C 93.36 (C8), δ_C 98.41 (C6), δ_C 104.45 (C10), δ_C 115.04 (C6), δ_C 120.94 (C2'), δ_C 121.53 (C1'), δ_C 138.13 (C3), δ_C 145.06 (C4'), δ_C 148.55 (C5'), δ_C 156.60 (C2), δ_C 157,00 (C9), δ_C 161.4 (C5), δ_C 164,5 (C7), δ_C 178.60 (C=O)

Figure S61. HRESIMS spectrum of 9 ([M + H] +, positive mode)

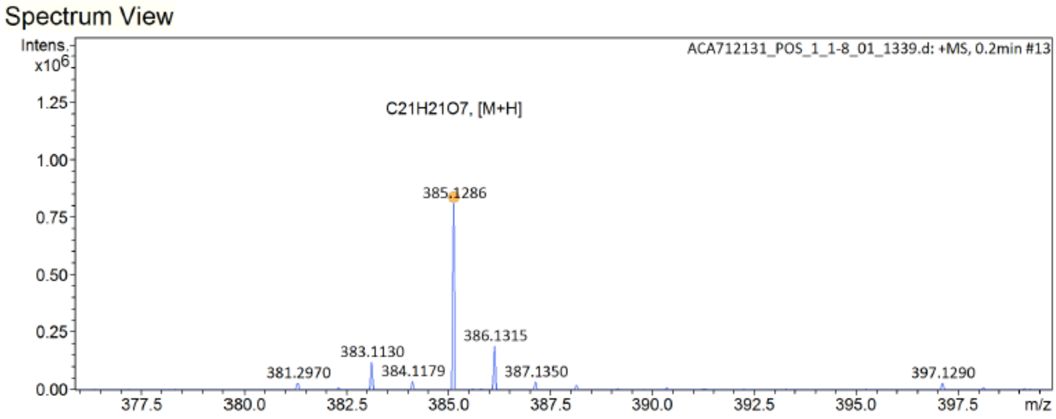

Meas. m/z	#	Ion Formula	m/z	err [ppm]	mSigma	# mSigma	Score	rdb	e¯ Conf	N-Rule
385.1286	1	C21H21O7	385.1282	-1.0	1.2	1	100.00	11.5	even	ok

Figure S62. ^{1}H NMR spectrum of 9 at 500 MHz in MeOD

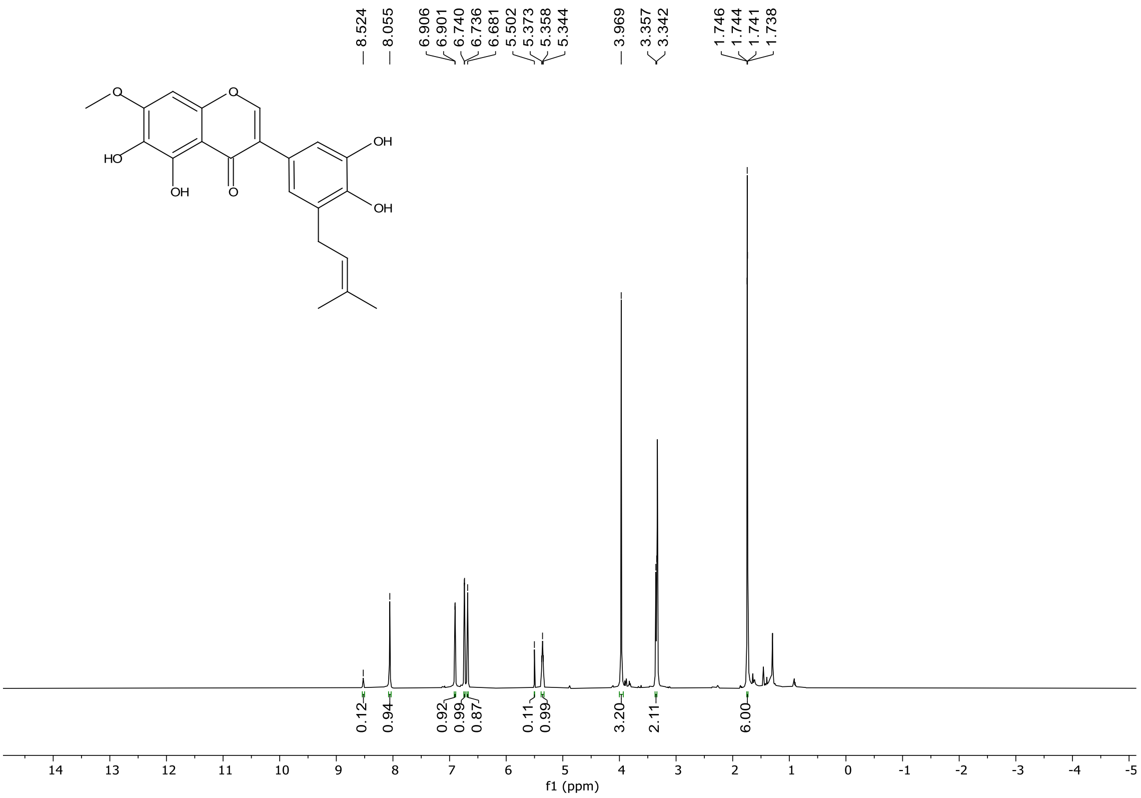

Figure S63. ^{1}H NMR spectrum of 9 at 500 MHz in MeOD (expansion: 5.1 – 8.7 ppm)

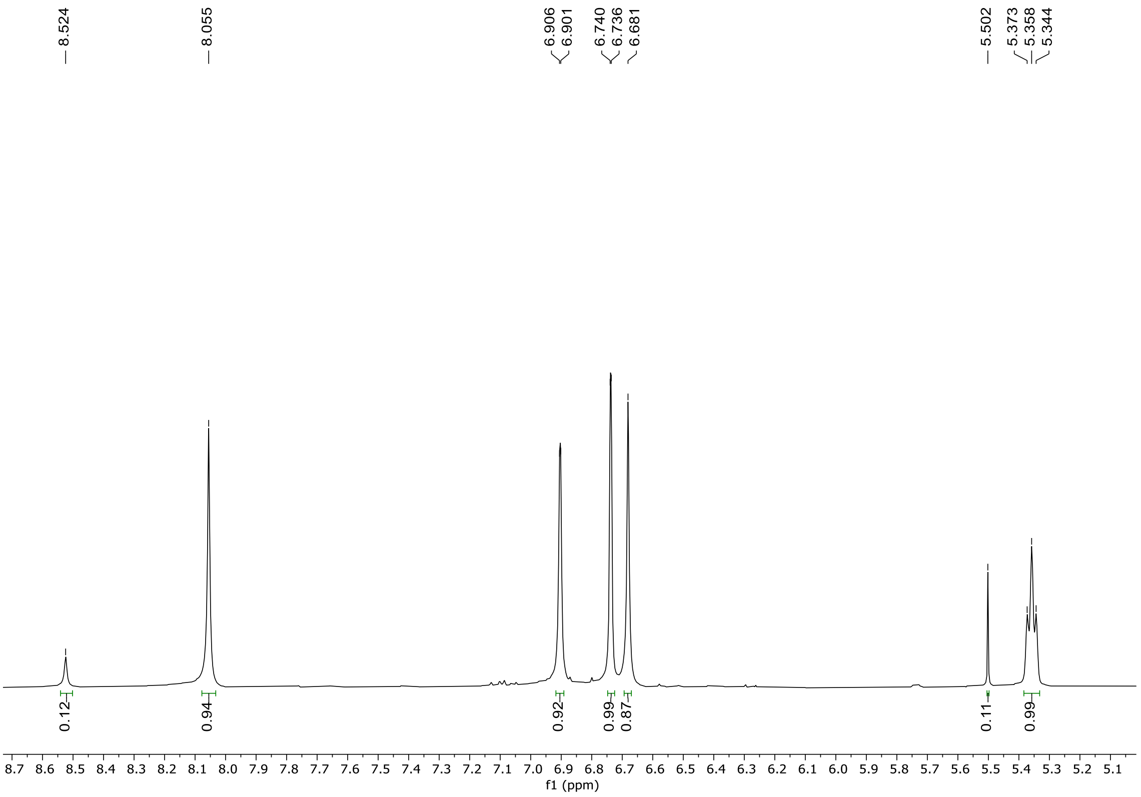

Figure S64. ^{13}C NMR spectrum of 9 at 125 MHz in MeOD

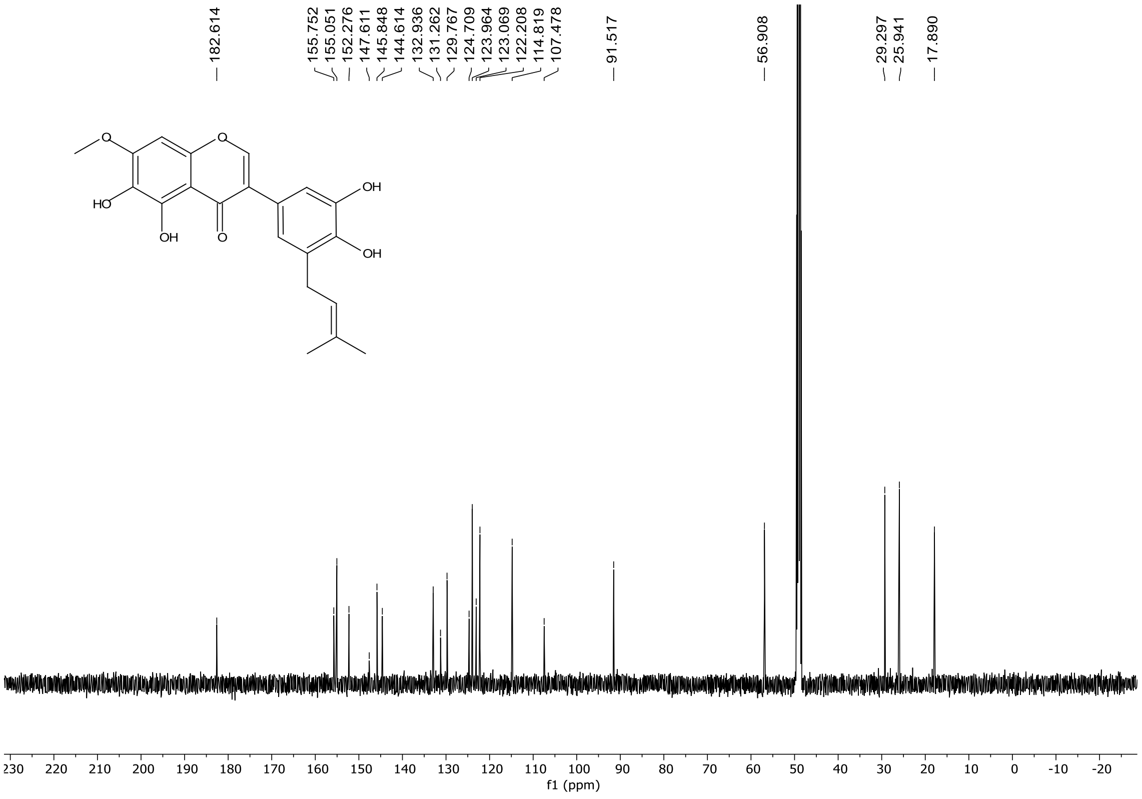

Figure S65. ^{13}C NMR spectrum of 9 at 125 MHz in MeOD (expansion: 90 – 160 ppm)

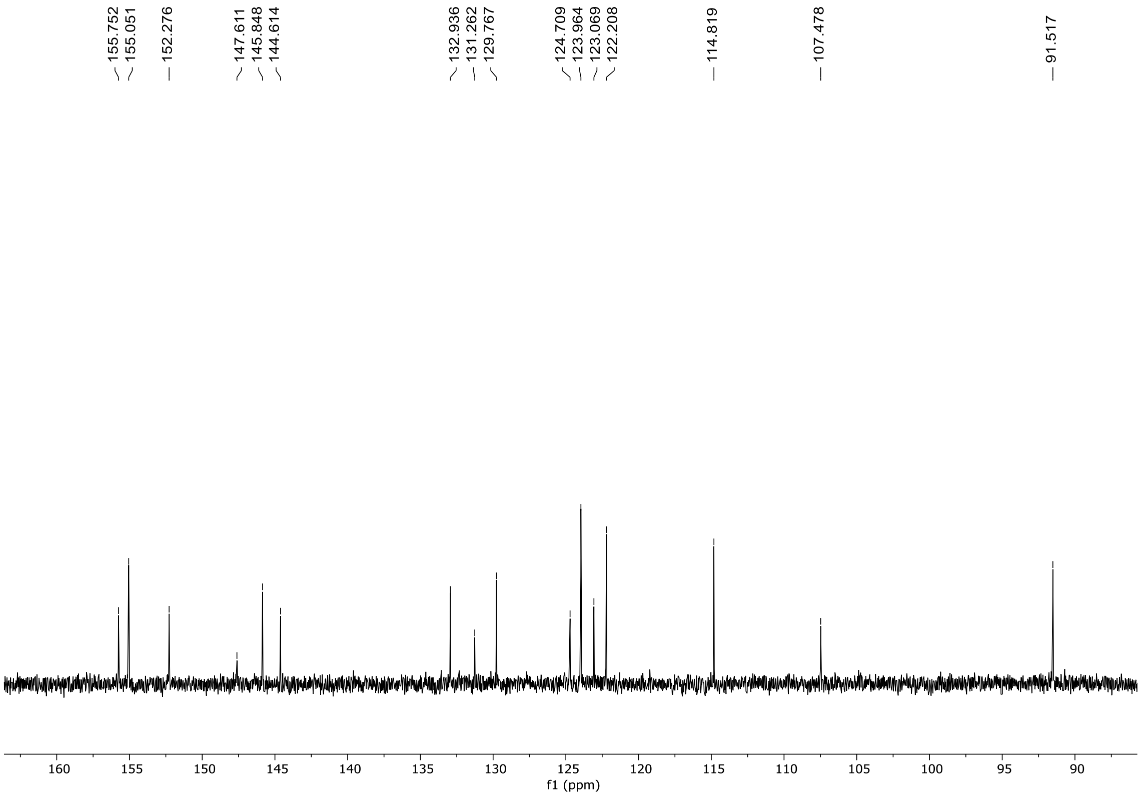

Figure S66. DEPT135 spectrum of 9 at 125 MHz in MeOD

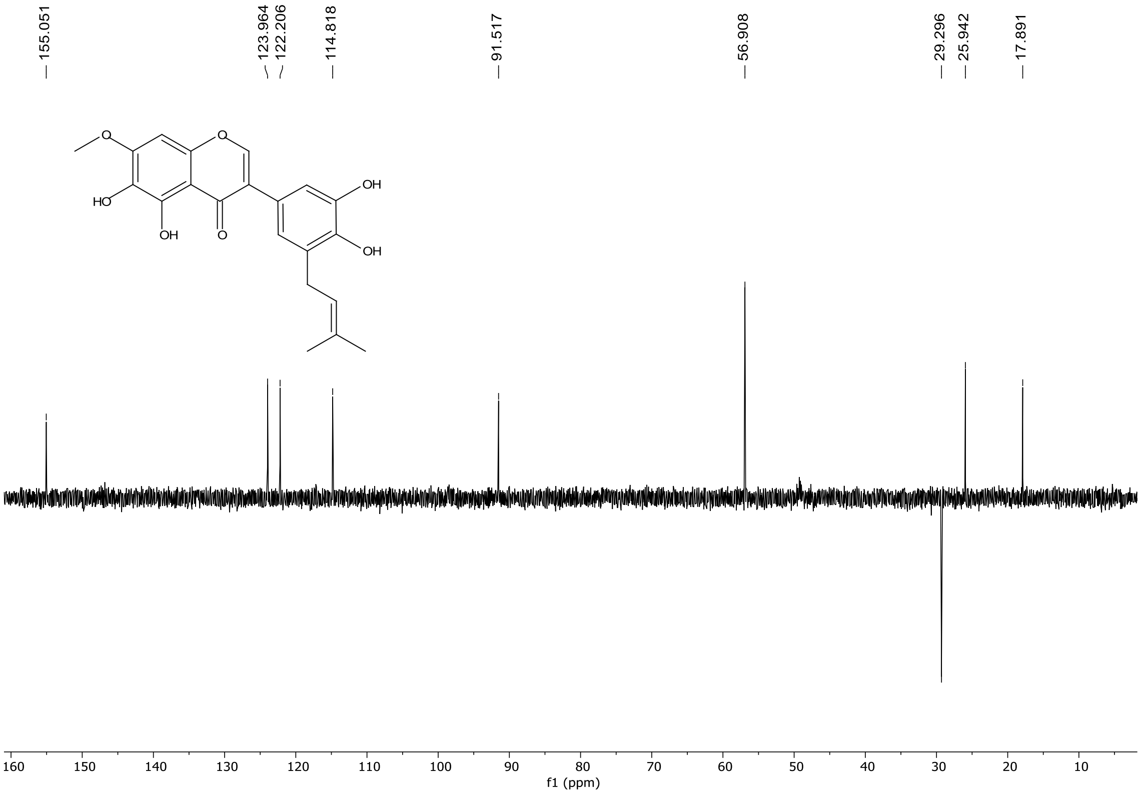

Figure S67. HSQC spectrum of 9 at 500 MHz in MeOD

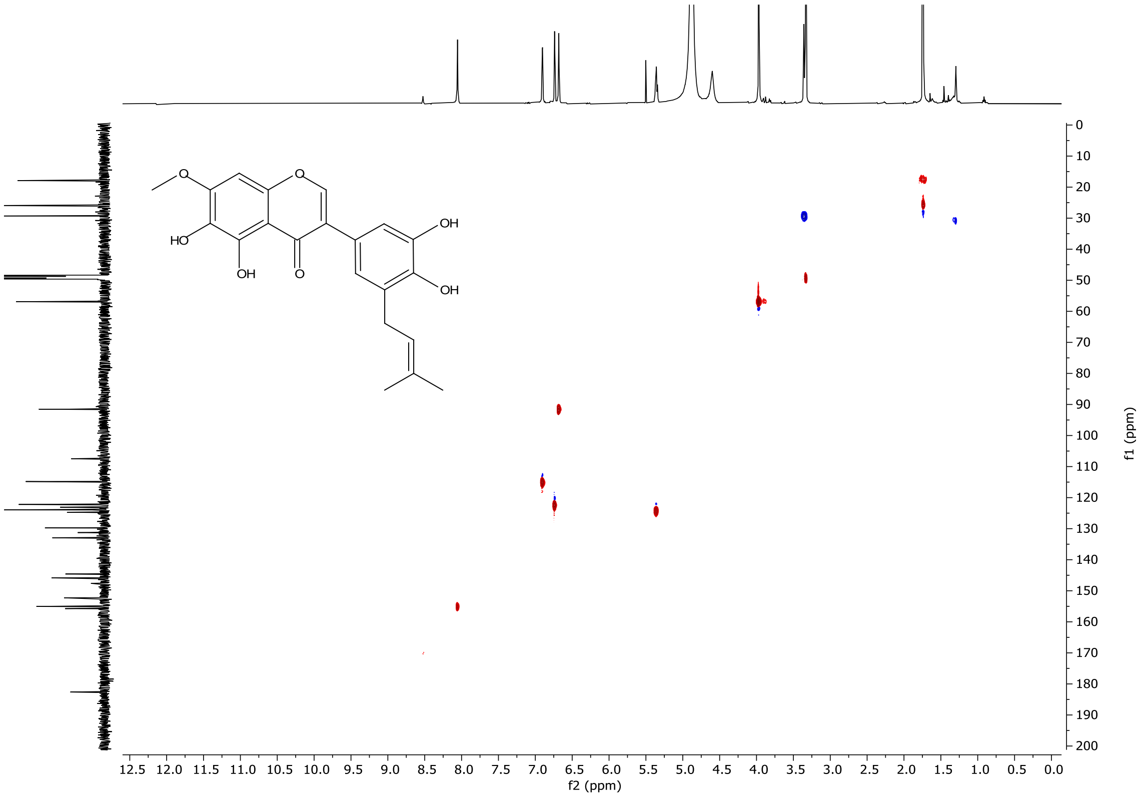

Figure S68. HMBC spectrum of 9 at 500 MHz in MeOD

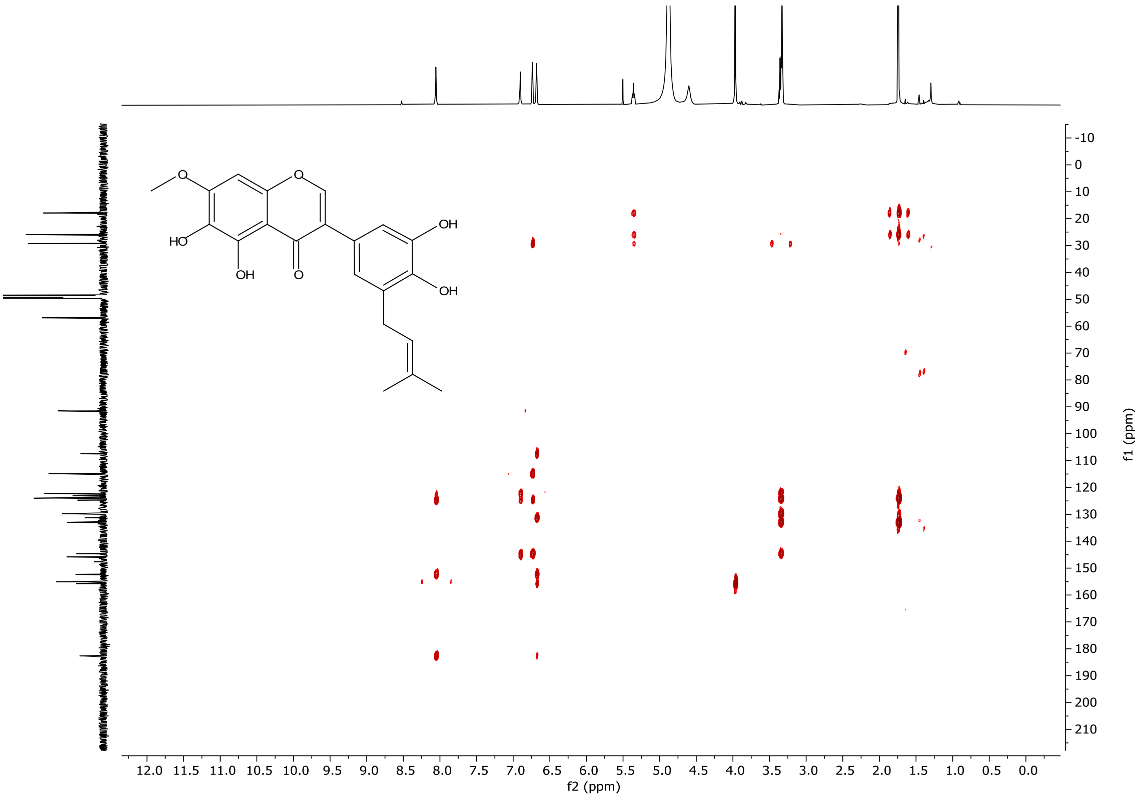

Figure S69. COSY spectrum of 9 at 500 MHz in MeOD

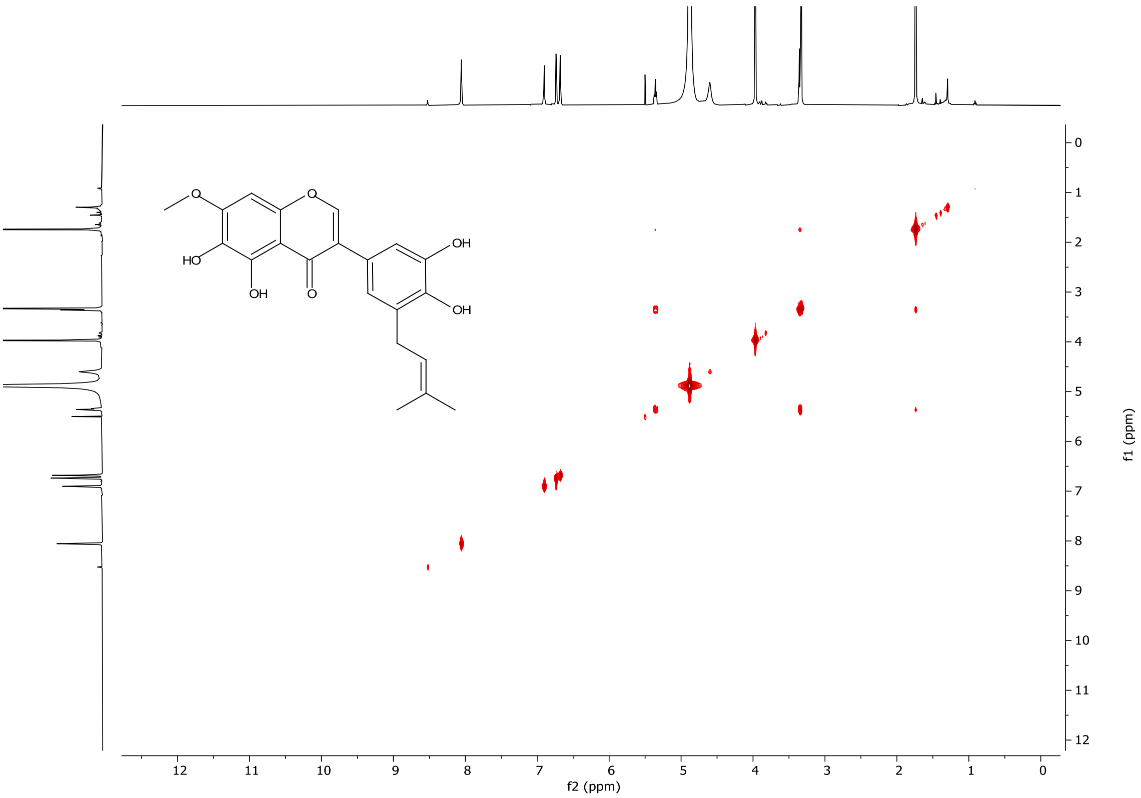

Figure S70. IR spectrum of 9 (liquid solution)

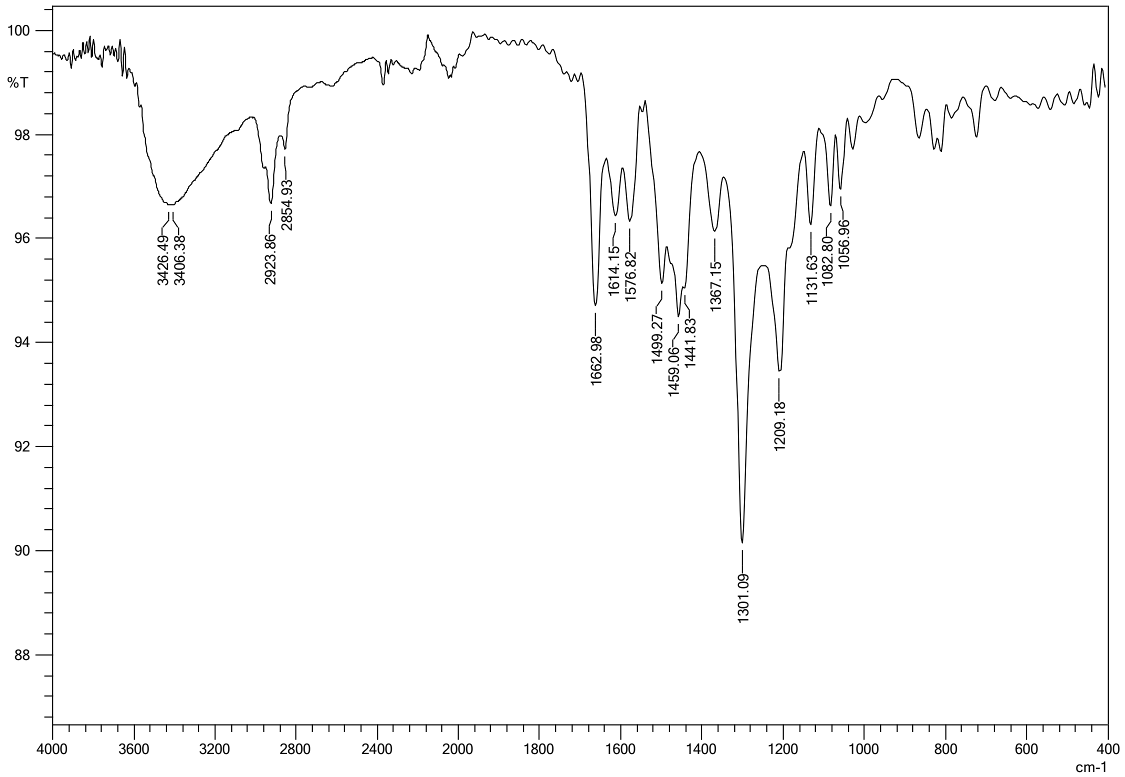

Figure S71. HMBC, ^{1}H-^{1}H COSY and NOESY corrections of compounds from stems of *Acosmium diffusissimum*

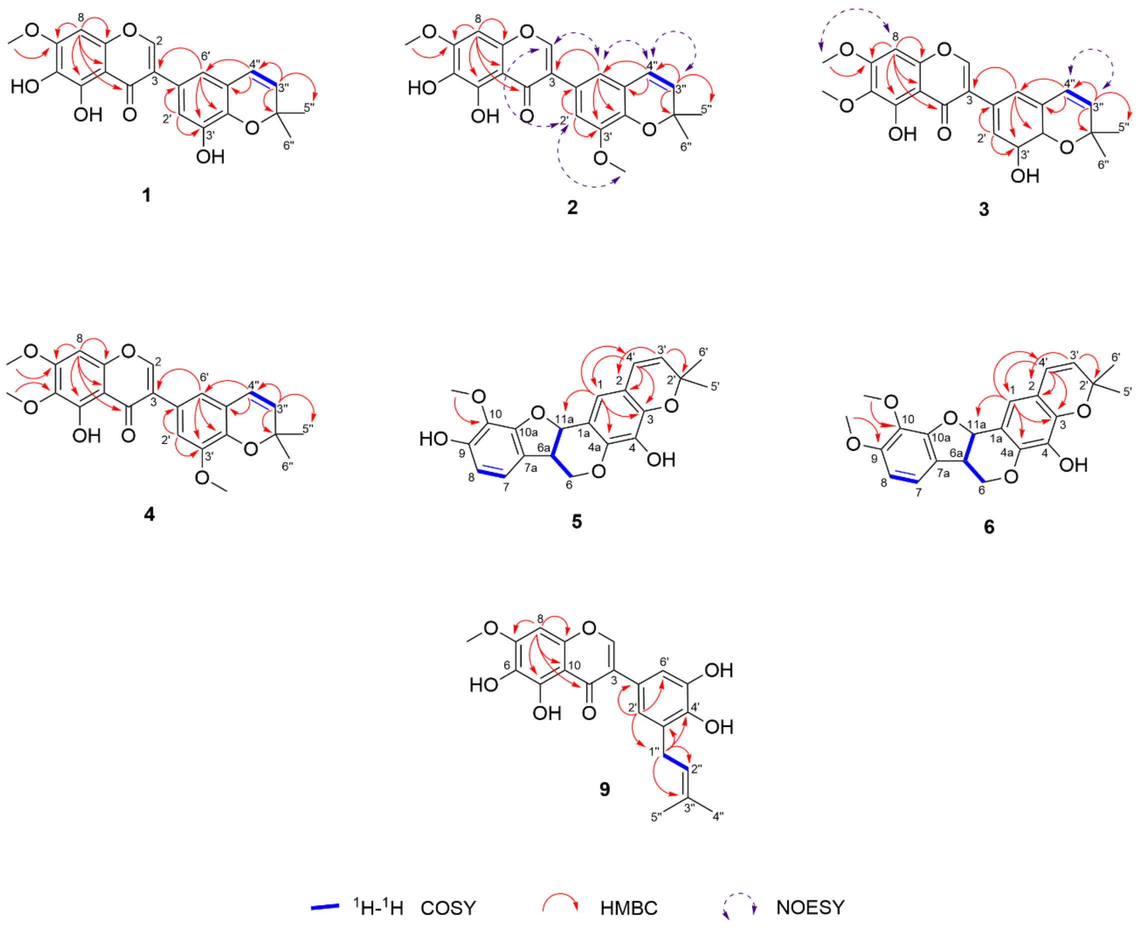

Figure S72. Cell viability of RAW 264.7 macrophages assessed using the MTT method. RAW 264.7 macrophages were exposed to the compounds at different concentrations of 1.25, 2.5, 5, 10 and 20 µg/mL. The results are presented as mean ± standard error of the mean where values for *p<0.05, **p<0.01, ***p<0.001 and ****p<0.0001 when compared to the control group (CTR) were considered significant. The data was analyzed using one-way ANOVA followed by the Bonferroni post-test for comparisons between established groups.

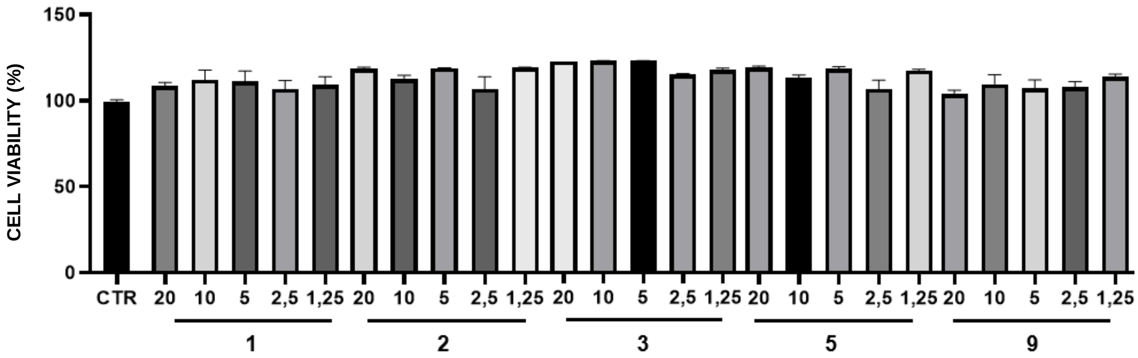

Supplement: Supplementary file 1 — ao5c00866_si_001.pdf [file ao5c00866_si_001.pdf]
